# The BrainMap strategy for standardization, sharing, and meta-analysis of neuroimaging data

**DOI:** 10.1186/1756-0500-4-349

**Published:** 2011-09-09

**Authors:** Angela R Laird, Simon B Eickhoff, P Mickle Fox, Angela M Uecker, Kimberly L Ray, Juan J Saenz, D Reese McKay, Danilo Bzdok, Robert W Laird, Jennifer L Robinson, Jessica A Turner, Peter E Turkeltaub, Jack L Lancaster, Peter T Fox

**Affiliations:** 1 Research Imaging Institute, University of Texas Health Science Center, San Antonio, TX, USA; 2 Department of Psychiatry and Psychotherapy, RWTH Aachen University, Germany; 3 Institute of Neuroscience and Medicine (INM - 2), Research Center Jülich, Jülich, Germany; 4 Department of Physics and Earth Sciences, St. Mary's University, San Antonio, TX, USA; 5 Scott & White Memorial Hospital, Neuroscience Institute, Temple, TX, USA; 6 Texas A&M Health Science Center, College of Medicine, Temple, TX, USA; 7 The Mind Research Network, Albuquerque, NM, USA; 8 Department of Neurology, University of Pennsylvania, Philadelphia, PA, USA

**Keywords:** functional neuroimaging, structural neuroimaging, meta-analysis, BrainMap, neuroinformatics, activation likelihood estimation, ALE

## Abstract

**Background:**

Neuroimaging researchers have developed rigorous community data and metadata standards that encourage meta-analysis as a method for establishing robust and meaningful convergence of knowledge of human brain structure and function. Capitalizing on these standards, the BrainMap project offers databases, software applications, and other associated tools for supporting and promoting quantitative coordinate-based meta-analysis of the structural and functional neuroimaging literature.

**Findings:**

In this report, we describe recent technical updates to the project and provide an educational description for performing meta-analyses in the BrainMap environment.

**Conclusions:**

The BrainMap project will continue to evolve in response to the meta-analytic needs of biomedical researchers in the structural and functional neuroimaging communities. Future work on the BrainMap project regarding software and hardware advances are also discussed.

## Background

A recent and timely editorial in *BMC Research Notes *called for a series of educational articles that promote best practices in data sharing in the biomedical sciences [1]. In the domain of neuroimaging research, data sharing is critical for establishing the robust and meaningful convergence of knowledge of human brain function and structure. The need for such data pooling is primarily dictated by the inherent limitations of neuroimaging data. Most important among those are the rather small sample sizes investigated, the low reliability of indirect signals, and the inherent subtraction logic that is only sensitive to differences between two specific conditions.

Progress towards open sharing of reusable original data has been slow, limited by complex data acquisition and analysis techniques that require extensive curation, the size of the data sets, patient confidentiality, as well as a desire on the investigators' part to protect their costly investment and maintain future rights to their data. Nevertheless, several recent efforts have begun to promote neuroimaging data sharing on a large scale, such as the Biomedical Informatics Research Network [2,3], XNAT Central [4,5], the Alzheimer's Disease Neuroimaging Initiative [6,7], and the Human Connectome Project [8,9]. These projects focus on sharing complete imaging data sets at the subject level across a wide range of modalities, such as task-based functional magnetic resonance imaging (fMRI), resting state fMRI, structural MRI, diffusion imaging, positron emission tomography, magnetoencephalography, and electroencephalography. These multivariate neuroimaging data can be processed and analyzed in a huge variety of ways using algorithms that are in a continual state of evolution and improvement. As a result, understanding complete data and processing provenance [10] across these diverse data sets remains a significant neuroinformatics challenge for the imaging community.

In contrast to these large-scale, multi-institutional sharing initiatives, the BrainMap project was created as an alternative to sharing raw biomedical images. Instead, BrainMap offers a venue for sharing neuroimaging data in a reduced format as a means to encourage and facilitate the identification of consistent findings on brain activity and structure across multiple data sets [11-14]. Here, we describe the rigorous community standards developed since the inception of functional and structural neuroimaging research that have laid the foundation for the advancement of formal meta-analysis methods. These meta-analyses do not require access to raw image data, but can be achieved via information reported by authors in the published literature. In this report, we address the data and metadata standards that enable neuroimaging meta-analyses and the strategy developed by the BrainMap project to encourage data reuse and sharing throughout the community.

### Neuroimaging Data Analysis and Reporting Standards

Spatial normalization algorithms have been developed and implemented in all of the major neuroimaging software packages (e.g., *FSL *[15], *SPM *[16], *AFNI *[17], etc.) to ensure that data from individual subjects are spatially normalized from a subject's "native" brain space to a "standard" brain space. This data standardization removes the effects of intersubject anatomical variability due to differences in brain size and shape, allowing investigators to report their research findings in a manner that facilitates the comparison and synthesis of results across multiple studies [18]. The location of brain imaging results are hence generally published as three-dimensional coordinates (*x*, *y*, *z*) of the centers of mass of clusters or local maxima of brain activation or structural findings, to provide readers with quantitative summaries of the statistical parametric images, with corresponding *z *or *t *statistic values to indicate the strength of the observations. Most commonly, these tables of coordinates refer to locations in Talairach [19] or MNI standard spaces [20]. To facilitate meta-analysis of structural or functional brain findings, it is critical that authors clearly report which standard space was utilized in their publications, as well as which software application was used for spatial normalization, since different applications can yield different results [21]. Frequently, this data description can be incomplete or even inaccurate in the literature, especially when the authors have employed a coordinate conversion algorithm to convert MNI coordinates to Talairach space (or vice versa) [21,22] and do not properly indicate this data transformation. Incomplete data descriptions can have a significant effect on meta-analysis outcomes [23], and can be remedied by stronger adherence to the data reporting standards set forth by the fMRI Methods Working Group [24].

**Figure 1:**
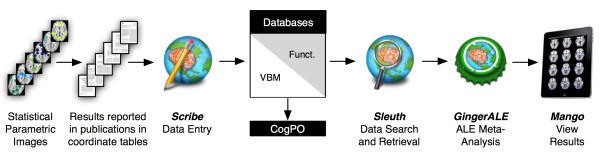
**The BrainMap Procedure for Coordinate-Based Meta-Analyses**. In the human neuroimaging literature, investigators frequently compute a series of statistical parametric images that summarize the group results observed in their functional or voxel-based morphometry neuroimaging experiments. From these images, the coordinates (*x*, *y*, *z*) of the activation clusters (or clusters of structural differences) are extracted and published in tabular format. *Scribe *is used to input these coordinates and the associated metadata for these experiments into the BrainMap functional or VBM databases. Once the entries are inserted into the appropriate database, *Sleuth *is used to search and retrieve coordinates and metadata, and filter the search results to create a data set suitable for meta-analysis. *GingerALE *is used to perform activation likelihood estimation (ALE) meta-analysis of the data, and these results can be viewed in *Mango*, or any similar image viewer. As an ancillary tool, the Cognitive Paradigm Ontology (*CogPO*) has been developed from the BrainMap schema for describing cognitive neuroimaging experiments, and can be used by any researcher to aid in the annotation and formal representation of their own experiments [52].

Capitalizing on these community standards for data analysis and reporting, the BrainMap project was conceived in 1988 and originally developed as a web-based interface to guide users through search, retrieval, and visualization of a coordinate-based database of functional neuroimaging results [25]. After more than 20 years of development, BrainMap has evolved into a much broader project whose software and data have been utilized in nearly 130 publications, with half of those articles published in the last two years [26]. In contrast to other neuroimaging databases, BrainMap provides not only data for meta-analyses and data mining, but also distributes software and concepts for quantitative integration of data. Currently, the BrainMap project includes two neuroimaging databases, three desktop software applications, one web-based application, and several other tools that serve ancillary functions for carrying out meta-analyses. Below we describe recent updates to the project and provide an educational description for performing meta-analyses in the BrainMap environment (an overview is depicted in Figure 1); this information reflects the new software versions that were released in August 2011.

## Findings

### BrainMap Databases

BrainMap was initially developed as a database for *functional *neuroimaging studies reporting brain activation patterns as tabular-formatted three-dimensional stereotactic coordinates. Metadata describing the experimental design and data processing pipeline for each study are manually extracted from each publication, along with the coordinates, by internal BrainMap staff members or external users from the community. Generally, the latter is supported by investigators who are interested in performing a meta-analysis on a given topic and consequently wish to increase BrainMap's volume of studies relevant to this topic. Together, the extracted metadata and coordinate data for each publication comprise the database content. Currently, BrainMap archives the results of 2,114 functional neuroimaging publications, which include 39,672 subjects and report 79,577 activation locations across 9,994 experiments. This volume is estimated to include approximately 20% of the relevant literature [27].

In 2007, the BrainMap team initiated efforts to expand into archiving *structural *neuroimaging data. Specifically, voxel-based morphometry (VBM), which is a statistical analysis for investigating structural differences between two groups of subjects (e.g., areas of increased gray matter density for patients vs. healthy controls), also had achieved community standardization such that results reported in the form of stereotactic coordinates had become the norm in the same form as for functional neuroimaging data. Formal integration by meta-analyses on structural neuroimaging findings has thus become possible [28-30]. Following multiple years of data entry, as well as database, servlet, and software programming and development, the BrainMap VBM database has been released to the public and is now live. Currently, BrainMap VBM archives the results of 729 voxel-based morphometry publications, which includes 50,375 subjects reporting 15,206 locations of structural differences across 2,231 experiments. While the BrainMap VBM database is much smaller than the functional database, this volume of the literature is also much smaller (939 total eligible publications), and it is hence estimated that the database includes approximately 78% of the eligible VBM studies.

### Database Infrastructure

Both the BrainMap functional and VBM databases are managed with Oracle, a commercial relational database management system [31]. BrainMap's data resides on a Sun Microsystem workstation running Oracle Solaris 10 at the Research Imaging Institute in San Antonio, TX. The Oracle Corporation's Object-Relational Database Management System was utilized when designing BrainMap's database structure. Object-relational databases have a high-level structure that allows for defining data as objects instead of a collection of items in tables. Treating objects as cohesive units simplifies storing, updating, and retrieving data, as well as defining relationships between objects. The ability to quickly fetch object data is extremely helpful when communicating with BrainMap's Object-Oriented client programs.

### BrainMap Software Applications

BrainMap's client programs are written in the Java programming language that may run under PC, Macintosh, and UNIX operating systems. The use of Java makes updating and distributing these applications simpler for both developers and users. There are three main desktop applications that provide access to the BrainMap databases: *Scribe*, *Sleuth*, and *GingerALE*.

1. ***Scribe ***allows users to input data and metadata from publications into the databases using the BrainMap taxonomy [32]. In previous versions, *Scribe *only provided access to functional submissions; however, the newly released version 2.0 allows users to create entries for either the functional or VBM databases. When *Scribe *is launched, a dialog window asks users to select which type of paper they wish to code, functional or VBM. Following this, the main application window is configured to match the user's selection. Functional submissions are created as .ent files while the VBM database archives .vbm files, which allows each type of submission to be easily identified. In addition to integrating the interface for functional and VBM submissions, we have also improved how the application functions when there is no active internet connection, as this caused some problems in previous software versions.

2. ***Sleuth ***allows users to search and retrieve coordinate data and metadata from the databases. A radio button gives users the option of searching either the functional or VBM database; simultaneous searching of both databases is not permitted to avoid a conflation of both types of imaging results. The graphical user interface of *Sleuth *has been redesigned in version 2.0, and now allows users to build searches with multiple criteria using an interface that was inspired by the playlist building feature in Apple's *iTunes *software [33]. In addition, searches are now more rapidly executed as a result of server side optimizations to the database architecture in which search results are pre-generated instead of being generated dynamically each time a search is made. Once a search is executed, users are able to examine and filter the query results in workspace panel. As part of this process, the software also allows the brain-based visualization of results with individual experiments being toggled on and off. Most recently, *Sleuth *now offers the ability to search for studies identifying functional or structural results located in a three-dimensional arbitrary-shaped region of interest (ROI) in Talairach or MNI space. To carry out these image-based ROI searches, all user-originated files must conform to a strict format: ROIs must be formatted as binary NIfTI [34] images with 1x1x1 mm^3 ^resolution, and the ROI must not extend across more than 500 voxels. These stringent requirements are enforced to ensure a timely response from the database; more advanced hardware solutions are currently being evaluated to reduce these technical limitations and allow rapid image-based ROI searches of greater volume. Other *Sleuth *tools include the ability to: (1) generate a histogram of metadata results that describe the paradigms and behavioral domains associated with experiments in the current workspace, and (2) export workspaces in multiple formats, including images in NIfTI format (nii), *EndNote *[35] citation files (txt), or files suitable for meta-analysis using the *GingerALE *application (txt). Meta-analysis coordinate files can be exported in the form of either Talairach or MNI coordinates.

3. ***GingerALE ***allows users to carry out activation likelihood estimation (ALE) meta-analyses using BrainMap-formatted coordinate-based data in Talairach or MNI space. In ALE, a set of coordinates retrieved via *Sleuth*, which are identified by the user as suitable for meta-analysis, are input to *GingerALE*, blurred with a Gaussian distribution to accommodate the associated spatial uncertainty, and a statistical parameter is computed that estimates convergence across the modeled brain images and measures the likelihood of activation at each voxel in the brain. ALE was originally developed by Turkeltaub et al. [36], but the algorithm has undergone several revisions since then. When initially integrated into the BrainMap environment, a statistical framework was developed for multiple comparisons corrections and allowing two sets of coordinates to be contrasted [37]. In 2009, the algorithm was extensively modified to: (1) model the spatial uncertainty of each brain location using an estimation of the intersubject and interlaboratory variability typically observed in neuroimaging experiments, and (2) calculate the above-change clustering between experiments (i.e., random-effects analysis), rather than between foci (i.e., fixed-effects analysis) [38]. Most recently, we published a modification of the ALE algorithm that minimizes both within-experiment and within-group effects, further optimizing the ALE technique [39]. These algorithms are available in the newest software release, *GingerALE *version 2.1. *GingerALE *2.1 also includes a more streamlined interface and a revision of the subtraction analysis [40] originally developed by Laird et al. [37] that has been substantially improved using the new statistical framework developed by Eickhoff et al. [38]. Tools are also included to spatially renormalize coordinates to Talairach or MNI space using publicly available algorithms [21,22].

All of the above software applications can be downloaded from the BrainMap website [41]. [See Additional File 1 for the *Scribe *user manual, Additional File 2 for the *Sleuth *user manual, and Additional File 3 for the *GingerALE *user manual]

### Related Tools

In addition to *Scribe*, *Sleuth*, and *GingerALE*, BrainMap distributes or links to several other related tools that serve ancillary functions for carrying out coordinate-based neuroimaging meta-analyses and sharing coordinate-based data and metadata.

1. ***BrainMapWeb ***is a web-based application for searching and retrieving data from the functional database [42]. Queries are similar to those of *Sleuth*, but lack 3D visualizations and advanced data manipulation capabilities.

2. ***icbm2tal ***is a coordinate-based transformation that was developed to accommodate spatial disparity between Talairach and MNI coordinates [21]. *icbm2tal *has been shown to provide improved fit as compared to the earlier *mni2tal *transform [22], and improve the accuracy of coordinate-based meta-analyses [23]. *icbm2tal *is distributed from within *GingerALE *or can be downloaded as MATLAB .m files [43].

3. ***Mango ***(Multi-image Analysis GUI) is a viewer for biomedical research images [44]. It provides analysis tools and a user interface to navigate image volumes. *Mango *is available as a desktop application, web application, or iPad application. In the context of the BrainMap project, *Mango *may be used for viewing meta-analysis results and generating and editing ROIs for *Sleuth's *image-based ROI searches.

4. The ***Talairach Daemon ***is a spatially comprehensive set of anatomical labels for Talairach coordinates [45,46]. The *Talairach Daemon *is available as a desktop java client, web applet, or high-speed database server [47]. The *Talairach Daemon *is utilized by BrainMap to apply anatomical labels to coordinates archived in the databases and to label the centers of mass of ALE meta-analysis results in *GingerALE*.

5. The **Anatomy Toolbox **is a MATLAB-based software tool [48] that allows the comparison of statistical images, including meta-analysis results, with probabilistic cytoarchitectonic maps of the human brain [49,50]. Consistent findings from neuroimaging may be related to the histological properties of the cerebral cortex. In turn, regions of interest defined by cytoarchitectonic areas [51] may be used for probing the BrainMap databases.

6. ***CogPO ***[52] is an ontology of cognitive paradigms that is being built to enable the formal, machine-interpretable representation of paradigms in cognitive neuroscience experiments [53]. *CogPO *is based on the BrainMap taxonomy for describing experiments, and utilizes both the BrainMap functional database and the Functional Imaging Biomedical Informatics Research Network Human Imaging Database [54] for development and evaluation. *CogPO *version 1 is available as a wiki [55] or can be downloaded in OWL format [56]. *CogPO *also is available from within the Neuroscience Information Framework *NeuroLex *Wiki [57] and the National Center for Biomedical Ontology *BioPortal *[58].

### Two Exemplar Meta-Analyses

In this section, we provide two examples of how coordinate-based neuroimaging meta-analyses can be performed using the BrainMap system of databases and software applications.

#### ALE Meta-Analysis of Acupuncture Studies

**Figure 2:**
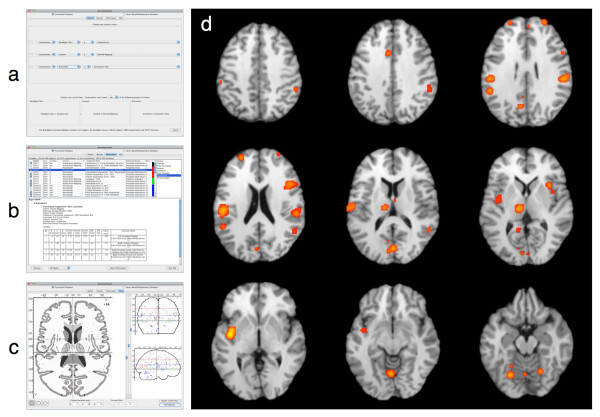
**Procedure and Results for a Paradigm-Based ALE Meta-Analysis**. In paradigm-based, or function-based, meta-analyses, the BrainMap database is searched for a paradigm or task of interest by (a) constructing an appropriate set of search criteria within *Sleuth*. Studies matching this query are (b) downloaded to *Sleuth's *workspace panel for further filtering, and (c) the observed location results of these experiments can be visualized on a glass brain. Using GingerALE, these locations can be meta-analyzed using the ALE approach, and (d) the ALE results can be visualized using *Mango*.

Paradigm-based, or function-based, meta-analyses are analyses in which coordinates are pooled from a set of published neuroimaging studies examining similar behavioral conditions, as a means to determine the most consistently observed activation pattern for a given task. As an example, we performed a paradigm-based meta-analysis to identify consistent results observed during acupuncture tasks in functional neuroimaging studies. A *Sleuth *search for the experiments reporting activations in healthy subjects was constructed using multiple search criteria: (1) Experiments: Paradigm Class IS "Acupuncture", (2) Experiments: Context IS "Normal Mapping", and (3) Experiments: Activation IS "Activations Only". This *Sleuth *query returned hits for 10 papers, with 23 experiments; however, the search results were then manually filtered in *Sleuth's *workspace by toggling experiments to remove activations associated with sham acupuncture. Thus, the final meta-analytic data set included 180 coordinates of brain activation locations from 10 papers across 20 experiments. These coordinates were exported from *Sleuth *for ALE meta-analysis using *GingerALE*. *GingerALE *also accepts text files generated manually by the user (i.e., without *Sleuth*); however, the formatting must match *Sleuth's *output. [See Additional File 4 for an example of a BrainMap-formatted text file that can be read by *GingerALE*] Figure 2 depicts the procedure for (a) searching, (b) filtering, and (c) visualizing the acupuncture workspace in *Sleuth*, and (d) reveals the results of ALE meta-analysis of this data set as viewed in *Mango*. The strongest convergence of foci from this group of acupuncture studies was observed in the bilateral insula, postcentral gyri, inferior parietal lobule, thalamus, and cerebellum. These regions are generally associated with stimulation of the somatosensory system, and are likely candidates for regions engaged during acupuncture tasks.

#### Meta-Analytic Connectivity Modeling of the Amygdala

In contrast to a paradigm-based meta-analysis, we next demonstrate how structure-based meta-analyses are carried out using BrainMap software using the meta-analytic connectivity modeling (MACM) approach. MACM was developed as a method of investigating whole-brain coactivation patterns for a region of interest across a range of tasks, i.e., functional connectivity. In this technique, the BrainMap database is used to search for studies reporting normal mapping activations in healthy subjects that fall within the boundaries of a three-dimensional rectangular, spherical, or arbitrary-shaped ROI, regardless of the behavioral conditions employed. The whole brain activation patterns from these studies are then integrated using the ALE method, yielding a map of significant coactivations that provides a task-free meta-analytic model of the region's functional interactions throughout the rest of the brain. This method can be viewed as the meta-analytic analogue to seed-based connectivity analyses of resting state fMRI data [59-61]. MACM analyses have been shown to be useful in understanding the functional connectivity of the amygdala [62], parietal operculum [63], regions of the default mode network [64], and the nucleus accumbens [65].

**Figure 3:**
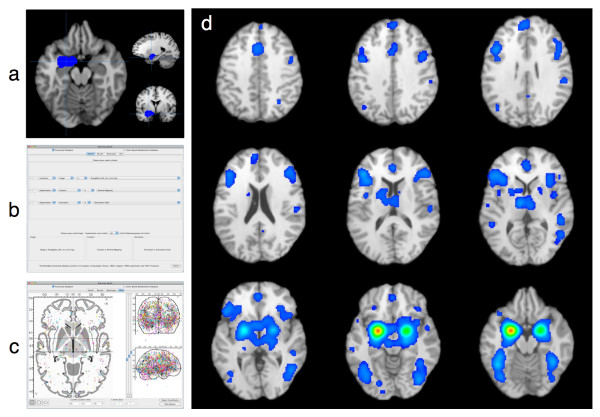
**Procedure and Results for a Meta-Analytic Connectivity Modeling Analysis**. In meta-analytic connectivity modeling (MACM) analyses, the BrainMap database is searched for activations in healthy subjects that are reported within the boundaries of a three-dimensional rectangular or arbitrary-shaped ROI. To identify the regions that coactivate with this ROI, a user must (a) obtain or generate a gzipped NIfTI image file that identifies the desired region of interest, and (b) construct an appropriate set of search criteria within *Sleuth*. Studies matching this query are downloaded to *Sleuth's *workspace and (c) the observed locations reporting across these experiments are visualized on *Sleuth's *glass brain. After meta-analysis using *GingerALE*, (d) the MACM results can be visualized in *Mango*.

As an example, we performed a MACM analysis of the left amygdala, using an ROI defined from the Harvard-Oxford Structural Probability Atlas distributed with the *FSL *software [15,66] and converted to Talairach space. This ROI was utilized by Robinson et al., although in that study the ROI was thresholded to 70% probability [62]. A *Sleuth *search for the experiments reporting activations in healthy subjects was constructed using multiple search criteria: (1) Locations: Talairach Image IS "LeftAmygdala.nii.gz", (2) Experiments: Context IS "Normal Mapping", and (3) Experiments: Activation IS "Activations Only". [See Additional File 5 for an example of a BrainMap-formatted ROI image file of the left amygdala that can be used for image-based ROI searches in *Sleuth*] This *Sleuth *query returned hits for 188 papers, with 263 experiments and 3,305 locations matching the search criteria; all of these coordinates were exported as a text file to be meta-analyzed with *GingerALE*. Figure 3 depicts (a) the visualization of the left amygdala ROI in *Mango*, (b) the search criteria in *Sleuth*, (c) visualization of the left amygdala workspace in *Sleuth*, and (d) the results of the *MACM *analysis of this data set in *Mango*. As reported by Robinson et al. [62], the strongest convergence of foci of left amygdala coactivations was observed in the bilateral amygdala, posterior and anterior cingulate, inferior and medial frontal gyri, insula, thalamus, and fusiform gyri.

## Conclusions

The BrainMap project, including its databases, software clients, and other associated tools, will continue to evolve in response to the meta-analytic needs of biomedical researchers in the structural and functional neuroimaging communities. Current work on BrainMap focuses on further extensions of the analysis capabilities, such as adding conjunction analysis of multiple data sets to *GingerALE *[67] and the ability to run a cluster analysis on a statistical parametric image. Tools are also being developed to integrate BrainMap metadata in *Mango *to facilitate detailed and quantitative functional labeling of any given image in the viewer (e.g., a single region of interest or multiple regions from an activation map). In addition, we are currently working on adding capabilities for carrying out statistical testing of *Sleuth's *metadata histograms to determine the significant behavioral domains or paradigms that have been most frequently reported as corresponding to a given ROI or set of ROIs. In the next year, *BrainMapWeb *will also undergo a substantial upgrade that will include a more efficient interface and integration of VBM searches. Lastly, we aim to update the hardware that serves the BrainMap database, in order improve the computational speed of image-based ROI searches in *Sleuth *and reduce the technical limitations that have been imposed, particularly with respect to the size of the ROIs allowed.

The BrainMap project's overall goal is to provide the human brain mapping community with data sets, computational tools, and neuroinformatics resources that enable quantitative meta-analyses and meta-analysis-based neuroimaging data interpretation. Our philosophy is that the most compelling meta-analytic applications are those extend the ALE method beyond that of a purely retrospective tool and utilize meta-analytic results to guide prospective analyses in newly acquired experimental neuroimaging data. Our aim is to fully embrace this philosophy in the next phase of the BrainMap project as we develop novel meta-analytic tools for improving causal model fit when studying the temporal dynamics that are engaged across different brain regions using effective connectivity techniques, such as dynamic causal modeling [68] and structural equation modeling [69]. Similarly, our most recent work emphasizes our meta-analytic philosophy via large-scale data mining as a means to investigate fundamental brain-behavior correlations and the organization and interactions within intrinsic connectivity networks [70,71]. Future work on the BrainMap project will additionally involve the development of more comprehensive data mining techniques, as well as extending the functionality of meta-analytic connectivity mapping tools, including constructing, validating, and distributing an atlas of whole-brain task-dependent connectivity. Within the scope of these future aims, the BrainMap project hopes to achieve significant progress in our long-term vision to provide researchers with the tools and data that will provide the foundations for neuroimaging-based models of healthy brain function, as well as models of psychiatric or neurological disease, across the human lifespan.

## Competing interests

The authors declare that they have no competing interests.

## Authors' contributions

ARL and PTF designed the study. ARL wrote the manuscript. SBE, PMF, AMU, and JLL developed and managed the software applications, databases, and other resources. SBE, DB, and PET contributed algorithms and tools to the software. KLR, JJS, DRM, JLR, and RWL contributed and analyzed data and tested the software. All authors read and approved the final manuscript.

## Supplementary Material

Additional file 1 **BrainMap *Scribe *Software Manual**. This file describes the features of the *Scribe *desktop application for creating BrainMap database entries. Click here for file

Additional file 2 **BrainMap *Sleuth *Software Manual**. This file describes the features of the *Sleuth *desktop application for searching, retrieving, and visualizing data archived in the BrainMap databases. Click here for file

Additional file 3 **BrainMap *GingerALE *Software Manual**. This file describes the features of the *GingerALE *desktop application for performing activation likelihood estimation (ALE) meta-analyses on coordinate-based neuroimaging data. Click here for file

Additional file 4 **BrainMap *GingerALE *Coordinate File**. This file is an example of a BrainMap-formatted text file of coordinates that can be read and analyzed using *GingerALE*. Click here for file

Additional file 5 **Gzipped NIfTI Image File of the Left Amygdala for Arbitrary-Shaped ROI Search in *Sleuth***. This file is an example of a BrainMap-formatted image file that can be used for arbitrary-shaped ROI searches in *Sleuth*. Click here for file
