# Supplementary material for: The BrainMap strategy for standardization, sharing, and meta-analysis of neuroimaging data
Source: BMC Res Notes. 2011 Sep 9;4:349. doi: 10.1186/1756-0500-4-349 (PMC3180707; doi:10.1186/1756-0500-4-349)

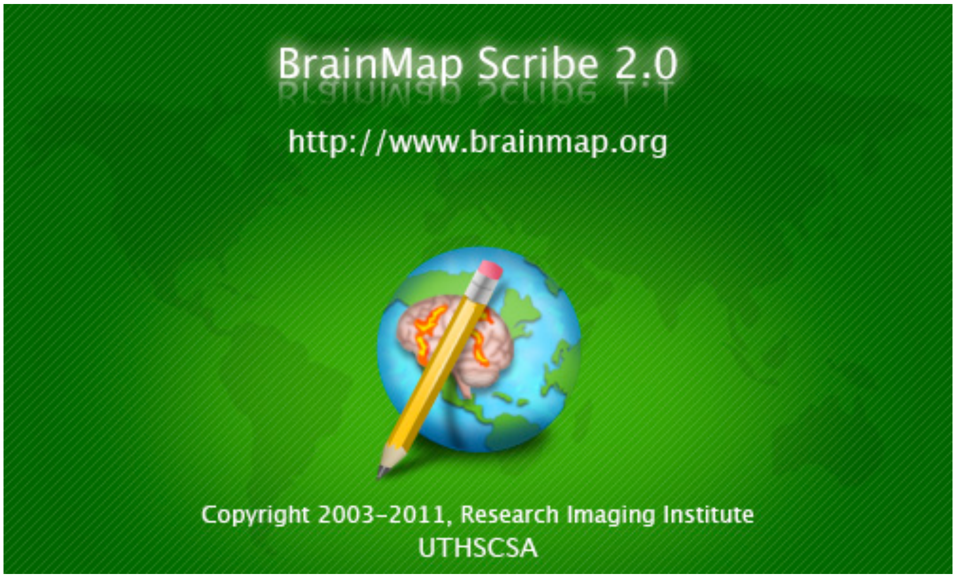

User Manual for Scribe 2.0

http://brainmap.org

Angela R. Laird, Ph.D.
Research Imaging Institute, UT Health Science Center San Antonio

BrainMap Development Team:
Peter T. Fox, M.D.
Angela R. Laird, Ph.D.
Simon B. Eickhoff, M.D.
Jack L. Lancaster, Ph.D.
Mick Fox, Programmer Analyst
Angela M. Uecker, Programmer Analyst
Kimberly L. Ray, Research Assistant,
Juan J. Saenz, Jr., Research Assistant

Updated 20 May 2011

Introduction

BrainMap has been in step-wise development since 1988 and actively in use since 1992. Its purpose is to facilitate the retrieval and understanding of the literature on functional mapping of the human brain, and to enable meta-analysis of this literature. The structure of BrainMap data entry involves three levels of information: paper, experiment, and locations (coordinates). Paper-level information contains fields such as authors, year of publication, and age of subjects. In the BrainMap database, experiments are defined as the comparison of two (or more) imaged conditions that result in a statistical parametric image (SPI). Papers containing multiple experiments require information for each experiment to be entered separately; this includes fields such as the paradigm class. At the location level, Talairach or MNI (x,y,z) coordinates (i.e., centers-of-mass of sites of activation) are extracted from the SPIs and are entered into the database.

Initially, the BrainMap database archived only functional neuroimaging experiments. In 2007, the BrainMap team initiated efforts to expand into archiving *structural* neuroimaging data. Specifically, voxel-based morphometry (VBM), which is a statistical analysis for investigating structural differences between two groups of subjects (e.g., areas of increased gray matter density for patients vs. healthy controls), also had achieved community standardization such that results reported in the form of stereotactic coordinates had become the norm in the same form as for functional neuroimaging data. Formal integration by meta-analyses on structural neuroimaging findings has thus become possible. Following multiple years of data entry, as well as database, servlet, and software programming and development, the BrainMap VBM database has been released to the public and is now live (June 2011).

The Scribe software application is used to code data and meta-data from a functional or voxel-based neuroimaging paper so that this information can be submitted and inserted into the BrainMap database. When Scribe is launched, a dialog window asks users to select which type of paper they wish to code, functional or VBM. Following this, the main application window is configured to match the user's selection. Functional submissions are created as .ent files while the VBM database archives .vbm files, which allows each type of submission to be easily identified.

Regardless of the type of submission, the first panel of the BrainMap Scribe interface is entitled 'Citation,' and the last is entitled 'Feedback.' The interface is designed to enter data from the left to right. Some entry panels will not accept information until previous panels have been completed. Furthermore, all panels except 'Experiment' require paper-level information. Some fields provide drop-down menus and some provide windows for free-form entry. The words from the drop-down menus are all keywords that users may use to conduct searches using BrainMap Sleuth (www.brainmap.org/sleuth).

According to BrainMap's system of describing a paper, studies are broken down into **Conditions** and **Experiments**.

Conditions:
Subjects perform tasks under certain conditions and these conditions are contrasted in an experiment. Each condition used in a BrainMap-defined experiment should be briefly described and named by simple terms such as "Read", "Finger Tapping", "Rest", or "Word Generation". For example, in a simple motor study, subjects performed two conditions: "Finger Tapping" and "Rest". Conditions are worded in the past tense.

Experiments:
An experiment is the result of comparing two or more conditions. Each experiment name should indicate which conditions were contrasted. In the preceding example, the experiment would be named: "Finger Tapping –

Rest". The names used here should be entered on the Experiments panel. Experiments should be listed here and in the order in which they were published.

Do not enter conditions that do not result in activations or were not used in experiments.

Save BrainMap Database Submission:
It is a good idea to save your changes to file after completing each major panel. To do so, click on the top left program menu: 'File' → 'Save As'. This saves the information in an *.ent file (or a *.vbm file, depending on your choice). Enter the file name in the following format: "Author_Journal_Year.ent". If you are on a PC, be sure and type in file extension. Journal names should be abbreviated and years listed as the last two digits of the published year, for example, "Lee_HBM_02.ent"

Functional or VBM Database Submissions

When Scribe is launched, you will see the following dialog window:

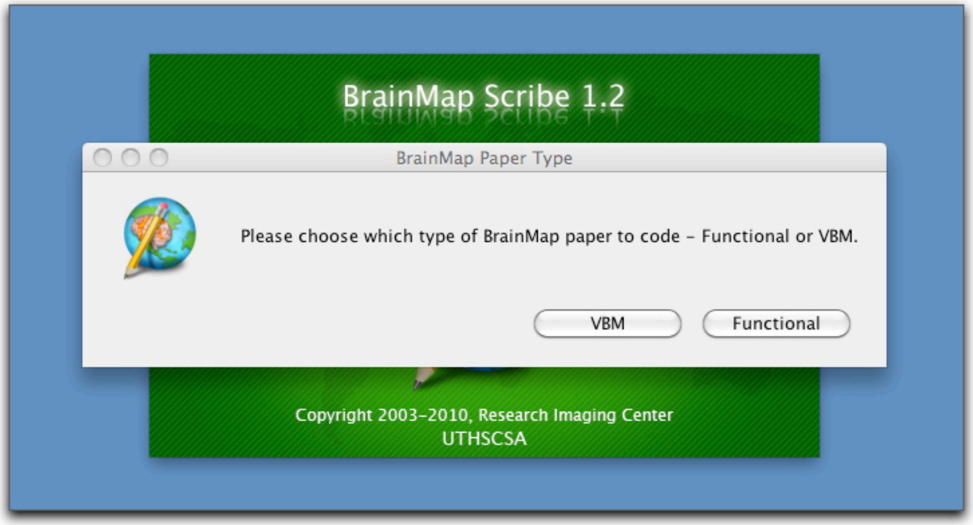

Selecting "VBM" takes you to the interface for a VBM submission, while selecting "Functional" will take you to the interface for a functional submission.

In this manual, we will first describe how to generate a submission for the BrainMap functional neuroimaging database. On page 36, we address the modifications necessary to generate a BrainMap voxel-based morphometry submission.

Functional Neuroimaging Submissions

Citation

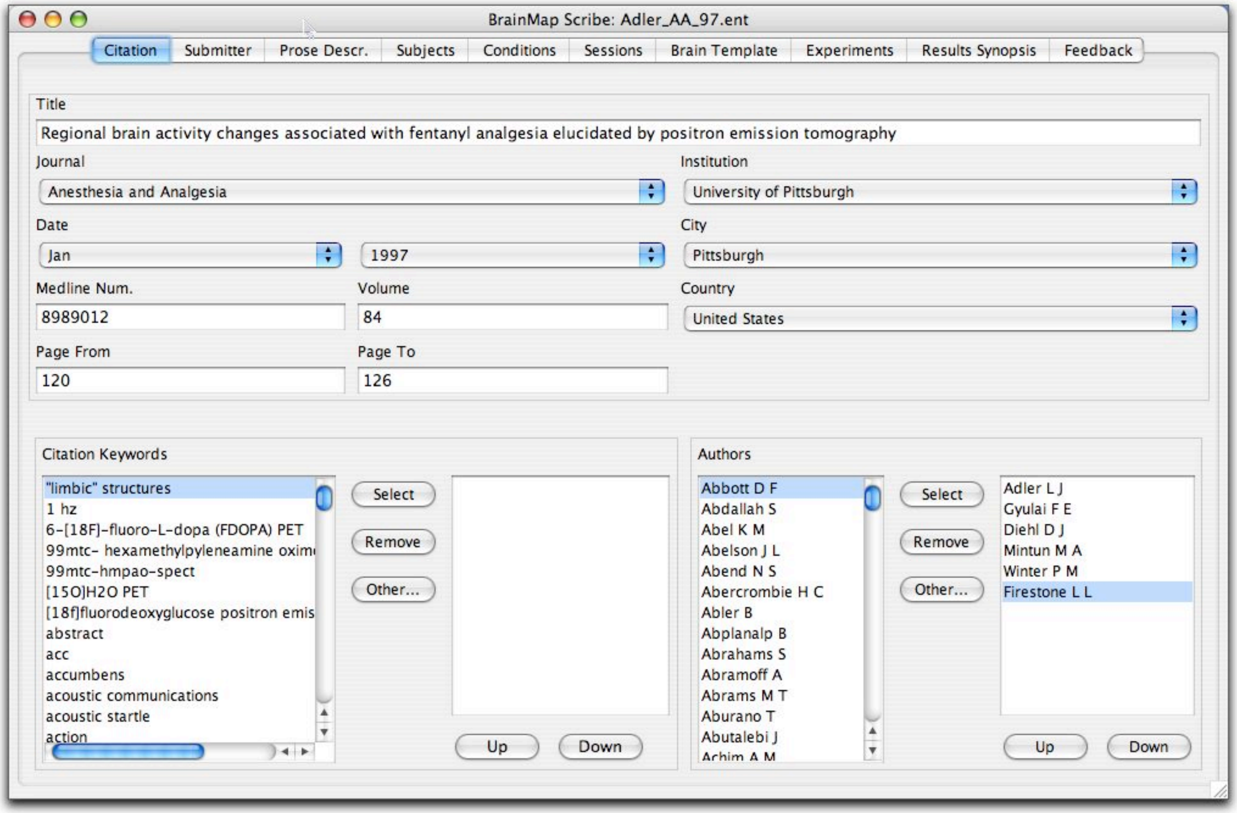

<u>Title</u>

Capitalize the initial word and those used to capitalize proper names; use lowercase for all other words. Also, capitalize the first letter after a colon. Do not leave a period at the end of the title.

<u>Journal</u>

Select the journal name from the pull-down menu provided. If the journal name does not appear, choose 'Other' and enter the journal name without abbreviations. Do not list sections.

<u>Institution</u>

List the name of the institution where the data was acquired. Enter the university name, not the department.

- Enter Institute of Psychiatry, King's College of London as: University of London.
- Enter Wellcome Department of Cognitive Neurology as: University College London.

<u>Medline Num.</u>

The Medline Number can be located at the PubMed or Medline website. If a paper is not archived in PubMed, leave this field blank.

<u>Citation Keywords</u>

Click on the desired keywords (from the list published with the paper), and then click the 'Select' button. The chosen keyword will appear in the box to the right. Use the 'Up/Down' buttons to reorganize the keywords as needed.

If the appropriate keywords used in your paper are not included in the list provided, click the 'Other' button and type in the keyword in the window provided.

- Enter the exact keyword listed, for example, fMRI and functional MRI are two different keywords.
- Do not use capital letters except for proper names/nouns.
- **Enter only the keywords published with the paper.** If there are no keywords listed, leave this field blank.

Authors

Enter all authors listed on the paper.

- Do not add another version of an author's name that already appears in the database, e.g., Friston K and Friston K J.
- List authors in the order of appearance on the paper.
- Do not hyphenate first names of authors. Example: Marie-Claude Antoinette should be entered as "Antoinette M C", not as "Antoinette M-C".

Submitter

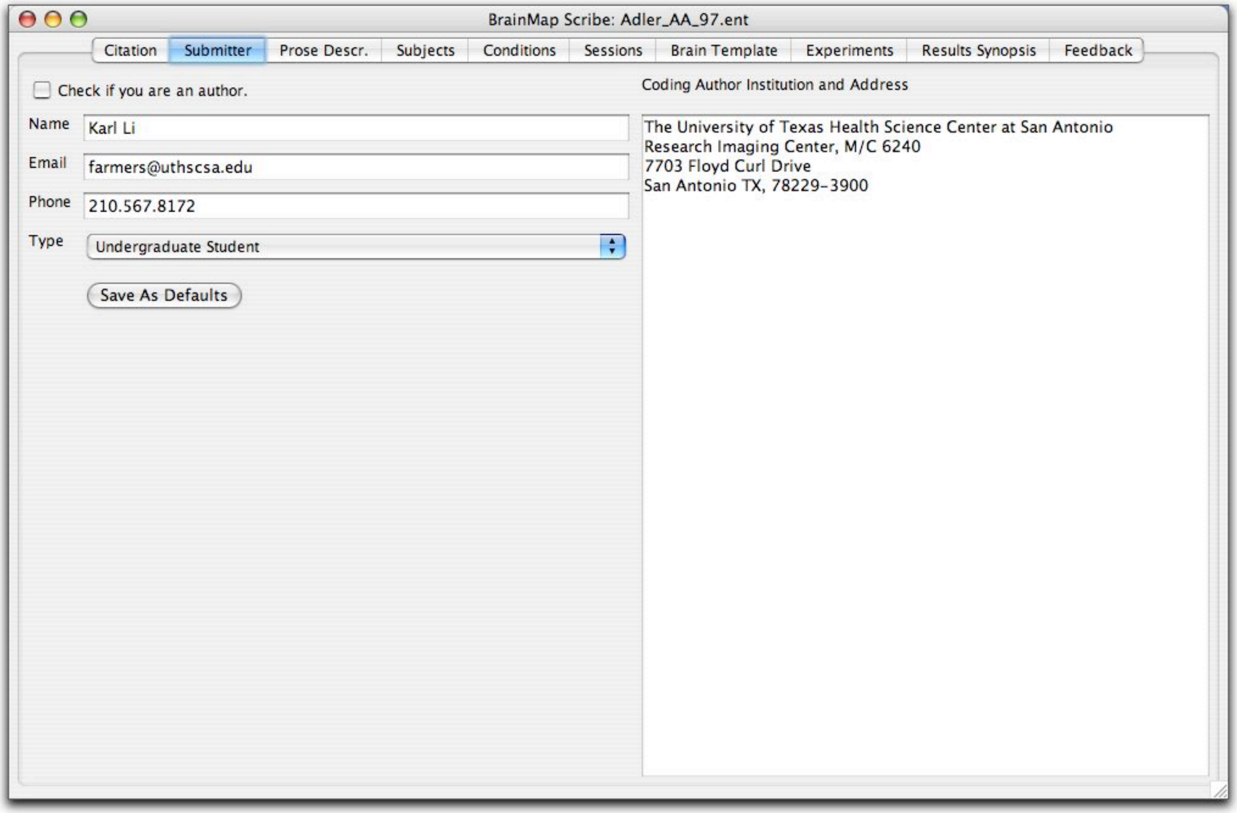

Enter the requested information about the submitter (you). Check the appropriate box if you are an author on the paper. By clicking the “Save as Defaults” button, you can save your submitter information so that you will not have to re-type it on subsequent submissions.

Prose Description

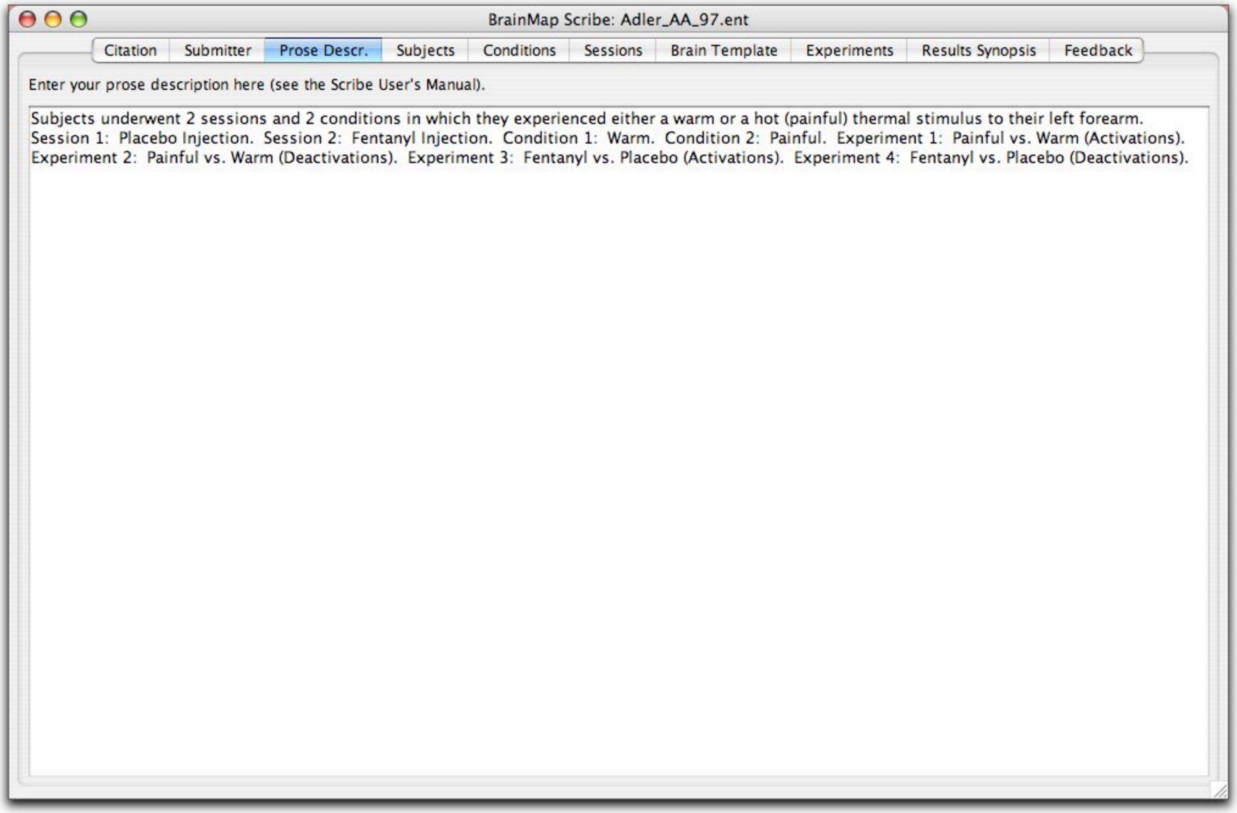

When entering a paper into BrainMap, special care should be taken when writing the Prose Description.

The prose description should:

- Describe only the experimental design (no background, results or discussion)
- Be illustrative, but as succinct as possible
- Be written in the past tense
- Include sufficient, specific information for each condition and experiment so that a reader will fully understand the experimental design in the paper.

Correct Format:

Subjects underwent 2 conditions. Condition 1: Name C1: description. Condition 2: Name C2: description. Experiment 1: Name E1. Experiment 2: Name E2.

Sample Prose Description:

Subjects underwent 2 conditions. Condition 1: Verb Generation: subjects viewed pictures and generated semantically related verbs of the objects. Condition 2: Rest. Experiment 1: Verb Generation - Rest.

- Be as brief as possible when describing conditions.

- Describe the conditions in a chronological sense. For example, "subjects viewed a series of letters; after a delay, subjects viewed a probe letter and recalled if the probe letter had been one of the encoded letters".

- Capitalize the first letter in all words in the names of conditions and the names of experiments.

- The names of experiments should be taken directly from the coordinate tables in the papers. Some experiment names explicitly state the conditions in them: "Finger Tapping – Rest" or "Finger Tapping vs. Rest" or "Finger Tapping > Rest", and some do not: "Motor Directed Attention" or "Conjunction Analysis". Use the convention of naming adopted in the paper. For conjunction and disjunction analyses, normally all conditions are used in all experiments unless the experiment indicates which conditions were used in each experiment. (See Thierry_HBM-03.ent for an example.)

- Avoid use of the word "task" in the condition (and experiment) name. For example, "Finger Tapping Task" should be named "Finger Tapping".

- When possible, combine "like" instructions for more than one condition in the first sentence of the prose description. Example: "Subjects underwent 5 conditions in which they silently read words immediately after presentation." Then list each condition. When one condition is different from the others, list this condition last.

- If "Rest" is used as a control condition, list it last. The conditions "Rest" and "Fixation" do not require a description.

- Some papers report increases and decreases in activation. Be sure to inspect the coordinate tables carefully, as authors frequently present each in the same table. Increases and decreases should be coded and named as separate experiments. For example, a study may investigate motor function and acquire data on two conditions, Finger Tapping and Rest. The coordinate table may be titled "Increases" and "Decreases" and the paper should be coded with two experiments, not one.

- If the paper includes normal subjects and a patient group, begin by stating this in the first line of the prose description. For example, "Epilepsy patients and normal subjects underwent 3 conditions".

Examples of properly formatted prose descriptions are provided below.

Example 1: Subjects completed a variant of the Posner spatial cueing task. The target was an X or an O and the subjects pressed one of 2 buttons to indicate the target's identity as quickly as possible. Condition 1: Location-Based Valid Cueing: attention was cued by an arrow pointing in the direction of the target positions. Condition 2: Location-Based Invalid Cueing: attention was cued by an arrow pointing in the incorrect direction of the target positions. Condition 3: Object-Based Valid Cueing: attention was cued by a geometric shape drawn to contain the target positions within its boundaries. Condition 4: Object-Based Invalid Cueing: attention was cued by a geometric shape drawn to contain the target positions within its boundaries, and the shape did not contain the target. Experiment 1: Object- > Location-Based Cueing. Experiment 2: Invalid > Valid Cueing.

Example 2: Subjects underwent 4 conditions in which they performed the n-back task. Condition 1: Zero-Back: subjects responded to a single pre-specified target. Condition 2: One-Back: subjects responded if the target was any letter identical to the one immediately preceding it. Condition 3: Two-

Back: subjects responded if the target was any letter identical to the one presented 2 trials back. Condition 4: Three-Back: subjects responded if the target was any letter identical to the one presented 3 trials back. Experiment 1: Load. Experiment 2: Time.

Example 3: Depressed subjects underwent 2 sessions and 1 condition. Session 1: Baseline. Session 2: 8-Week Paroxetine Treatment. Condition 1: Rest. Experiment 1: 8-Week Paroxetine Treatment vs. Baseline.

Example 4: Schizophrenic, bipolar and control patients underwent 4 conditions in which they were presented with auditory and visual stimuli. Condition 1: Verbal Fluency: subjects were presented with a letter aurally and asked to generate a word beginning with that letter internally. Condition 2: Verbal Fluency Control: subjects were cued by the auditory presentation of the word "rest" and asked to internally articulate the word. Condition 3: Semantic Decision: subjects were cued by visual presentation of a noun and asked to decide internally if the word represented a living or non-living object. Condition 4: Semantic Decision Control: subjects fixated on an isoluminant screen. Experiment 1: Verbal Fluency, Bipolar Patients. Experiment 2: Verbal Fluency Control, Bipolar Patients. Experiment 3: Semantic Decision, Bipolar Patients. Experiment 4: Semantic Decision Control, Bipolar Patients. Experiment 5: Bipolar vs. Control, Verbal Fluency. Experiment 6: Bipolar vs. Schizophrenic, Verbal Fluency. Experiment 7: Bipolar vs. Control, Semantic Decision. Experiment 8: Bipolar vs. Schizophrenic, Semantic Decision.

Subjects

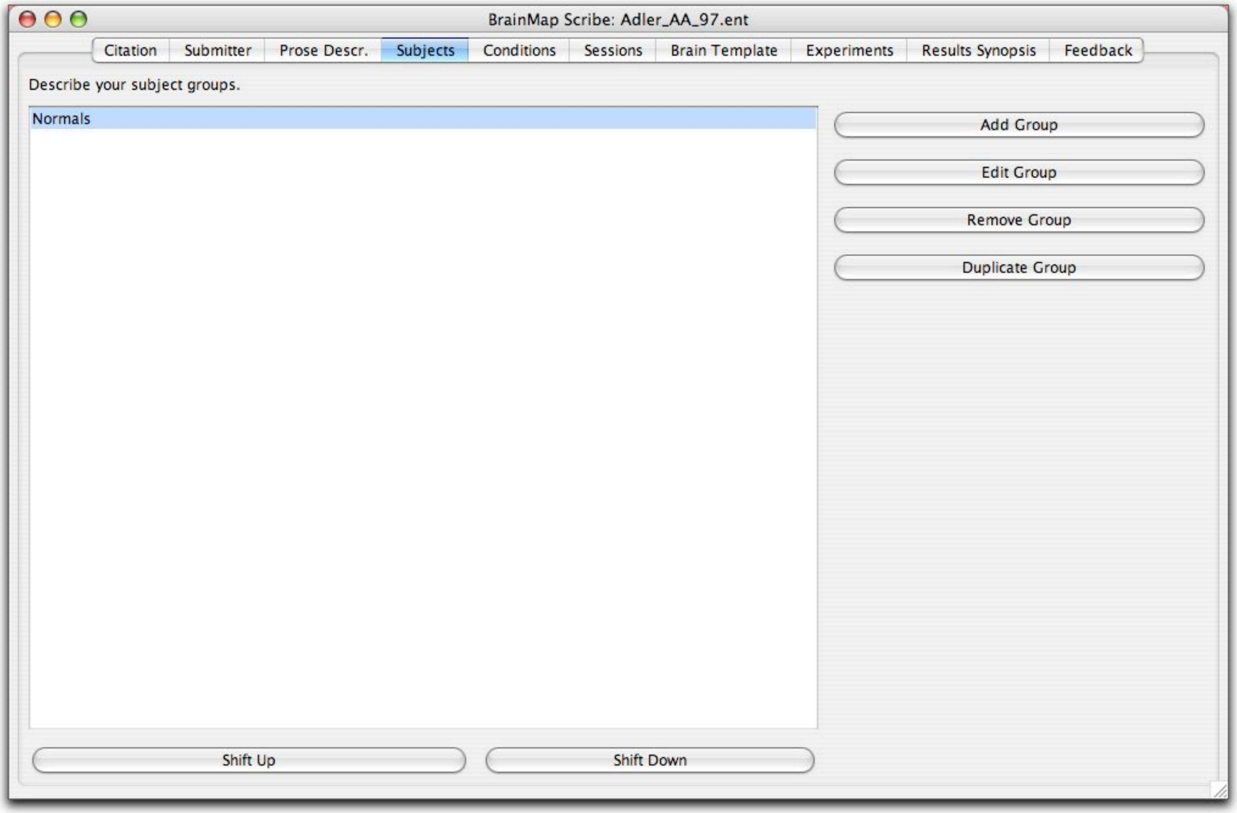

Click the 'Add Group' button to enter subject information in the 'Subjects' panel that appears as a blank window.

To enter several similar subject groups, enter the first set of data, and then click on the group name. Next, click the 'Duplicate Group' button. Data entered for the first group will be copied into a new subject group window. Rename the duplicate subject group and change the data as appropriate.

If coordinates reported are for individual subjects, as opposed to group mean data, enter each subject's information as an individual group.

Subjects: Subject Group

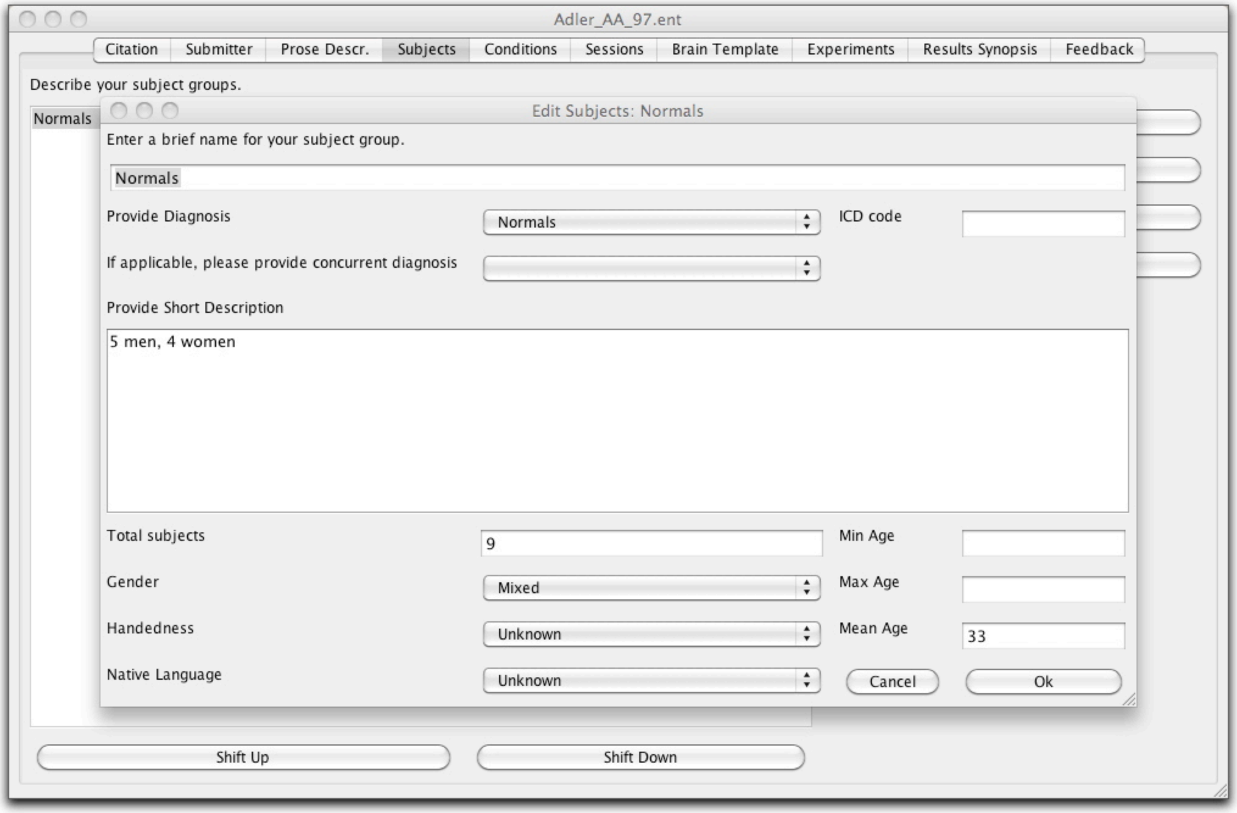

<u>Name of Subject Group</u>
Provide a brief name for the subject group, for example, “Normals”.

<u>Provide Diagnosis</u>
Select from the list any medical or psychological conditions.

<u>Concurrent Diagnosis</u>
If applicable, please select a concurrent diagnosis from the list. Generally, this is left blank.

<u>ICD Code</u>
Most diseases are listed in the International Classification of Diseases (ICD). If the ICD number is not known (usually the case), then leave this blank.

<u>Provide Short Description</u>
Enter the ratio of male to female subjects (e.g. 4 women, 5 men). Also, enter the ratio of right-handed and left-handed subjects (e.g., 6 right-handed, 4 left-handed).

<u>Total Subjects</u>
Enter a number into the space provided. Only integers are allowed.

<u>Gender</u>

Select the gender from the pull-down menu. If “Mixed” gender is selected, indicate the number of male and female subjects in the short description.

Handedness

Choose one option from the menu to describe the handedness of the subject group. Use the ‘Mixed’ option if the subjects had different handedness.

Native Language

Choose the native language or languages of the subjects, or add a new language to the list by choosing ‘Other’ and typing into the window that appears.

Ages of Subjects

Fill in ‘Min Age,’ ‘Max Age,’ and ‘Mean Age’, if reported. Only integers are allowed.

Conditions

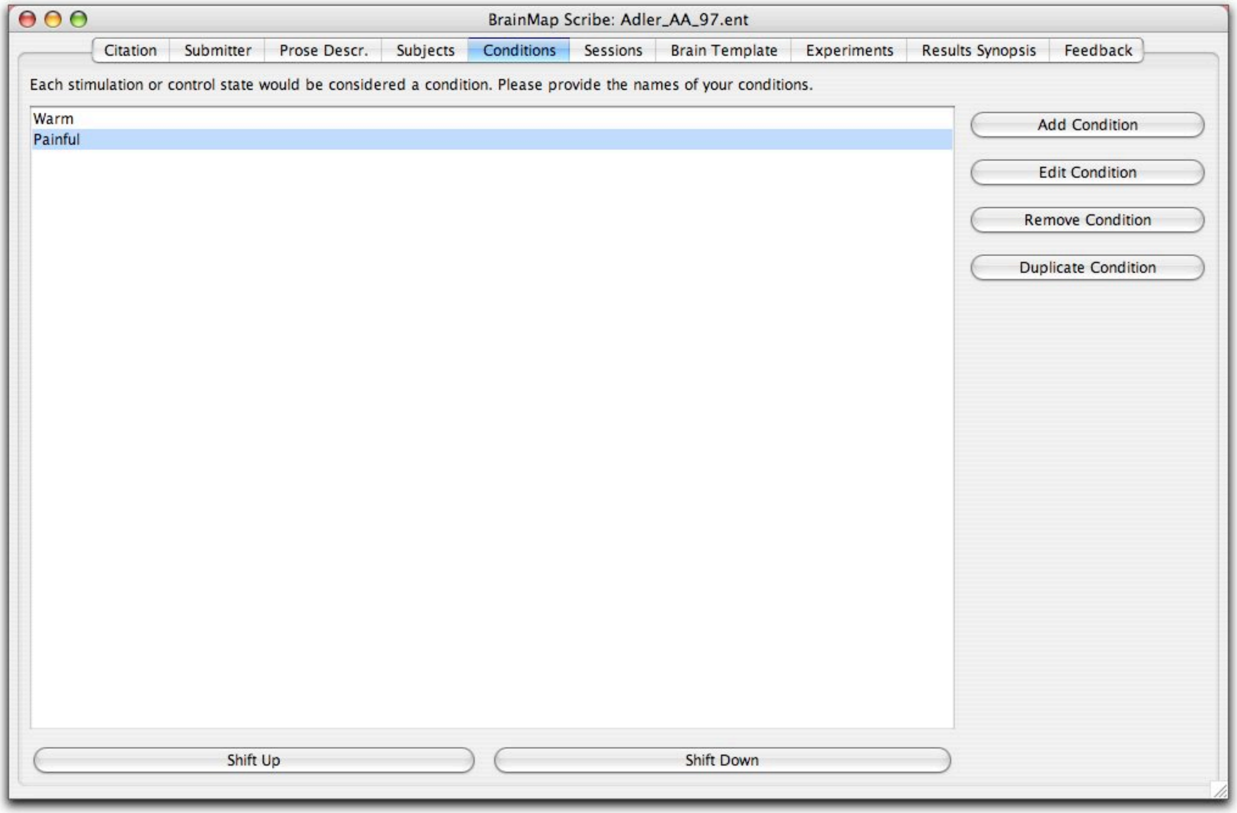

Enter all conditions used in the paper, including the control conditions, e.g., Rest or Baseline.

To change the order listed to better reflect the order used in the experiment, click the ‘Shift Up’ or ‘Shift Down’ button at the bottom of the window.

Conditions: Stimulus, Response, and Instructions

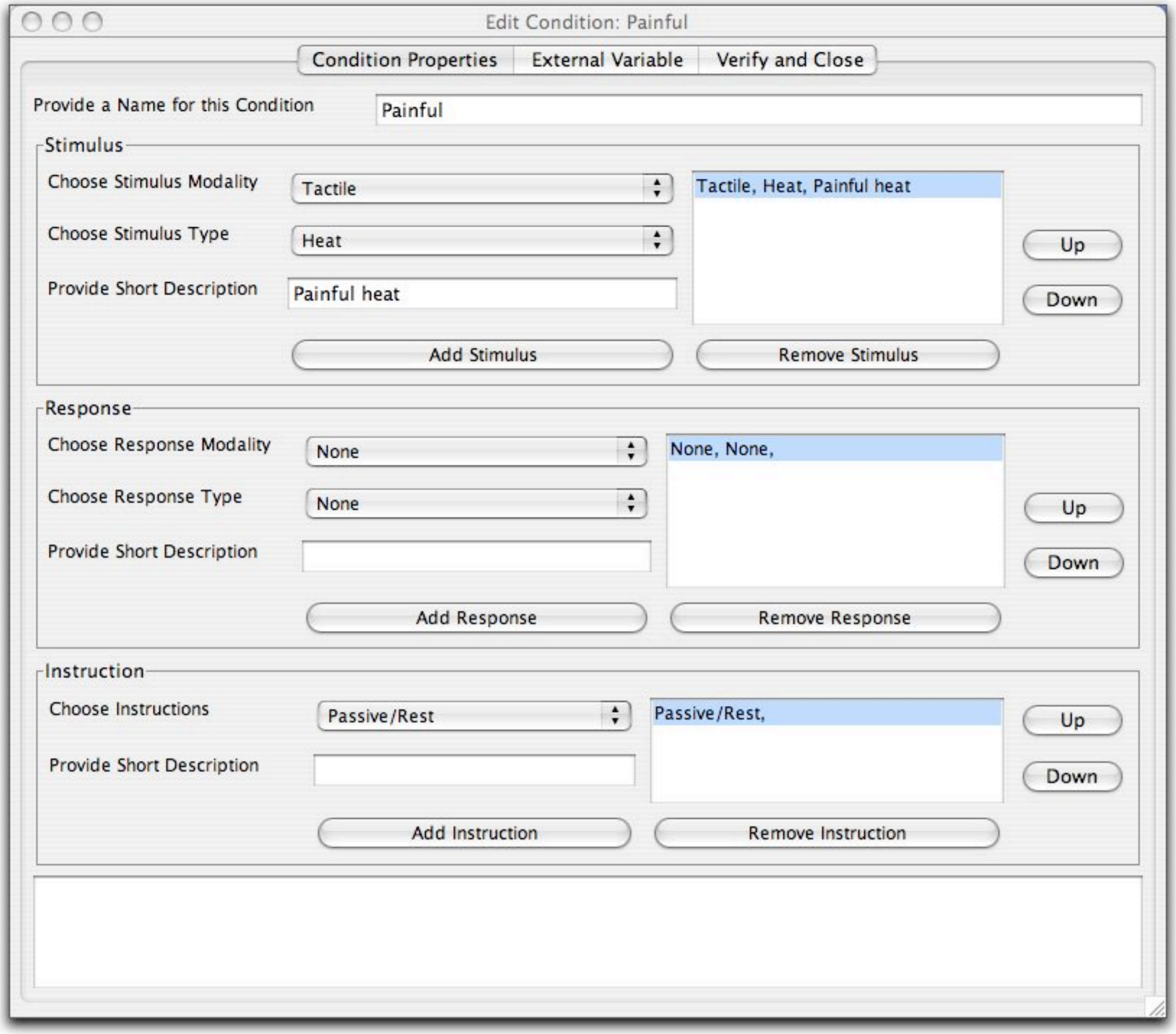

<u>Provide a Name for this Condition</u>
Use the same naming system as defined in the prose description. Again, try to keep the naming convention of the paper, but use your best judgment. Sometimes authors can be confusing and will invent a very long condition name for a simple task. If, in the interest of brevity, it is necessary to formulate your own condition names, then do so.

<u>Choose a Stimulus Modality</u>
Choose the sensory mechanism through which the subject was stimulated. That is, what was used to stimulate one of their five senses while they were in the scanner?

<u>Choose Stimulus Type</u>
Select the specific stimulus from the drop-down menu. If these choices do not match the stimulus exactly, choose 'Other' then click the ‘Add’ button.

Provide Short Description
Fill in any additional details about the stimulus that are necessary for a firm understanding of the paper or that add interest to the study. This might include information about the exact type of stimulus not apparent from the 'Stimulus Type' selected, for example, "Letter Strings" or "Spanish Nouns".

Choose Overt Response Modality
Enter measurable responses only, for example, imagined movement and silent verb generation are covert responses and should not be entered. If the response was a button press, choose 'Hand' for the modality.

Choose Overt Response Type
Select the appropriate response type. Use the Overt Response Type 'Ocular' for saccades or eye movement only, not for simply looking at something. Don't forget to click on "Add".

Provide Short Description
Fill in any additional details about the response that are truly necessary for a firm understanding of the study.

Choose Instructions
Choose the instruction that best describes those used in the study. If a subject was instructed to read words aloud, choose 'Read' as the instruction and 'Speech' as the subject's response; rather than choosing 'Speak' as the instruction. If the instructions were to attend and then press a button upon perceiving a target, it is only necessary that you enter the instruction to 'Attend'. 'Button Press' should be entered only as a response. Don't forget to click on "Add".

Please use a complete sentence to describe the instructions (e.g., Subjects were instructed to generate verbs in response to the presented nouns). Also, please use the past tense.

- Do not confuse "detect" and "discriminate": Detect is to discover the presence of, i.e., does target exist?, while discriminate is to distinguish from another "like" object by discerning differences, i.e., choosing a target.
- Do not use "attend" every time the subjects pay attention to the stimulus, otherwise it will be coded in every paper. Only use "attend" in studies where the subjects' only instruction is to pay attention to the stimuli.

Provide Short Description
Any additional details about instructions that are necessary for a firm understanding of the study should be entered in this field.

Short Description – Bottom Window
Use this space to enter any summary or clarifying information that pertains to the condition as a whole, i.e., that cannot be broken into one of the three components (stimulus, response, instructions). If the description CAN be classified as one of the three components, then enter the desired information into one of the short description fields listed above.

Also, making a mental note of the differences in various conditions of your paper during entry will assist in identifying the contrast when entering experiment information later on.

Before exiting this panel, make sure that the way this condition differs from the other condition(s) in your paper can be determined by looking at the Conditions panel (i.e., don't duplicate conditions).

To edit existing conditions, simply double-click on the selected field entry. This will populate the condition information back into the drop-down menus so that you may edit the details.

If necessary, use the 'Up/Down' buttons to organize the condition information to reference time-dependent stimuli, responses, or instructions (stimuli presented first listed first, while stimuli presented later listed second).

Conditions: External Variable

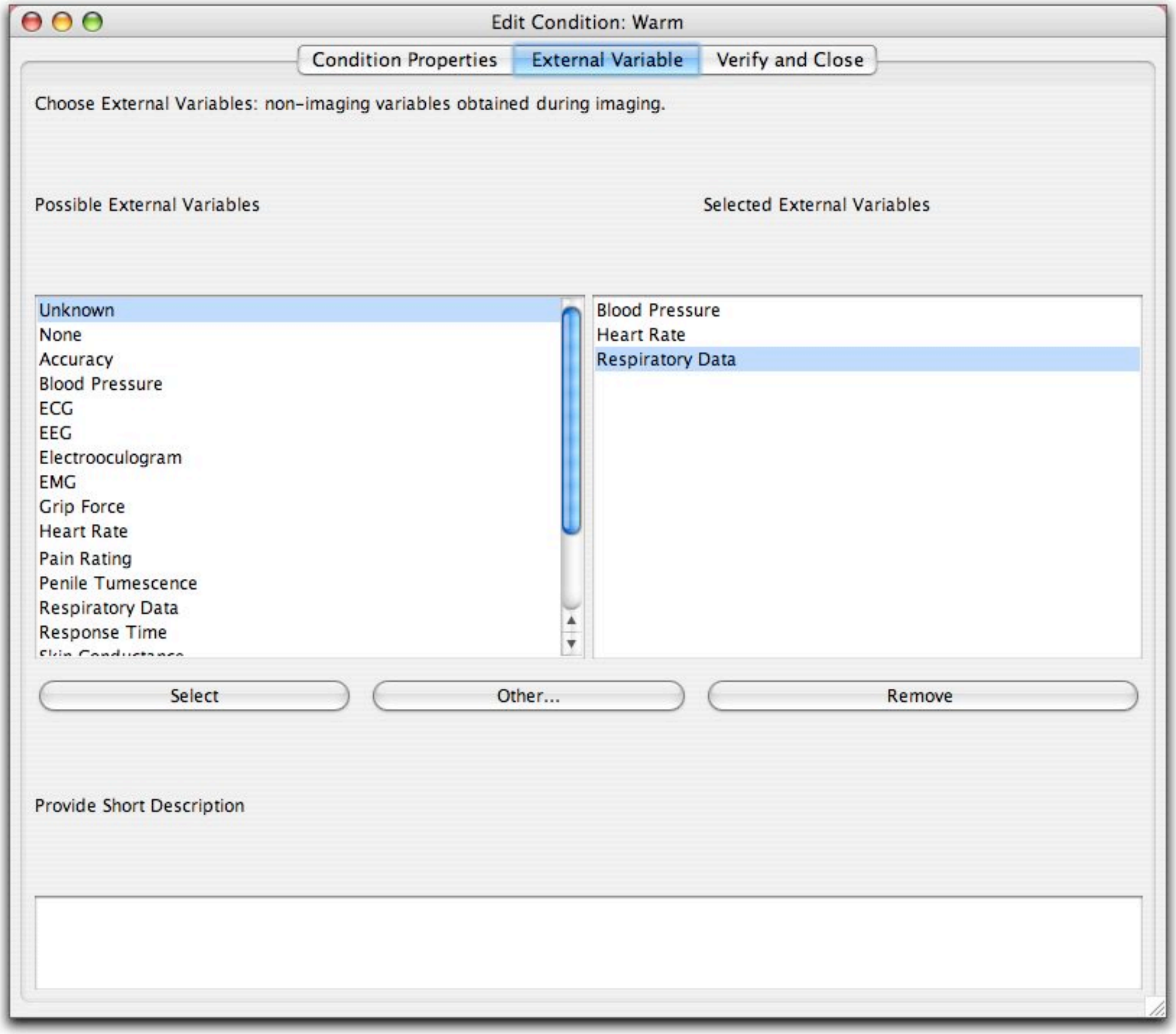

This menu is primarily intended for studies wherein an external variable was correlated with brain activity. If a correlation included an external variable, enter the variable here. After selecting the desired option from the menu, click the 'Select' button.

The external variable is any measurement taken during the scan, e.g., eye movement, heart rate, accuracy, temperature, etc. In order to be listed here, the external variable must be specifically stated in the study. When there is no external variable, select "None".

Many studies record accuracy and response time. Do not forget to include these external variables.

Conditions: Verify and Close

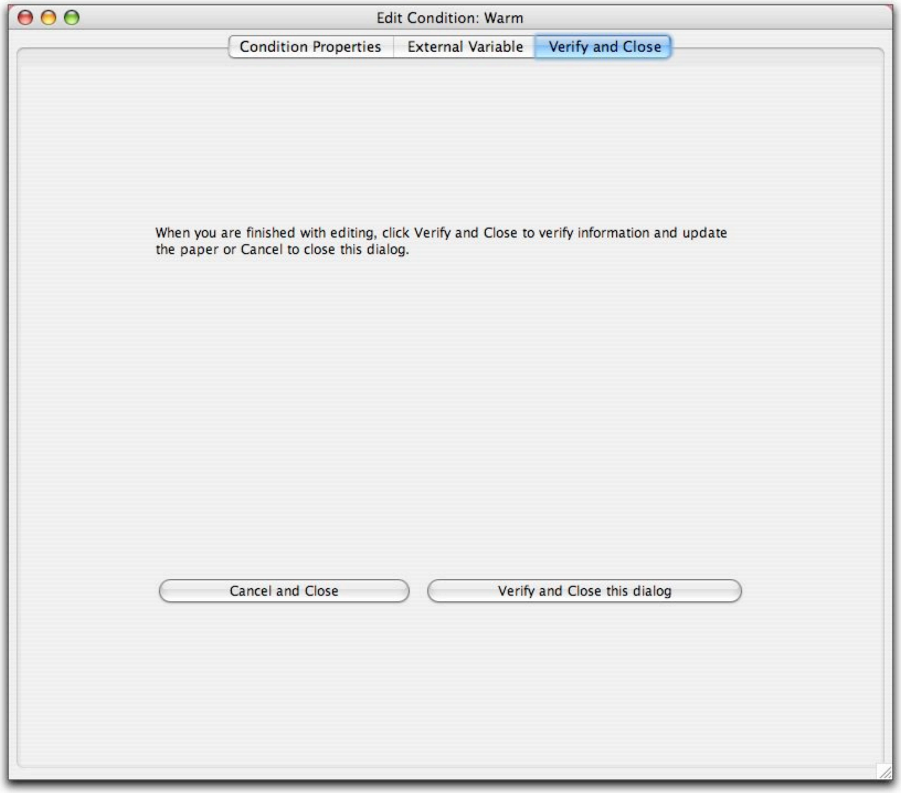

Once all condition information has been entered, click on the ‘Verify and Close this dialog’ button. To discard the changes, select 'Cancel and Close'.

Sessions

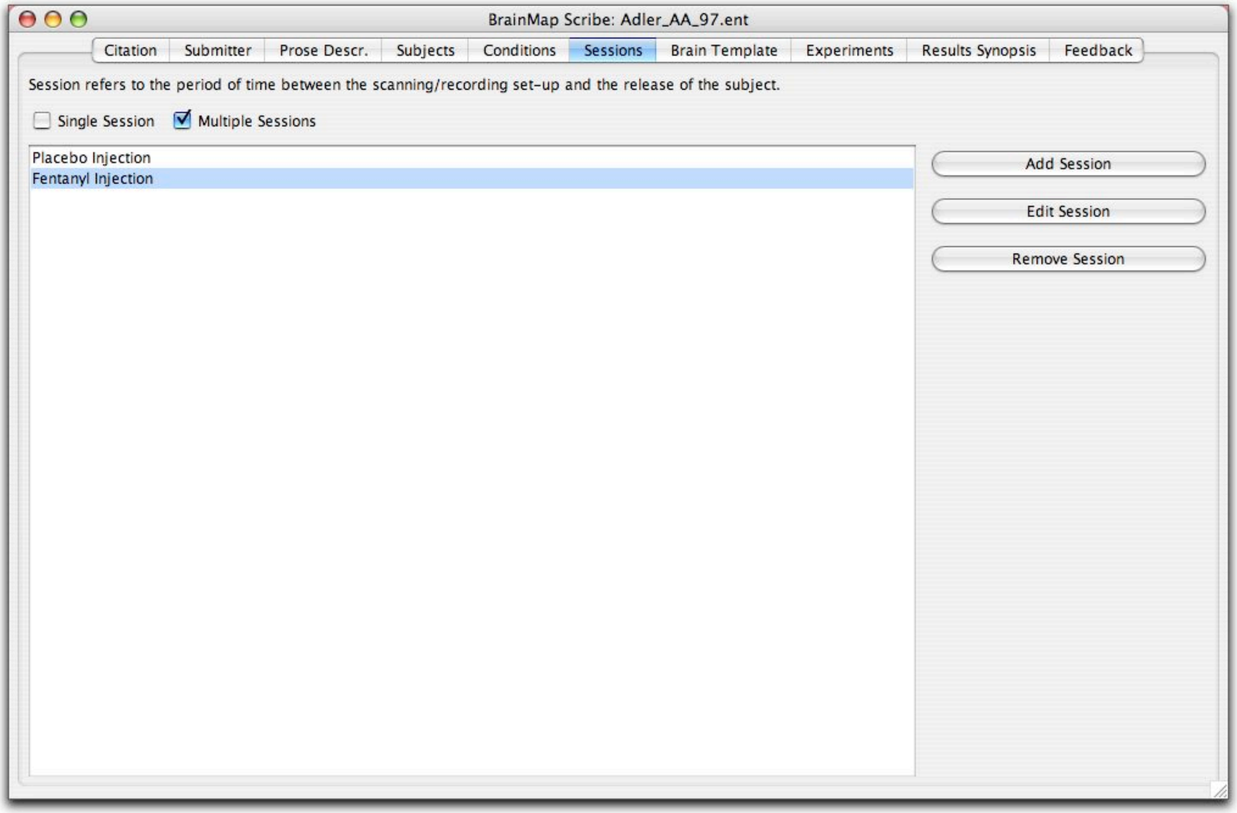

'Sessions' refers to the period of time between the scanning/recording set-up and the release of a subject. **Most studies occur during only one session**. If a subject is set up, released and then set up again, the second set-up marks the beginning of a second session. Before-and-after-treatment studies and before-and-after-practice studies would involve multiple sessions.

- For one-session studies, no entries are required on this panel.
- For multiple sessions, check on 'Multiple Sessions' and click the 'Add Session' button. A message box will then appear prompting you to enter at least two sessions.
- If your paper includes multiple sessions, be sure to include this design in the prose description.

Sessions: Session Name

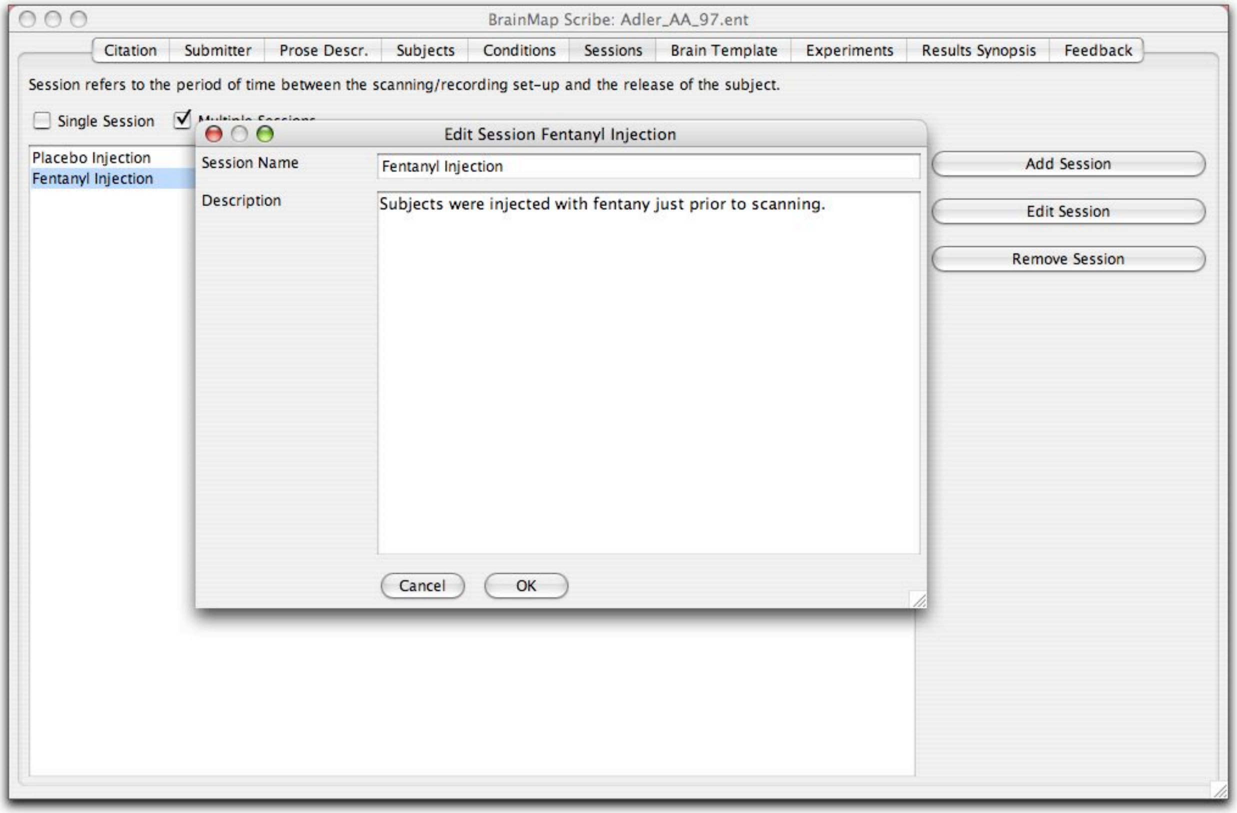

Give the session a brief name, for example "Before Treatment" or "After Therapy".

<u>Description of this Session:</u>
Describe the session, including sufficient detail to distinguish this session from other sessions and to give a firm understanding of the study. Please use a complete sentence to describe the session.

Brain Template

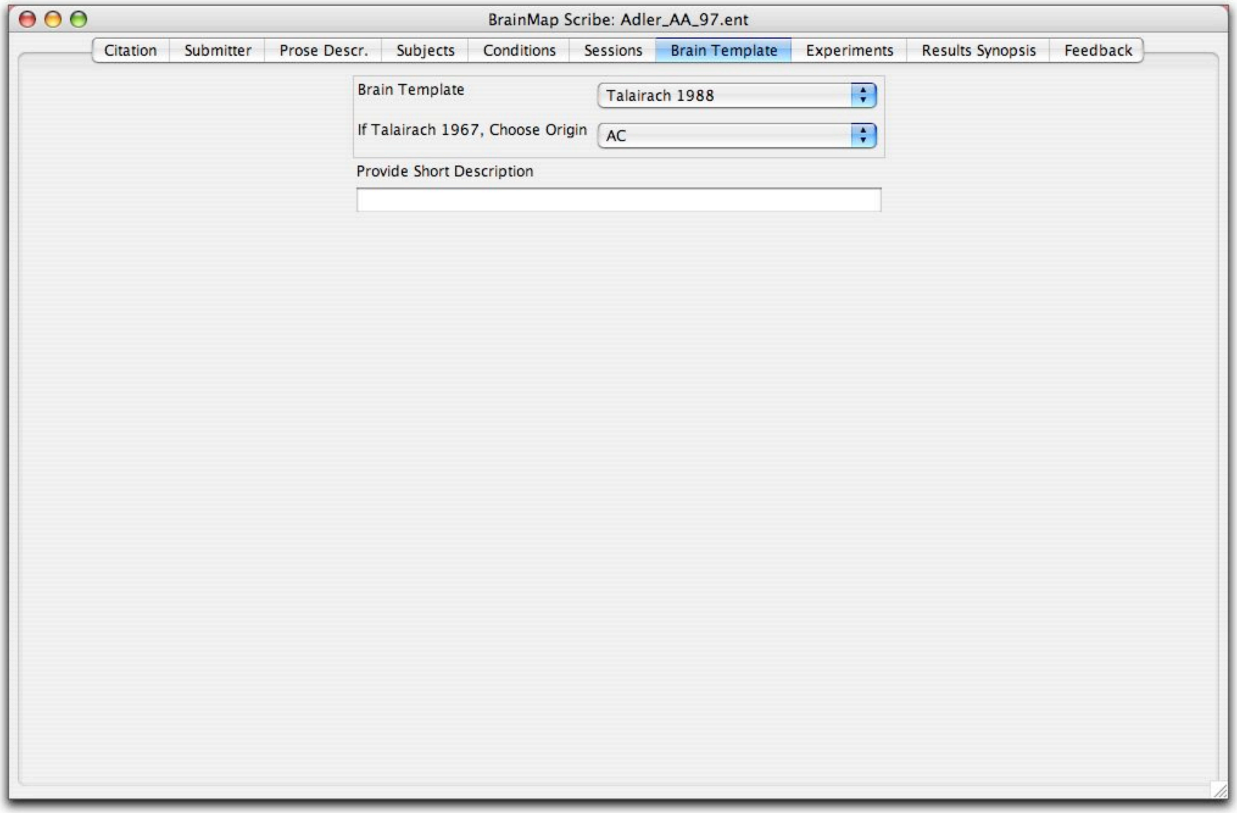

Click on the drop-down menu provided and choose the brain template used.

<u>Short Description</u>
Enter the software used for spatial normalization (e.g., "SPM96", "FSL", "SPM99, then converted to Talairach space using the Brett transform", etc.).

All MNI coordinates will be automatically converted to Talairach space using the icbm2tal transform (Lancaster et al., 2007). All MNI coordinates converted to Talairach space via the Brett transform **in the original publication** will be subject to 2 transforms: (1) reverse-Brett to convert back to MNI space and (2) icbm2tal for correct transformation from MNI to Talairach space.

Experiments

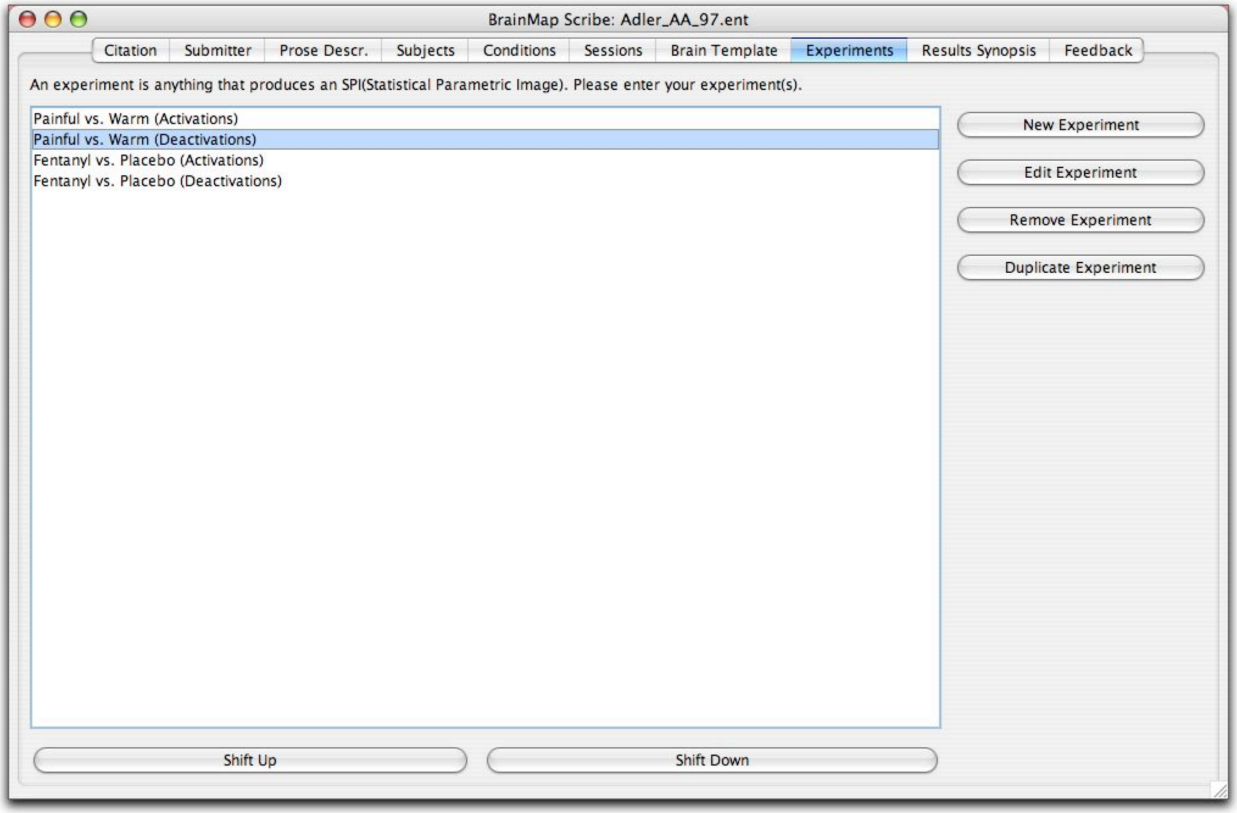

Enter experiments according to the names and order entered in the 'Prose' panel.

To enter several similar experiments, enter the information for the first experiment and verify and close it. Then click on its name and click the 'Duplicate Experiment' button. The experiment data will be copied into a new experiment. Enter a new name for the new experiment, making changes to the existing data as needed.

Shortcut: Many experiments are very similar within a paper, so it can save time if you enter in all the information for the first experiment EXCEPT for the coordinates. Then save the experiment, duplicate it, and edit the duplication(s) for the additional experiment(s). The final step is to go back and enter in the coordinates for all the experiments once they have been created.

Experiments: Context

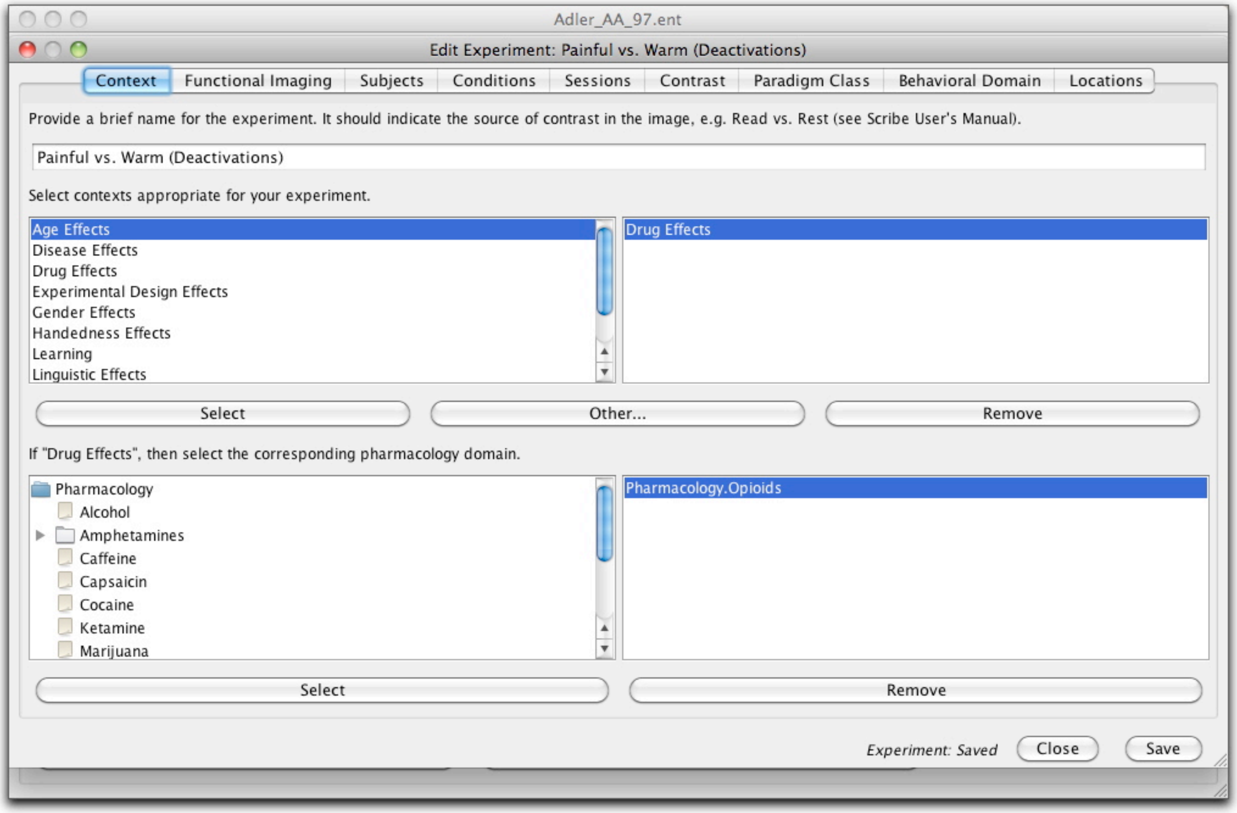

<u>Provide a brief name for the experiment</u>
The names entered here must be the same as those used in the Prose Panel.

<u>Select contexts appropriate for the experiment</u>
'Context' refers to the broadest category to which an experiment belongs within the realm of brain imaging. Most frequently, the context of "Normal Mapping" will be selected.

"Experimental Design Effects" is ONLY selected for studies in which a technical aspect of the paradigm is being manipulated, such as when comparing the effect of the rate of presentation of the stimuli ("Self-Paced n-back vs. Fixed-Paced n-back" or "Slow Words vs. Fast Words". This is NOT the appropriate context when simply contrasting two or more conditions – that is "Normal Mapping".

<u>Pharmacology</u>
If "Drug Effects" is selected as a context, then you will be prompted to enter a corresponding pharmacological domain from the available list of options.

Experiments: Functional Imaging

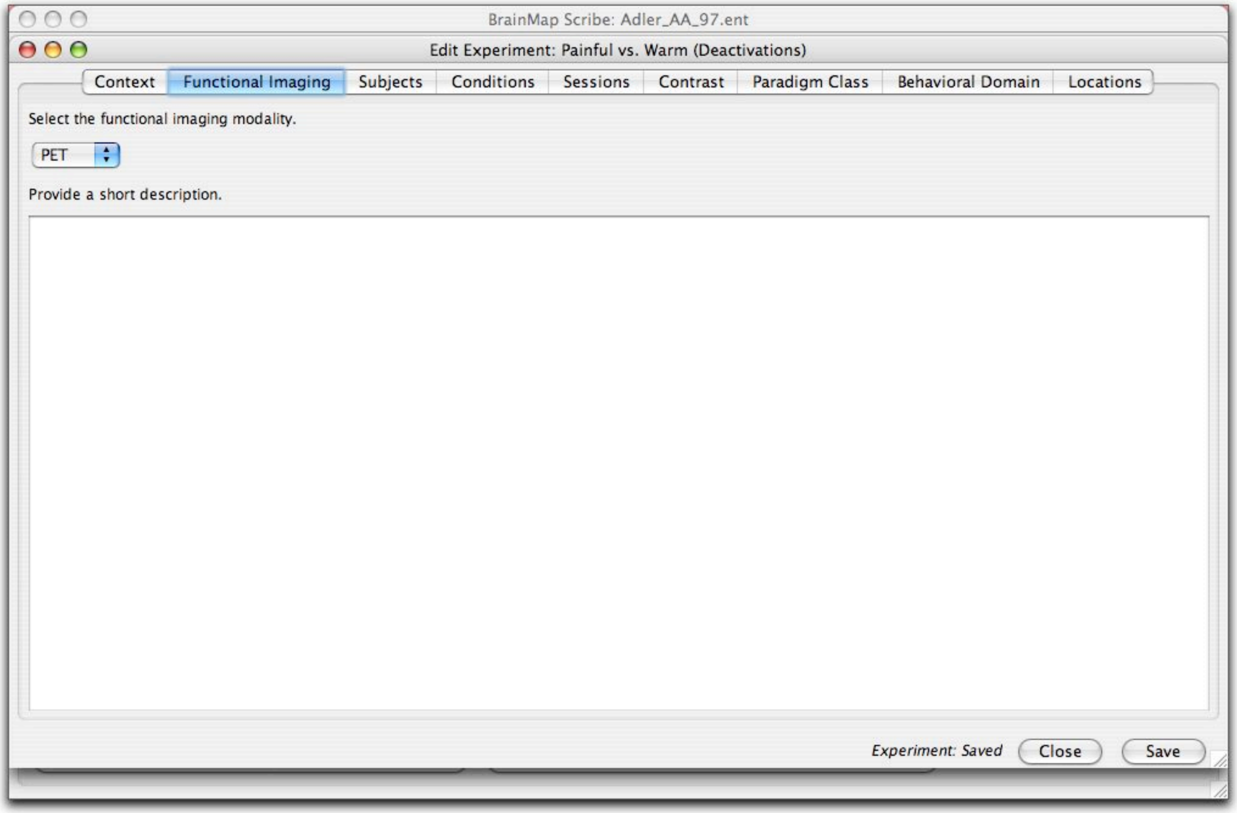

Functional Imaging Modality
Select the functional modality from the pull-down menu.

Short Description
If the imaging modality if MRI, please provide the magnet strength in the short description, if available (e.g., 3T).

Experiments: Subjects

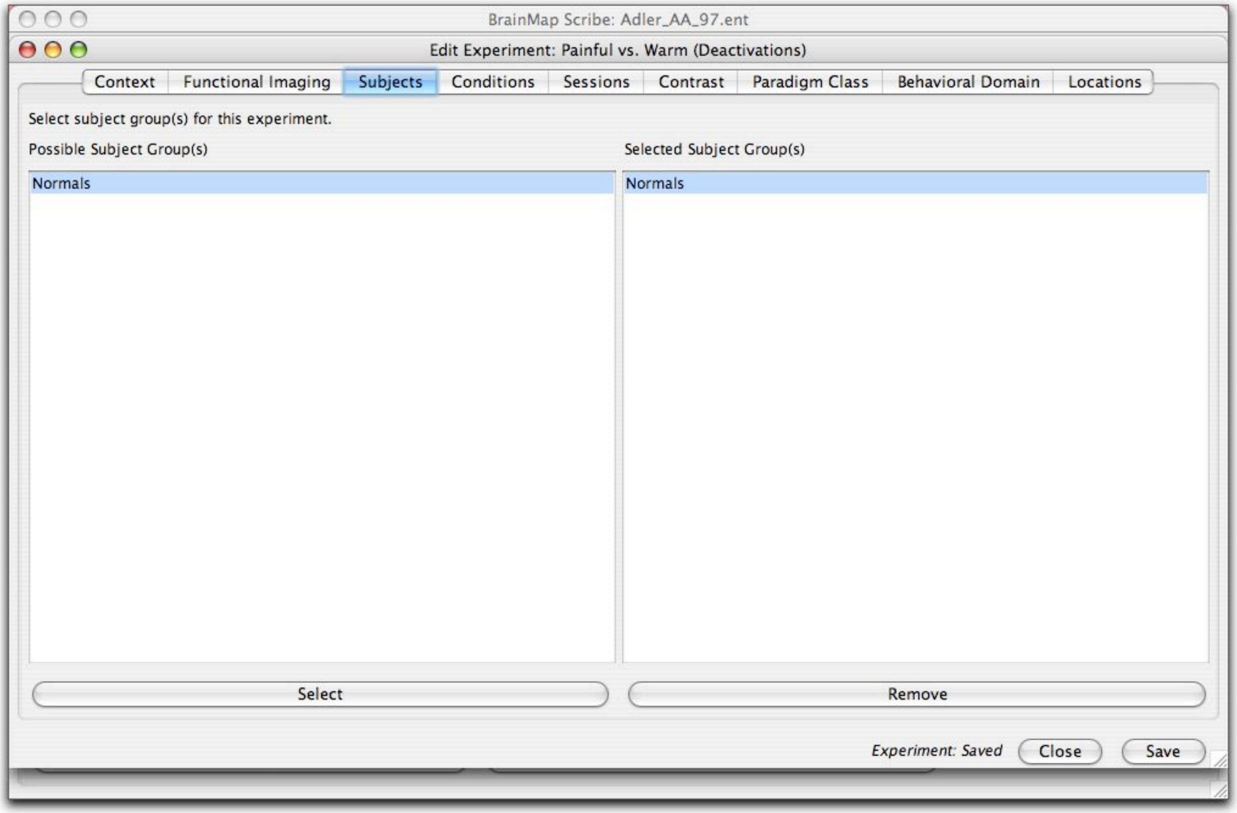

This panel contains the list of groups entered on the previous 'Subjects' panel. Click on the group used for the experiment you are presently entering and then click 'Select'.

Experiments: Conditions

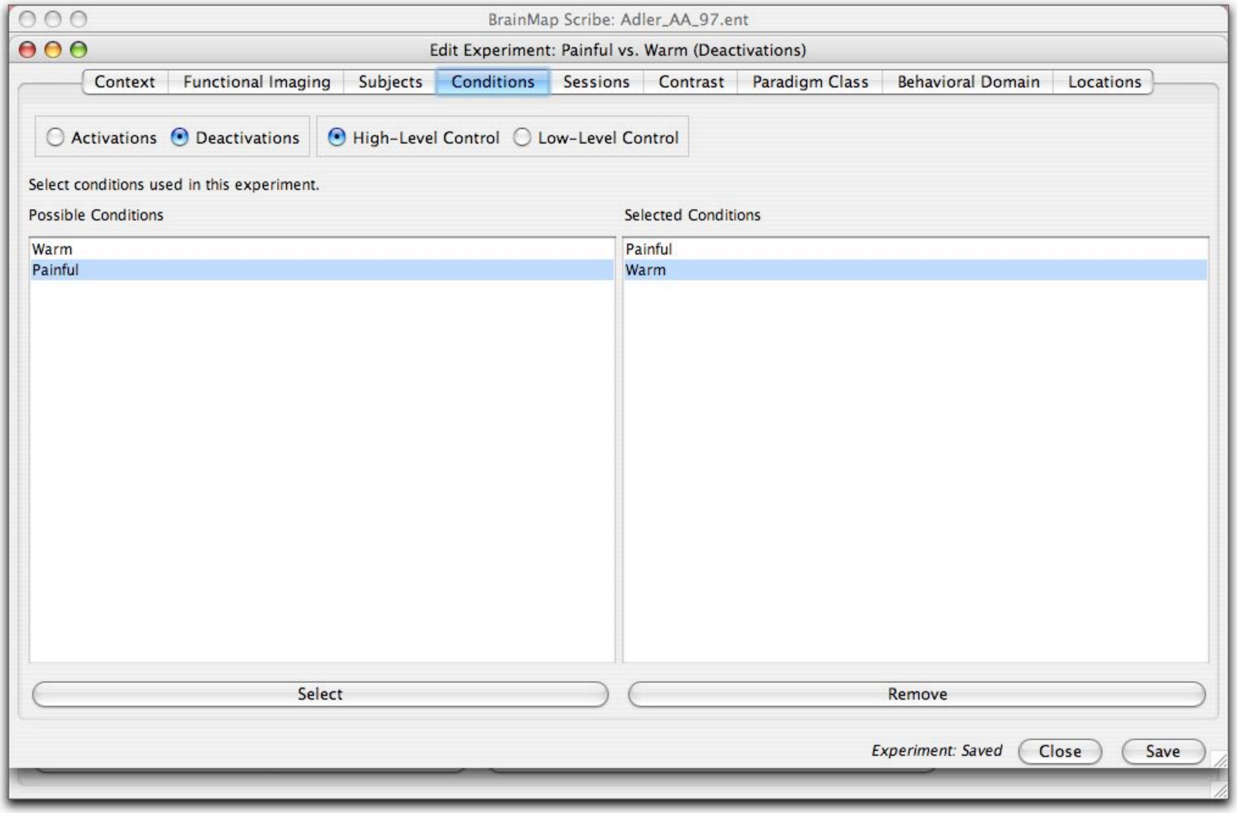

Check the box to indicate whether this experiment reports activations (increases) or deactivations (decreases) and whether a high- or low-level of control was used to contrast with the activation condition. **Only select 'Low-Level Control' for rest or fixation control conditions**.

Select Conditions

This panel contains the names of the conditions you provided previously on the 'Conditions' panel. Choose the conditions included in this experiment. After selecting a condition from the menu, click the 'Select' button.

Experiments: Sessions

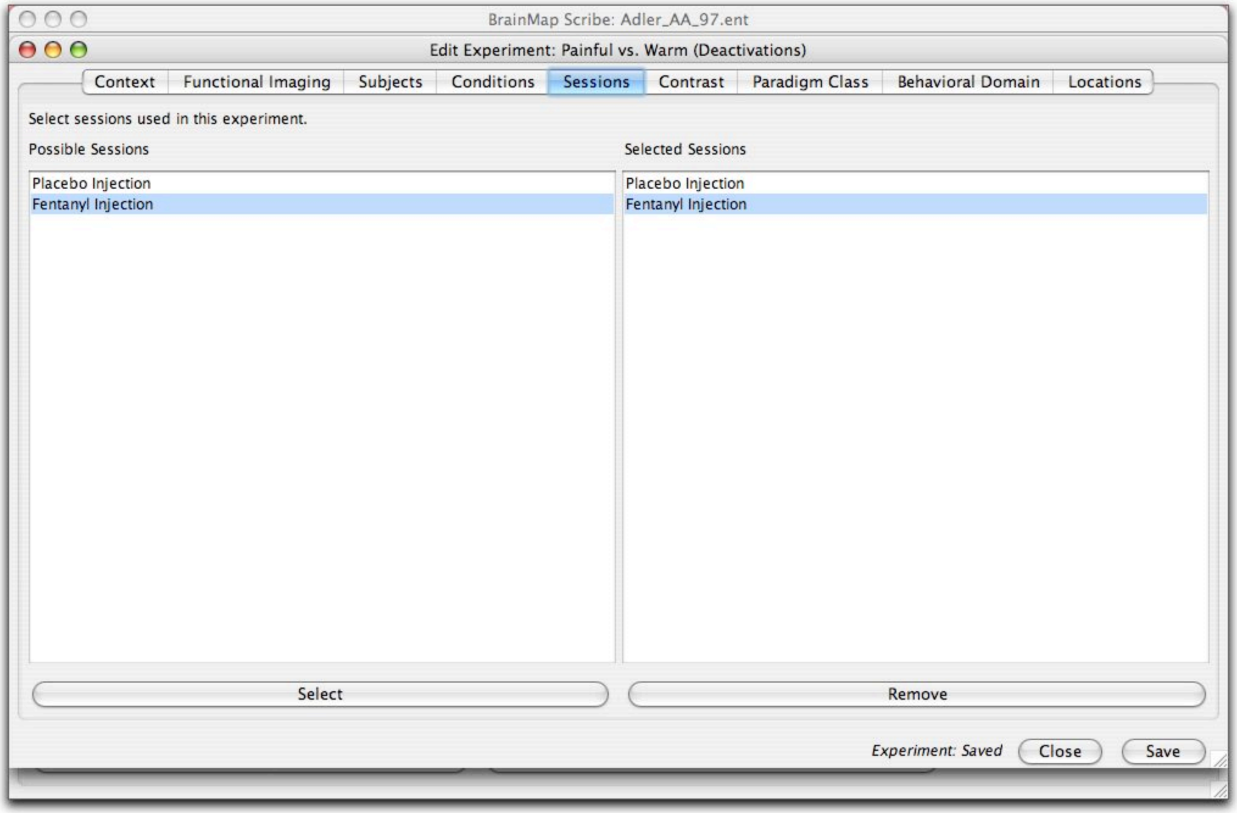

<u>Select Sessions</u>
This panel contains the names of the sessions previously provided on the 'Sessions' panel. If the paper included more than one session, choose the session used in this experiment. If not, ignore this panel.

Experiments: Contrast

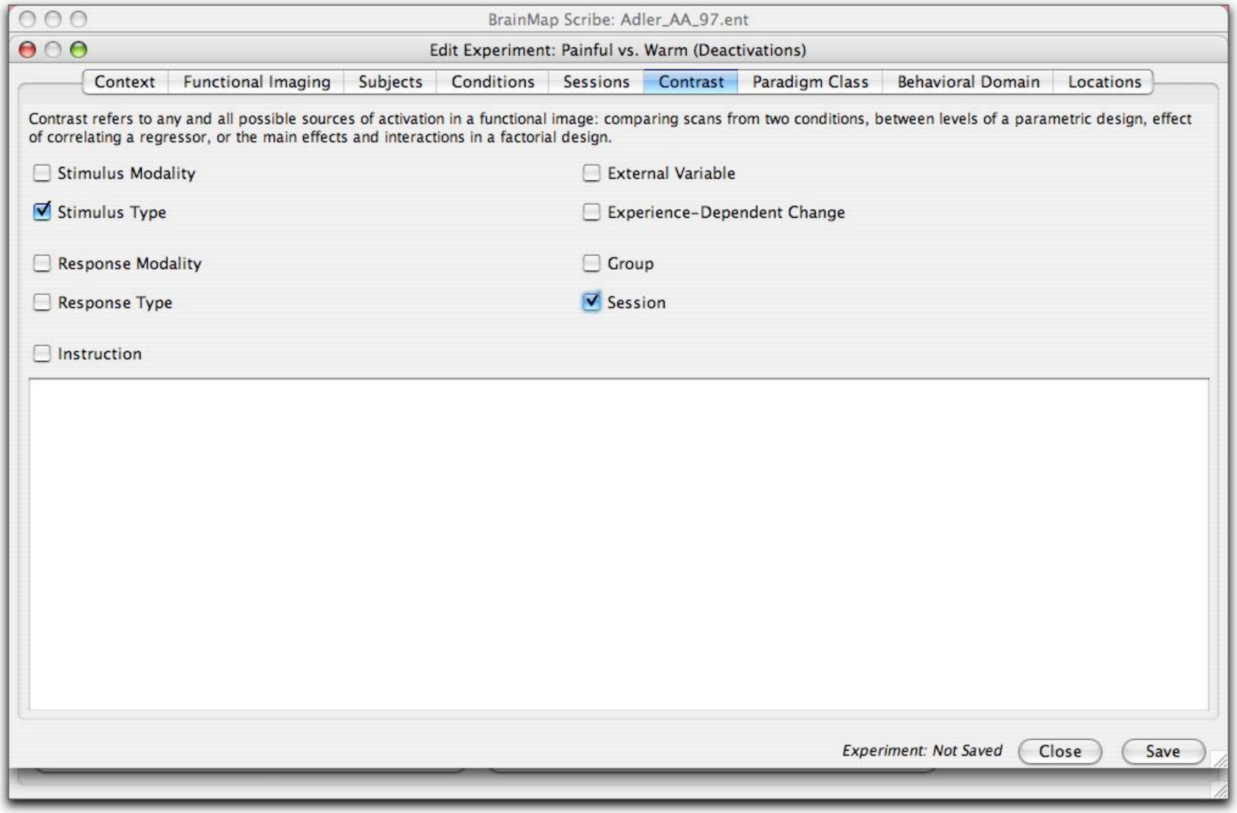

<u>Contrast</u>
The purpose of contrast is to identify the key aspect of the study. In a given experiment, the contrast fields indicates how the conditions being compared in an experiment are different.

To choose the source of contrast in the experiment, click on the box to the left of the description. Make as many choices as appropriate for each experiment. To help identify the contrast, refer to the Conditions panel and look at the differences in condition stimulus, response and instructions.

<u>Provide a short description</u>
Use the blank window in the lower half of this panel to summarize and clarify the contrast of a given experiment. If "External Variable" is selected, please provide the corresponding external variable in this window.

Example 1: Upon entering descriptions for the stimulus, instruction, and response used in each condition in an experiment, you notice that even though the stimulus used in Condition 1 was different from the stimulus used in Condition 2, the given choices for describing the instruction do not indicate that there is indeed any difference. For instance, an experiment might involve the following: Condition 1: the subject focused on a fixation point while a checkerboard wedge stimulated one part of the visual field. There was no response. Condition 2: the subject focused on a fixation point while a checkerboard wedge stimulated a more peripheral part of the visual field. There was no response. In entering the descriptions for these conditions, you notice that both are described as Stimulus Modality: Visual; Stimulus Type: Checkerboard; Response Modality: None; Response Type: None; Instruction: Attend. The

point of interest of this experiment is not indicated by the condition descriptions. For the Contrast field, however, you have the opportunity to indicate which aspects of the conditions differed. In this experiment, the choice of contrast selected would be Stimulus Type because it was the stimulus that differed between conditions. All other fields would remain unchecked.

Example 2: If the experiment involved Condition 1: subject viewed nouns on a screen and was asked to generate verbs aloud which pertain to the nouns on the screen, and Condition 2: subject viewed nouns on a screen and was asked to read the words aloud, the only difference here is in the instructions. For both conditions, the stimulus is visual words, and the response is speech. In this case, the instructions for Condition 1 would be ‘Generate’ and the instructions for Condition 2 would be ‘Read'. It is implied that during Condition 1 the subject had to read the nouns first in order to generate verbs. Therefore, the contrast between the conditions is only in 'Instructions'. All other fields would remain unchecked.

Example 3: If the experiment involved Condition 1: subject listened to nouns presented aurally one at a time with instructions to generate verbs which pertain to the nouns, and Condition 2: subject listened to nouns presented one at a time and was instructed to generate words that rhyme with the nouns, then in this case, all three fields (stimulus, response, instructions) would be entered identically for the two conditions. However, the generation performed in each condition was indeed different. Therefore, enter again the contrast as 'Instructions'. All other fields would remain unchecked.

Example 4: Group Contrast: If the conditions of an experiment are identical, but were endured by two different groups, this would be considered a Group contrast. In this case, click the box to the left of ‘Group’ and all other boxes would remain unchecked.

Example 5: Session Contrast: If all of the conditions of an experiment were identical, and were endured by only one group, but during more than one session, this would be considered a Session contrast. Select the ‘Session’ box and all other boxes would remain unchecked.

Example 6: External Variable Contrast: If an experiment included an external variable with which to conduct a correlation with brain activity, and this was the means for cultivating brain images, choose the box for ‘External Variable’. This contrast type must be selected for all experiments that investigated the accuracy of the responses.

Example 7: Experience-Dependent Change: If the experimental results could have been affected by experience, for example, learning, order, priming, repetition, or training, select the box for 'Experience-Dependent Change'.

Experiments: Paradigm Class

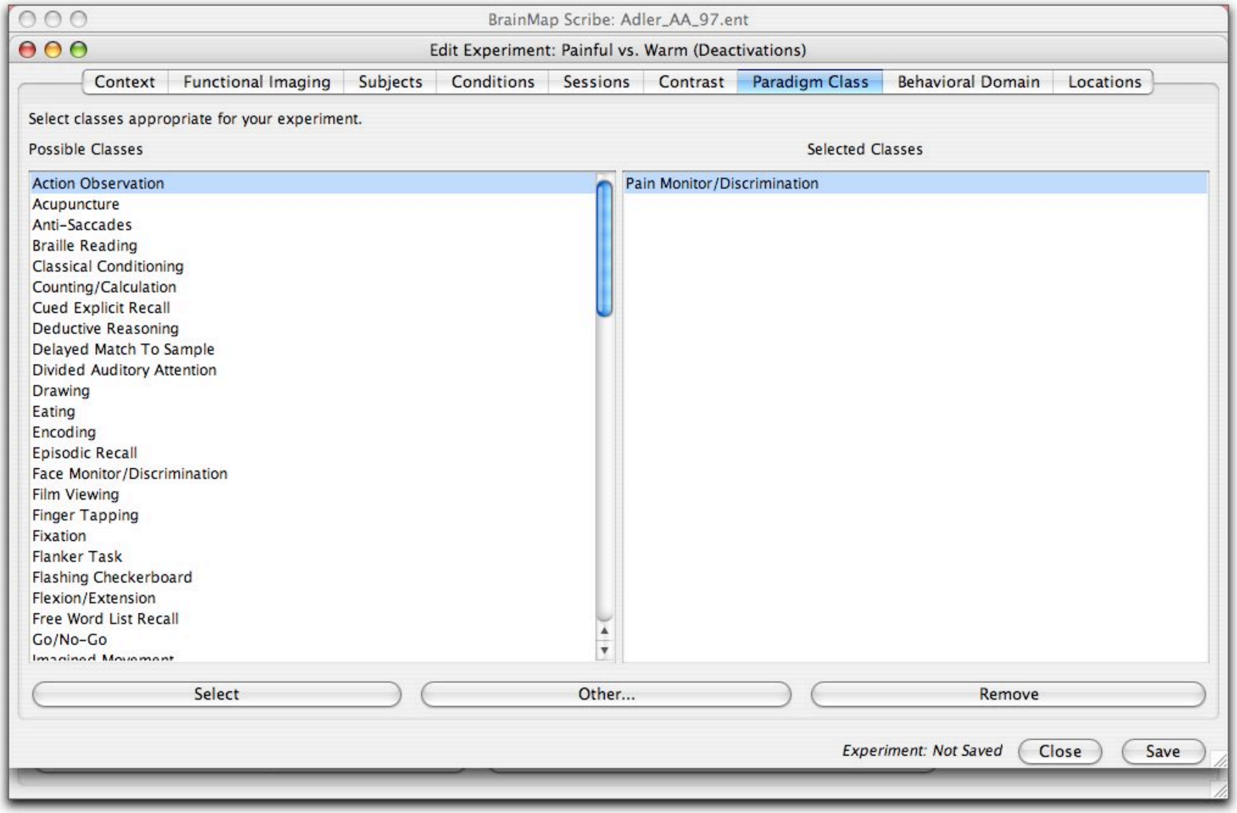

<u>Paradigm Class</u>

Paradigm Class refers to those experimental paradigms that have been used repeatedly by different researchers. Some have become widely known and accepted by brain imagers and have acquired informal (or formal) names. This list is not intended to include a fitting description of every experimental paradigm imaginable, but evolves with the field.

Select only the Paradigm Class that fits the Activation condition. For example, if the Activation condition is the Stroop task, and the Control condition is Fixation, then for Activation – Control, select Stroop only, not Stroop AND Fixation.

Experiments: Behavioral Domain

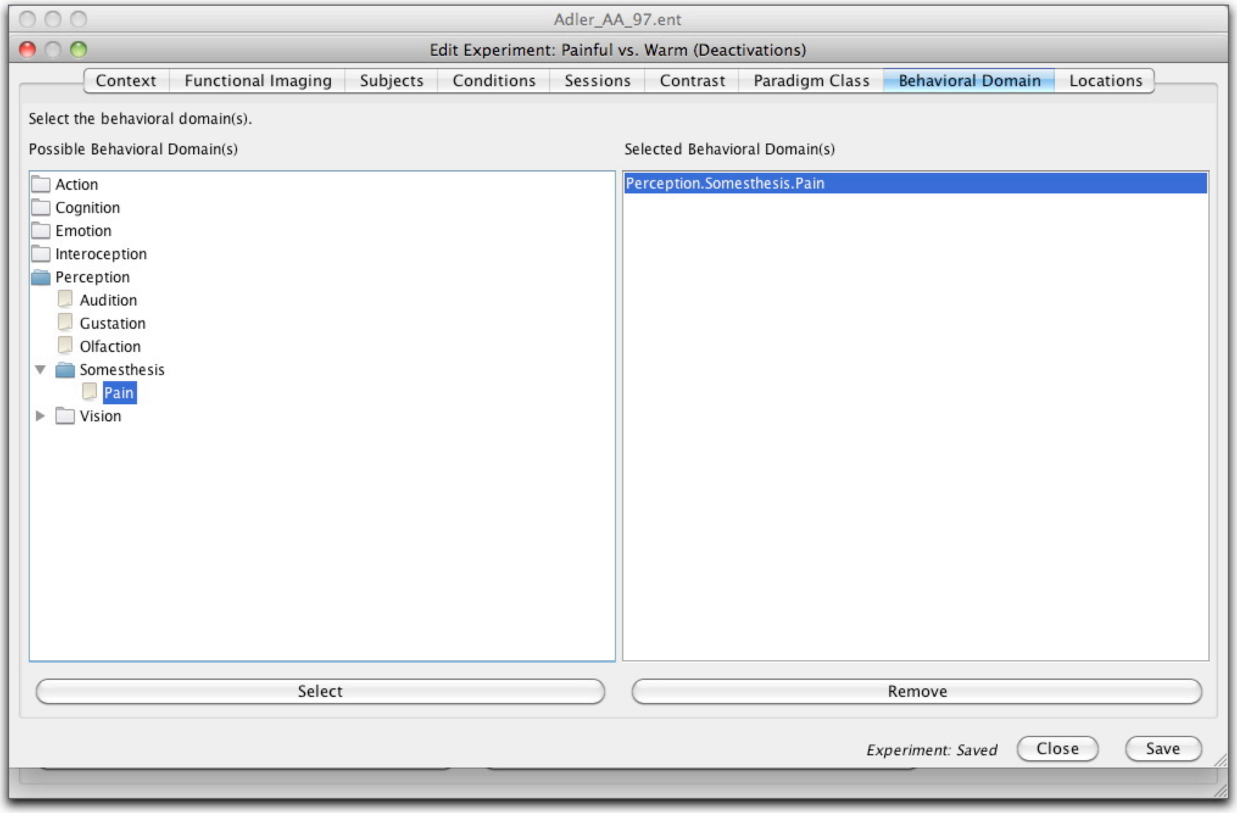

<u>Behavioral Domain</u>
The categories on the left provide several subcategories as drop-down menus. Double-click on the categories to see the related subcategories. These categories and subcategories classify the mental operations likely to be **isolated by the experimental contrast**.

Select only the Behavioral Domain that fits the Activation condition. For example, if the Activation condition is semantic in nature, and the Control condition is phonological in nature, then for Activation – Control, select Semantics only, not Semantics AND Phonology.

- Use both Behavioral Domains of Cognition.Language.Speech and Action.Execution.Speech with overt speech production, but with covert speech production use only the Behavioral Domain of Cognition.Language.Speech.

Experiments: Locations

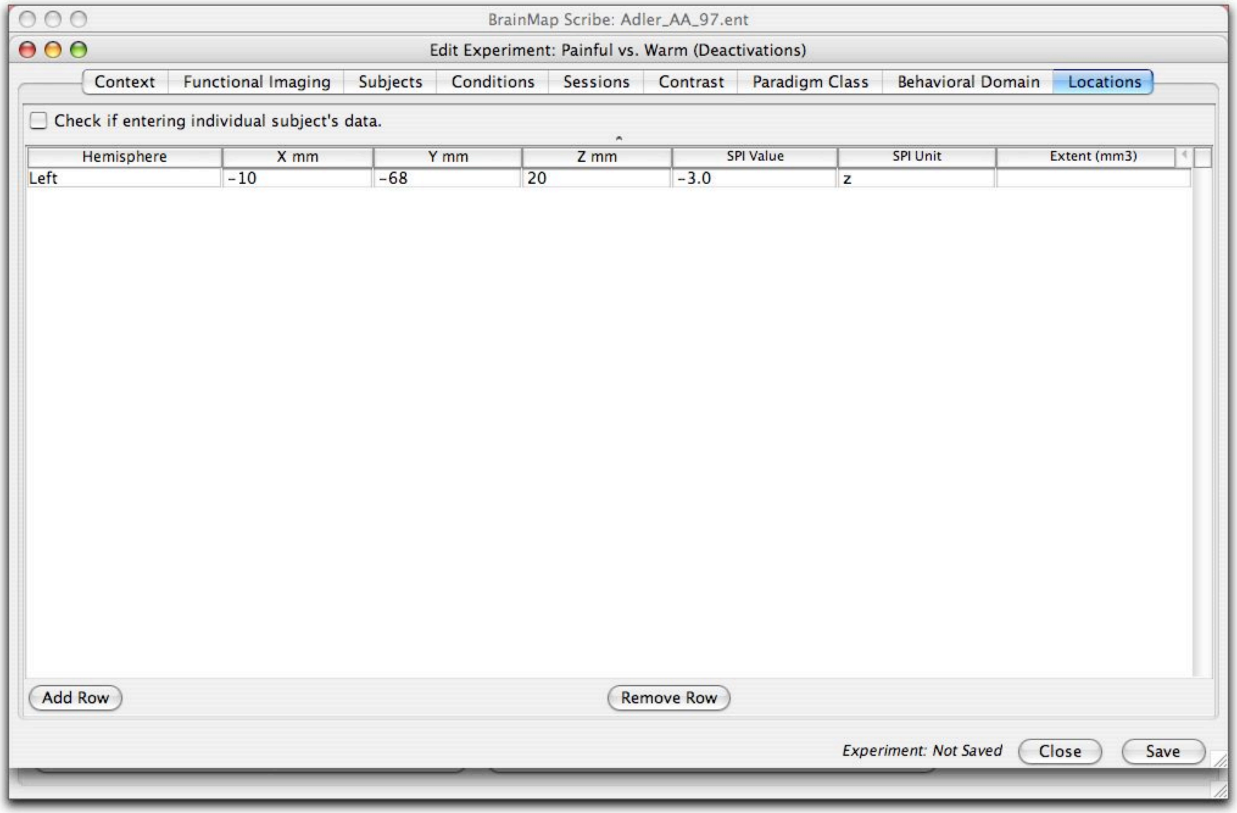

Check if entering individual subject's data
Check this box if the coordinates entered are for individual subjects, as opposed to group mean data.

Hemisphere
Adhere to the standard that **left is negative**. Inspect your table carefully to be sure that all coordinates listed from the left hemisphere have x-values that are negative and those from the right hemisphere are positive. If not, switch the signs of your x-values before entering them into the table.

X mm, Y mm, Z mm
Enter the x-, y-, and z-values as printed in the table (with the exception as noted above).

SPI Value
Enter the SPI value (statistic) reported in the paper, for example: 'z', 't', or 'r'. The z-score or t-statistic can be the mean or the maximum value for the cluster.

SPI Unit
Indicate the variable (z, t, r) entered in the 'SPI Value' column. Do not enter voxel-wise *P* values in the SPI columns.

Extent (mm^3)

Report the **extent** (also referred to as **volume** or **size**) of the activation recorded in cubic millimeters. This number usually ranges from the low hundreds to 1000 or 2000. Do not record the number of voxels (not in units of mm^3).

- 1 ml = 1 cc = 1 cm^3 = 1000 mm^3
- 1 μl = 1 mm^3

For other conversions, please see: http://www.onlineconversion.com/volume.htm.

Results Synopsis

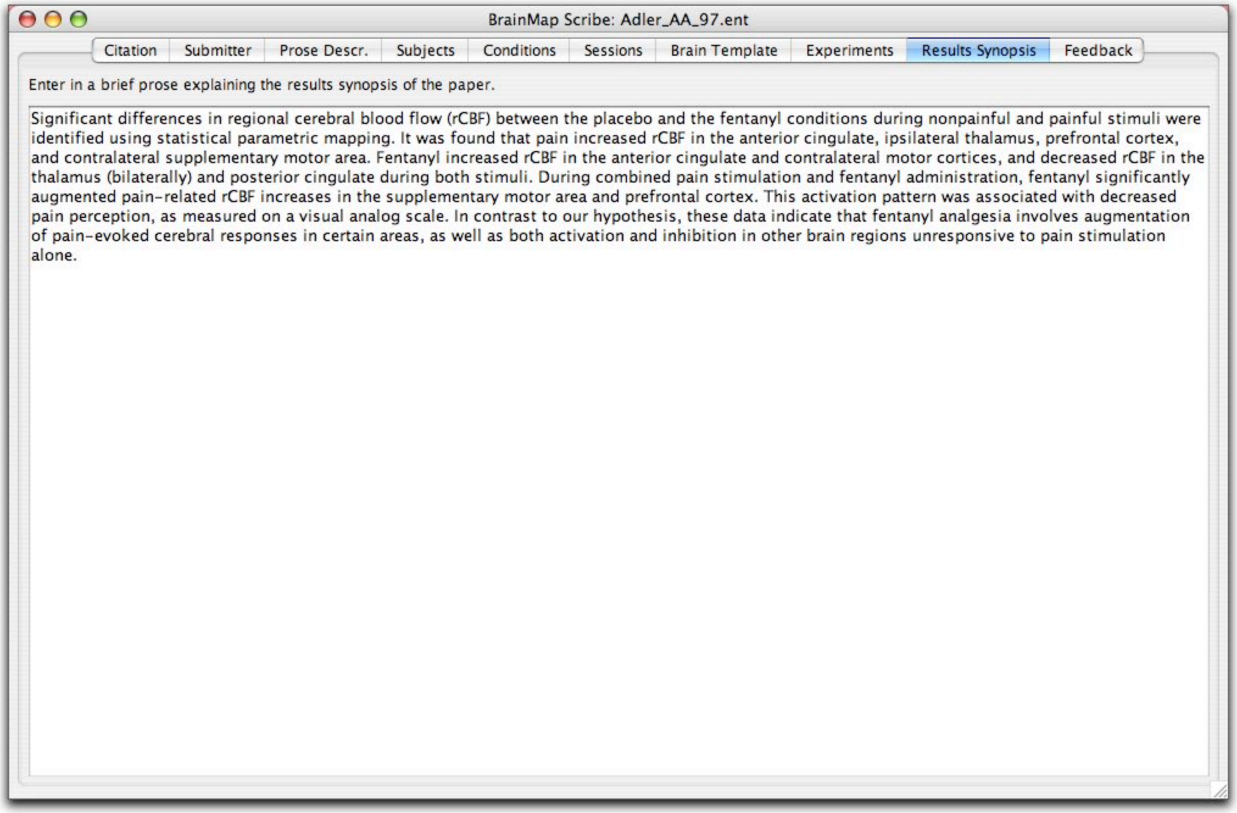

To enter the results synopsis of a paper, copy the published abstract from Medline or PubMed. Delete the first sentences that deal with the introduction, methods, and design of the study and leave only the sentences concerning the results and conclusions. DO NOT edit the synopsis to include your own interpretation of the study.

Feedback

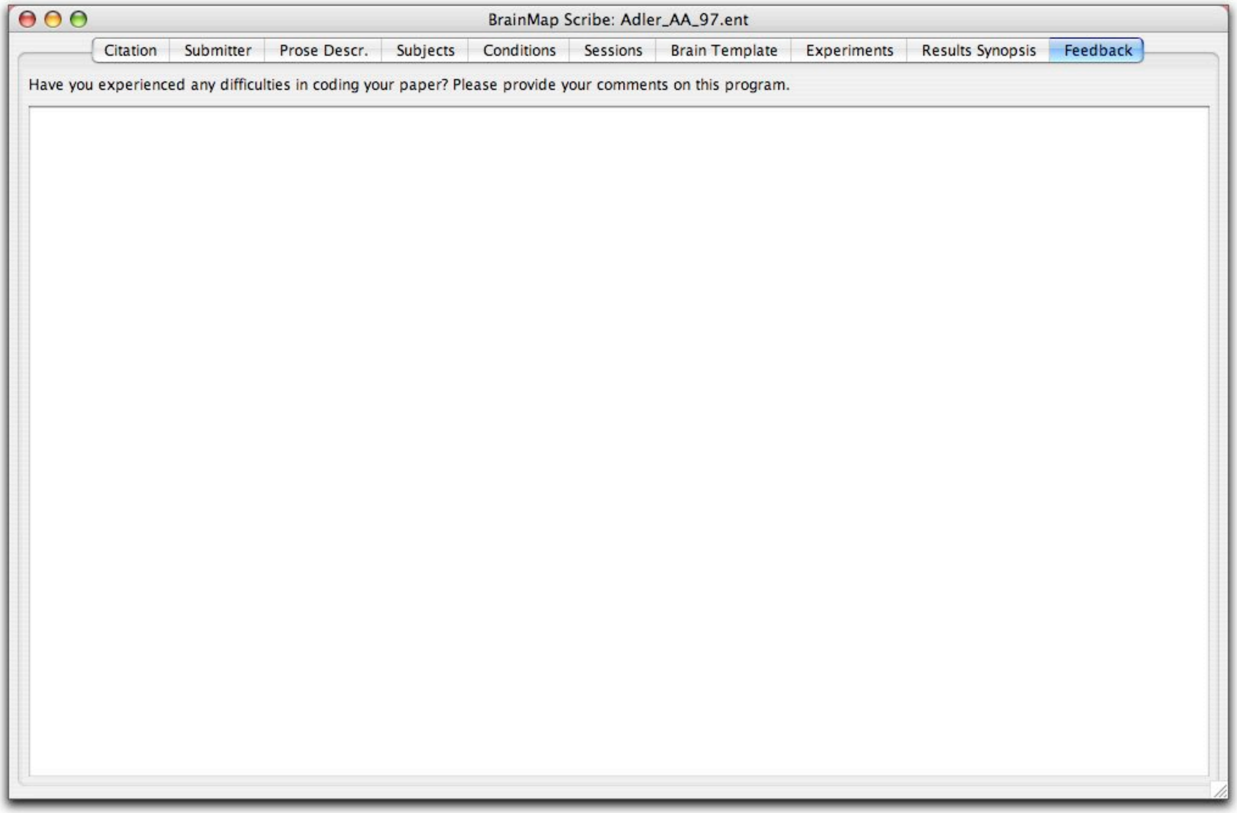

Please use this window to record any questions or comments you may have on the paper.

Save BrainMap Database Submission

After all experiments have been entered, save the BrainMap functional database submission by clicking on the top left program menu: 'File' → 'Save As'. This saves the information in an .ent file.

Enter the file name in the following format: "Author_Journal_Year". Journal names should be abbreviated and years listed as the last two digits of the published year, for example, "Lee_HBM_02.ent".

When you are finished, email your .ent file and a .pdf of the original article to the BrainMap Research Assistant http://brainmap.org/contact.html for review and insertion into the database.

Voxel-Based Morphometry Neuroimaging Submissions

The interface for VBM submissions is very similar to that for functional submissions. Here, we highlight the differences:

- The Citation and Submitter panels are identical to those previously described.
- Due to the reduced complexity of VBM papers, there is no Prose Description. Instead, we ask that you paste in the published abstract for your submission.
- The Subjects panel has a few minor modifications, based on the details for the subject groups.
- The Sessions panel is the same as previously described.
- A new panel, VBM Analysis, has been added for VBM submissions.
- The Experiments panel is greatly simplified in comparison to functional submissions.
- The Feedback panel is the same as previously described.

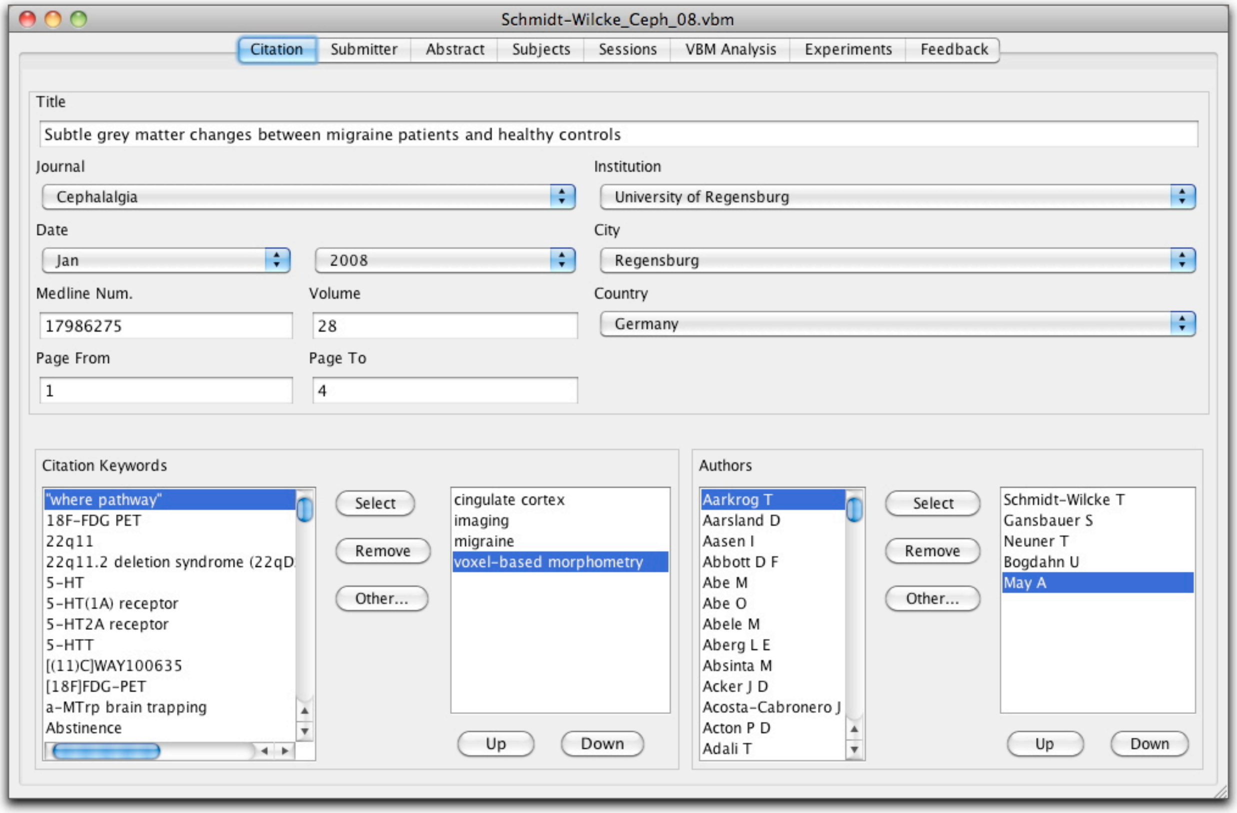

Subjects: Subject Group

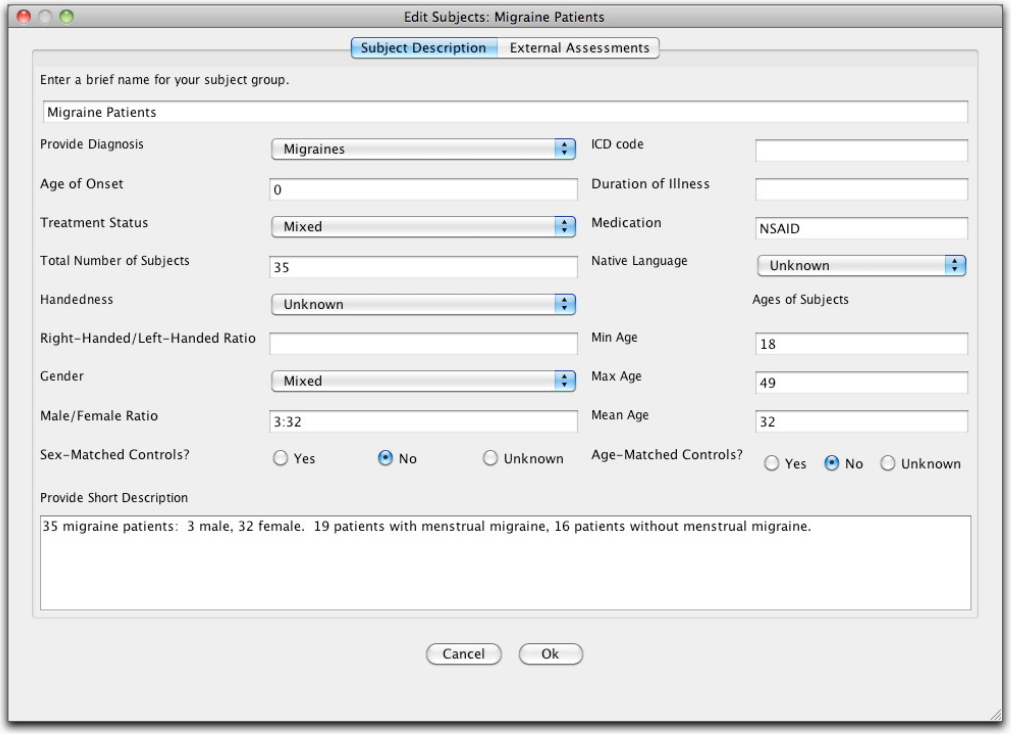

<u>Name of Subject Group</u>
Provide a brief name for the subject group, for example, "Normals".

<u>Provide Diagnosis</u>
Select from the list any medical or psychological conditions.

<u>Age of Onset</u>
Provide the age in years when patients began to experience symptoms of disease/condition. Only integers are allowed.

<u>Treatment Status</u>
Select the treatment ('Medicated' or 'Unmedicated') from the pull-down list. If 'Mixed', provide the number of subjects unmedicated and medicated in the short description.

<u>Total Number of Subjects</u>
Enter the number of subjects studied into the space provided. Only integers are allowed.

<u>Handedness</u>
Choose one option from the menu to describe the handedness of the subject group. Use the 'Mixed' option if the subjects had different handedness.

<u>Right-Handed/Left-Handed Ratio</u>
Provide the number of right-handed and left-handed people (ex. 5:5).

Gender
Select the gender from the pull-down menu. If ‘Mixed’ gender is selected, indicate the number of male and female subjects in the short description.

Male/Female
Provide the number of males and females (ex. 5:5).

Sex-Matched Controls?
Select “Yes” if the non-control (patient) group and control group have the same/similar ratio of males to females.

ICD Code
Most diseases are listed in the International Classification of Diseases (ICD). If the ICD number is unknown (usually the case), then leave this blank.

Duration of Illness
Provide the mean amount of time the patients have been experiencing symptoms (specify days, months, years).

Medication
If subjects are medicated, provide all of the names of the medications. Specify the number of subjects taking each medication in the short description.

Native Language
Choose the native language or languages of the subjects, or add a new language to the list by choosing ‘Other’ and typing into the window that appears.

Min Age
Fill in ‘Min Age’, if reported. Only integers are allowed.

Max Age
Fill in ‘Max Age’, if reported. Only integers are allowed.

Mean Age
Fill in ‘Mean Age’, if reported. Only integers are allowed

Age-Matched Controls?
Select “Yes” if the non-control (patient) group and control group have the same/similar mean age.

External Assessments
This menu is primarily intended for studies wherein an external assessment (Test Score, Physical Characteristic, etc.) was correlated with brain morphometry. If an experiment correlated an external assessment with the volume of brain matter and provided x,y,z locations, enter it here. After selecting the desired option from the menu, click the ‘Select’ button. If the exact external assessment from your paper is not in the list provided, try your best to fit it into one of the provided categories and then provide the exact test name, characteristic, etc. in the short description. Do not add your own external assessment (“other”) to the list unless absolutely necessary. Click “Ok” when complete with the subjects panel to save your information.

VBM Analysis

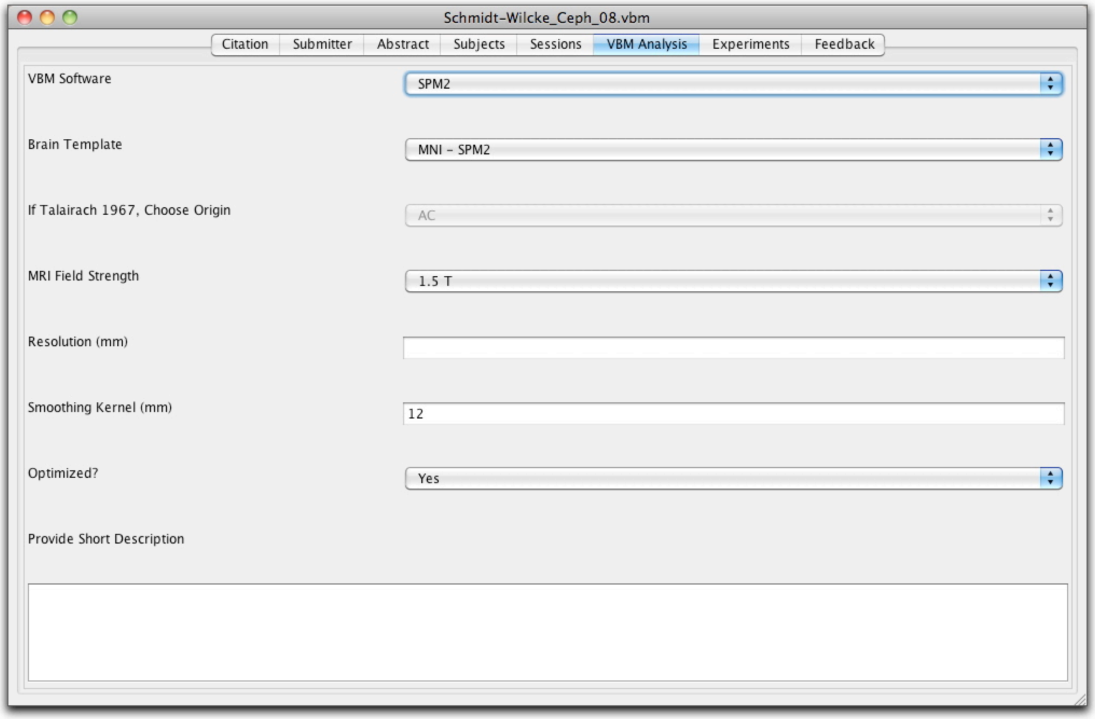

<u>VBM Software</u>
Click on the drop-down menu provided and choose the software used to analyze VBM data.

<u>Brain Template</u>
Click on the drop-down menu provided and choose the brain template used to report the stereotactic coordinates.

<u>MRI Field Strength</u>
Click on the drop-down menu provided and choose the strength of the MRI magnet used to acquire data (in Tesla).

<u>Resolution (mm)</u>
Provide the final voxel size/resolution in mm (e.g., 1x1x1). If this parameter is not provided in the paper, provide the Field of View (FOV) and the slice thickness in mm.

<u>Smoothing Kernel (mm)</u>
Provide the Gaussian smoothing kernel used in mm.

<u>Optimized?</u>
State whether the authors used an “optimized” VBM protocol. If the paper states they used the protocol described in Good et al., 2001 or Asburner and Friston, 2000, then this is “optimized”. Also, if images were modulated by the Jacobian determinant, then this is also considered “optimized”.

<u>Short Description</u>
Enter the software used for spatial normalization.

Experiments

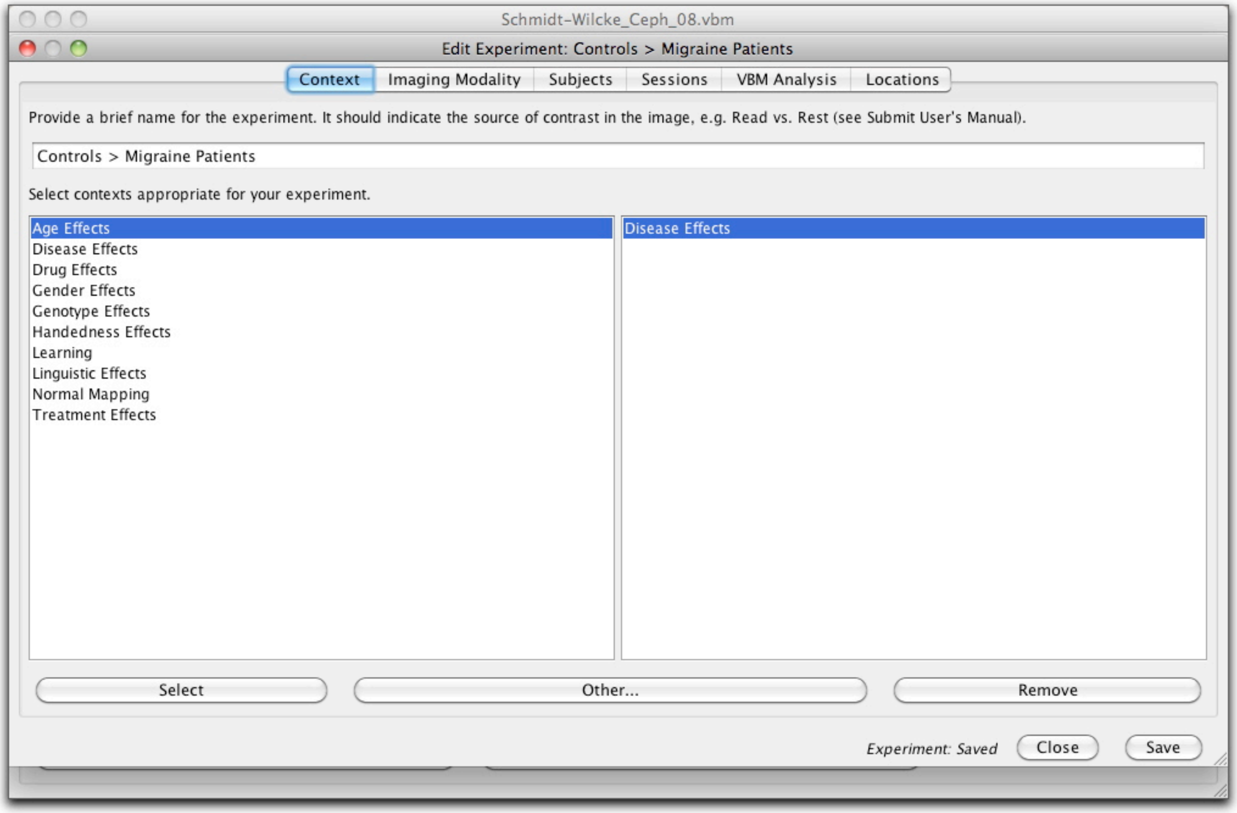

In the Experiments panel, the options for Context, Imaging Modality, Subjects, Sessions, and Locations are the same as for functional submissions. A new panel for VBM Analysis has been added. Due to the simplicity of VBM protocols, there is no panel for Conditions, Paradigm Class, or Behavioral Domain.

When naming VBM experiments, please specific if the locations are Grey Matter or White Matter, and Increases or Decreases.

Experiments: VBM Analysis

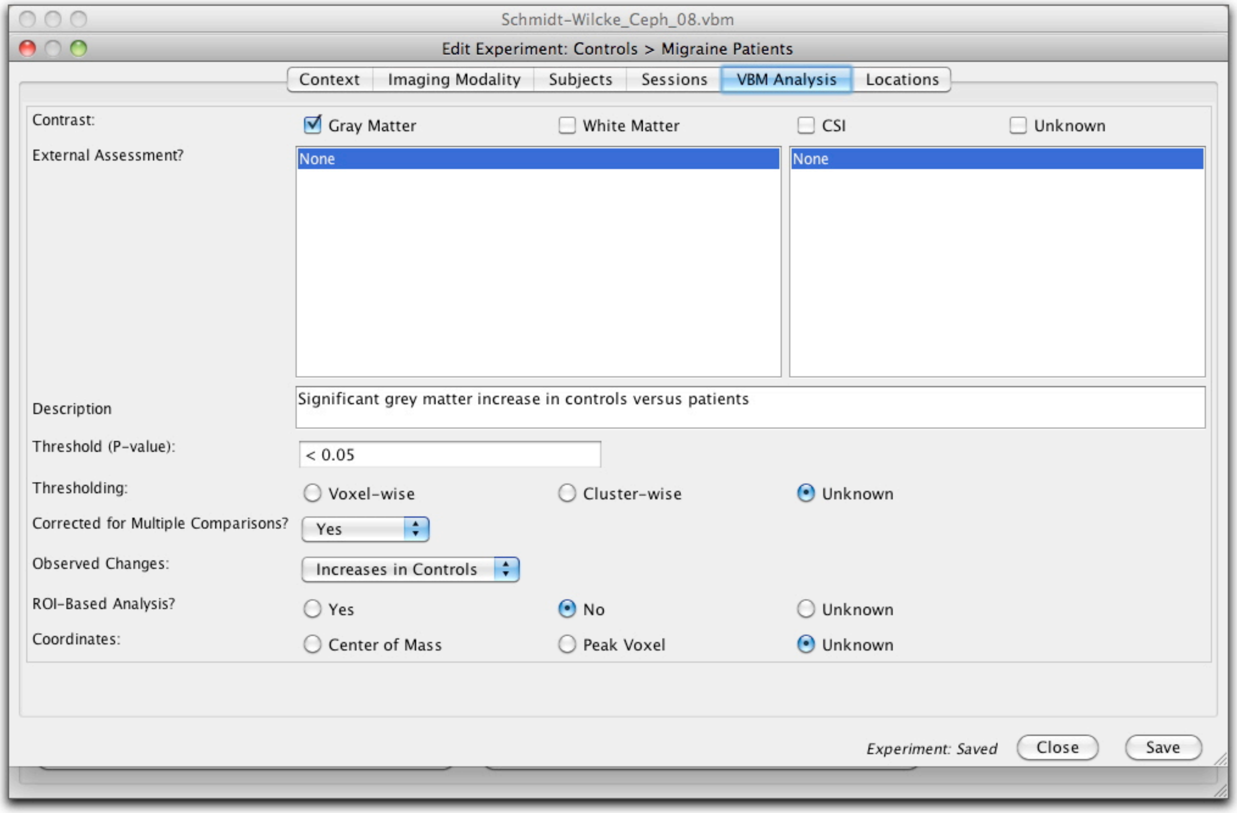

Contrast
Select the tissue contrast for the given experiment.

External Assessment?
This panel contains the names of the external assessments previously provided on the Subjects panel. If the paper included more than one external assessment, choose the one correlated in the given experiment. If no external assessment was correlated then you should select "None".

Description
If relevant, specify the external assessment as you did in the "Subjects" panel. If corrected for multiple comparisons, include the method of correction (e.g., Family-wise Error Correction, etc.") here.

Threshold (P-value)
Provide the p-value used for this experiment. (e.g., <0.01, <0.001, <0.05, etc.)

Thresholding
Select the method of thresholding. If information not provided, select "Unknown".

Corrected for Multiple Comparisons?
Select the answer from the pull-down menu.

Observed Changes

If there is greater brain matter volume in controls (Normals > Patients), then this would be "Increases in Controls", if great brain matter volume in patients (Patients > Normals), then you would select "Increases in Patients". If the experiment contrasts two patient groups or two healthy control groups, choose "Neither".

ROI-Based Analysis?
Select "Yes" for ROI-based analyses that analyzed a small, fixed number of *a priori* brain regions. Select "No" for whole brain analyses.

Coordinates
State whether the published x, y, z coordinates were extracted from the "Center of Mass" or "Peak Voxel" of the observed cluster.

Save VBM BrainMap Database Submission

After all information has been entered, save the BrainMap database submission by clicking on the top left program menu: 'File' → 'Save As'. This saves the information in an .vbm file.

Enter the file name in the following format: "Author_Journal_Year". Journal names should be abbreviated and years listed as the last two digits of the published year, for example, "Lee_HBM_02.vbm".

When you are finished, email your .vbm file and a .pdf of the original article to the BrainMap Research Assistant http://brainmap.org/contact.html for review and insertion into the database.

Troubleshooting

"Scribe says that I'm missing an essential file."

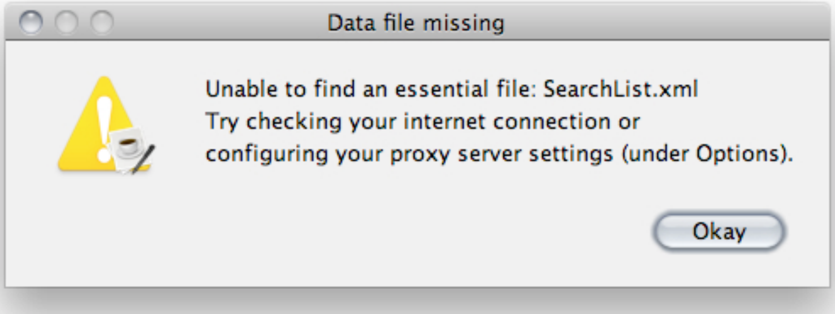

This can indicate one of two things. First, the BrainMap web server may be down for maintenance. In this case, try restarting Scribe in an hour or so. Alternatively, there may be a firewall blocking communication to the BrainMap web server. If a successful connection to BrainMap has been made previously, then please contact BrainMap technical support to check the web server.

If you are behind a restrictive firewall and have a known proxy server, Scribe can use it to access our BrainMap web server. Under File:Options, there is a menu item called "Proxy Settings". You will need to know the IP and valid port of the proxy server.

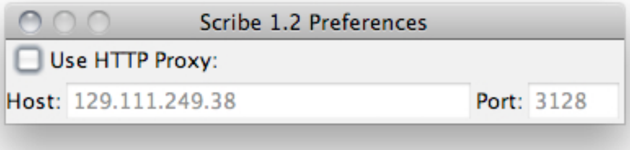

Supplement: Additional file 1 — BrainMap Scribe Software Manual. This file describes the features of the Scribe desktop application for creating BrainMap database entries. [file 1756-0500-4-349-S1.PDF]
