# Supplementary material for: The BrainMap strategy for standardization, sharing, and meta-analysis of neuroimaging data
Source: BMC Res Notes. 2011 Sep 9;4:349. doi: 10.1186/1756-0500-4-349 (PMC3180707; doi:10.1186/1756-0500-4-349)
Supplement: Additional file 2 — BrainMap Sleuth Software Manual. This file describes the features of the Sleuth desktop application for searching, retrieving, and visualizing data archived in the BrainMap databases. [file 1756-0500-4-349-S2.PDF]

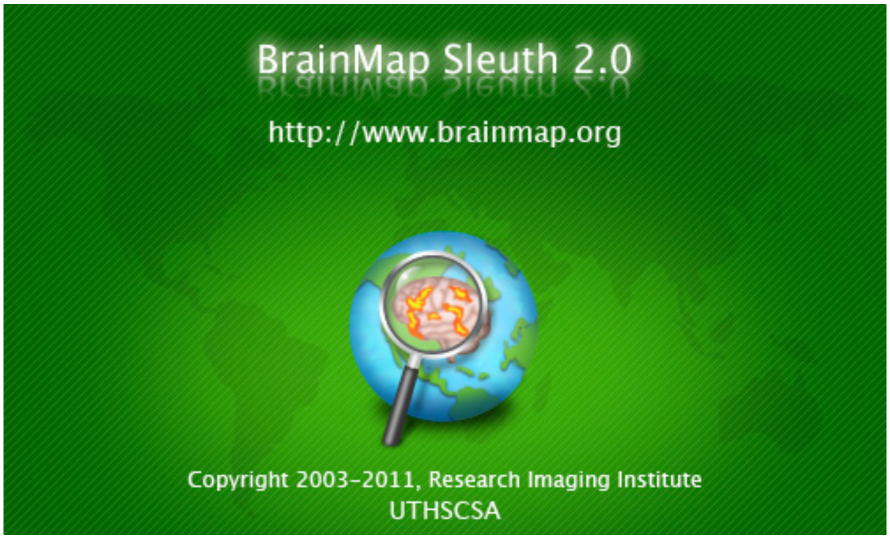

User Manual for Sleuth 2.0

http://brainmap.org

Angela R. Laird, Ph.D.
Research Imaging Institute, UT Health Science Center San Antonio

BrainMap Development Team:
Peter T. Fox, M.D.
Angela R. Laird, Ph.D.
Simon B. Eickhoff, M.D.
Jack L. Lancaster, Ph.D.
Mick Fox, Programmer Analyst
Angela M. Uecker, Programmer Analyst
Kimberly L. Ray, Research Assistant,
Juan J. Saenz, Jr., Research Assistant

Updated 20 May 2011

About Sleuth

BrainMap is an online database of functional neuroimaging results in the form of stereotactic (x,y,z) coordinates. Along with these coordinates, BrainMap also archives each paper's associated meta-data, which includes information on subjects, conditions, experimental paradigms, etc. The software application Sleuth can be downloaded from BrainMap's website, http://brainmap.org, and is used to search the database for experiments of interest and view the relevant search results in a standard brain space (Talairach or MNI).

Logging In

Once Sleuth has been downloaded and installed on your computer, you must visit our website in order to obtain a username and password: http://brainmap.org/sleuth/account.html. Next, launch Sleuth and type in your login information in the dialog box that appears. After the initial login, Sleuth will store your username and password so that you will not have to enter it each time. If you prefer to manually login, you can change this setting in the Preferences menu.

Performing Searches

Sleuth has four main panels: Search, Results, Workspace, and Plot.

Search

To perform a search of the BrainMap database, first choose which database you would like to search, functional or voxel-based morphometry ❶Fig.1. The graphical user interface of Sleuth has been redesigned, and now allows users to build searches with multiple criteria using an interface that was inspired by the playlist building feature in Apple's *iTunes* software (http://www.apple.com/itunes). You may select your desired search criteria from the main interface ❷Fig.1: "Citation", "Subjects", "Conditions", "Experiments, "Locations" for the functional database, and "Citation", "Subjects", "Conditions", "Analysis, "Locations" for the VBM database. The search criteria are further explained below. A hierarchical search criteria feature has been implemented in version 2 of Sleuth, similar to the iTunes playlist building feature. Multiple search criteria may be added by clicking on the "+" button to the left of the search criteria list (or removed using the "-" button). When you have finished making your search selections, hit "Search" ❸Fig.1 and you will be taken on the second panel of Sleuth.

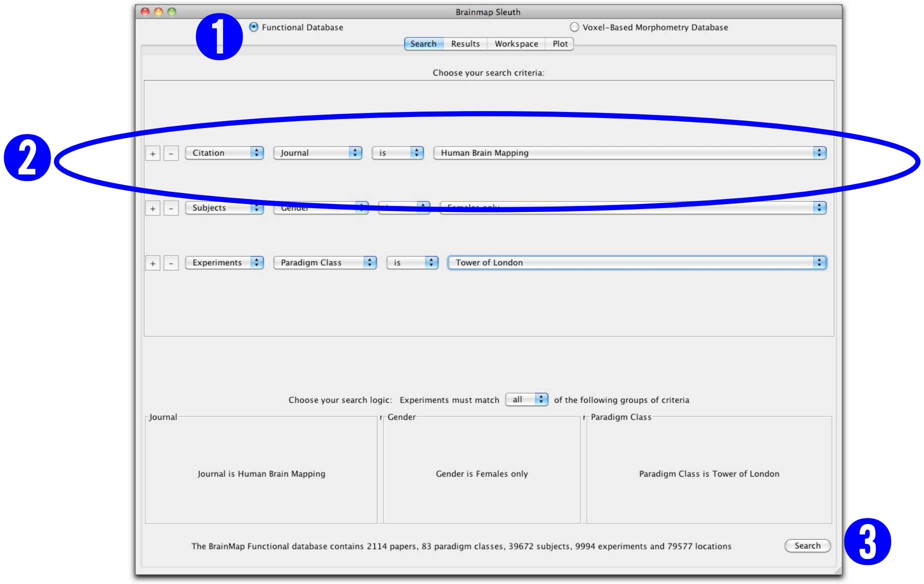

Figure 1. The Search Panel.

Results

Once your search has been completed, a list of all papers matching your criteria will be available for preview in the Results panel. This panel is intended to screen your searches by viewing limited information on each paper returned by your search. The top line of this panel ❶Fig.2 informs you how many papers and experiments match your search criteria. All of the returned papers are listed below ❷Fig.2 where you may click on whichever paper you wish to preview. To select multiple papers at once, ⌘-click (Mac) or ctrl-click (PC). To the right ❸Fig.2 you may select what you wish to view about the paper. Limited information can be viewed on Citation, Prose Description, and Experiment Info. The Prose Description summarily describes all conditions involved in the experiments as well as the experiments themselves (typically the name is based on conditions contrasted). Experiment Info lists all experiments, how many subjects were involved in each experiment, and the number of coordinates. The experiments are viewed in green when they match the search criteria and red when they do not. At the bottom is the preview area ❹Fig.2 where you view the information selected in ❸Fig.2. To download the papers of interest into your workspace, check the box next the appropriate papers in the Download column, and click "Download Checked"; they will then be available for you under the Workspace tab. Alternatively, you can choose "Download All" to get all papers. There is a "Clear Results" button at the bottom left. Be aware that this clears all results, checked or not.

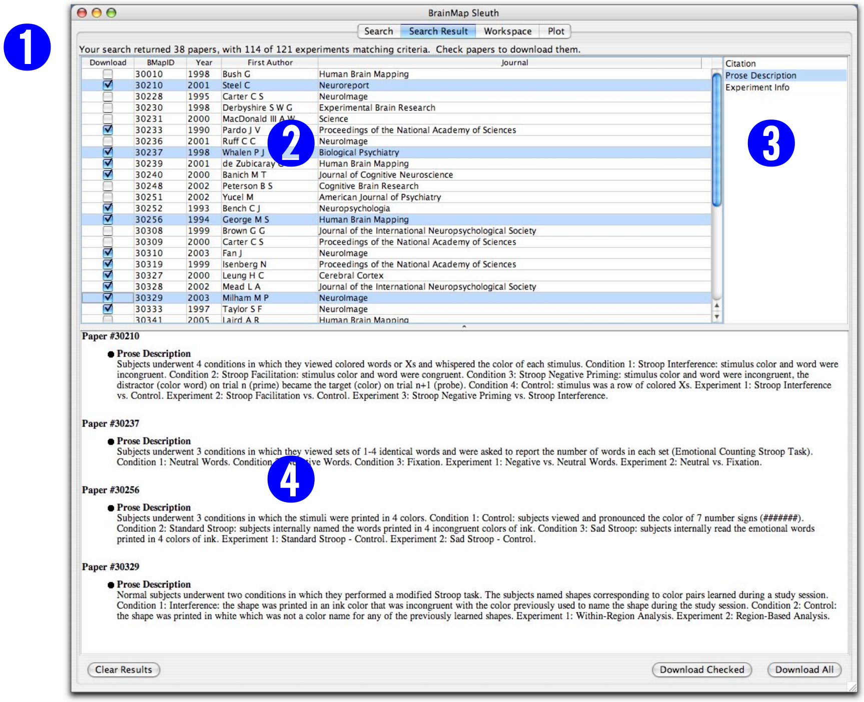

Figure 2. The Search Results Panel.

Workspace

In this panel you will find complete information on each paper and each experiment. A summary of the workspace is listed on the top line of the panel ❶Fig.3: number of papers, experiments, conditions, and locations. The workspace contents and vital statistics are shown in the top portion of the panel ❷Fig.3. Information listed here includes: BrainMap ID, year of publication, first author, journal, experiment name, behavioral domain, and number of coordinates (no behavioral domain is listed for VBM studies). Area ❸Fig.3 allows you to choose what information you wish to view about each experiment, including Citation, Submitter, Prose Description, Subjects, Conditions, Brain Template, Experiments, and Results Synopsis (a modified listing is available for VBM studies). The information contained in Prose Description and Subjects are exactly the same as that previewed under Search Result. However, the Citation information is now much more detailed, as is the

Experiments information. Particularly valuable information that can be viewed under Experiments, including: Paradigm Class, Conditions, Contrast, etc., as well as all the coordinates reported (Talairach or MNI), converted Talairach coordinates, the z- or t-scores, and a Talairach Daemon label for each converted coordinate. The Results Synopsis is taken from the pertinent section of the paper's published abstract. All of this selected information may be viewed in the bottom portion of the workspace ❹Fig.3. You may use ⌘-click (Mac) or ctrl-click (PC) to make multiple selections at once.

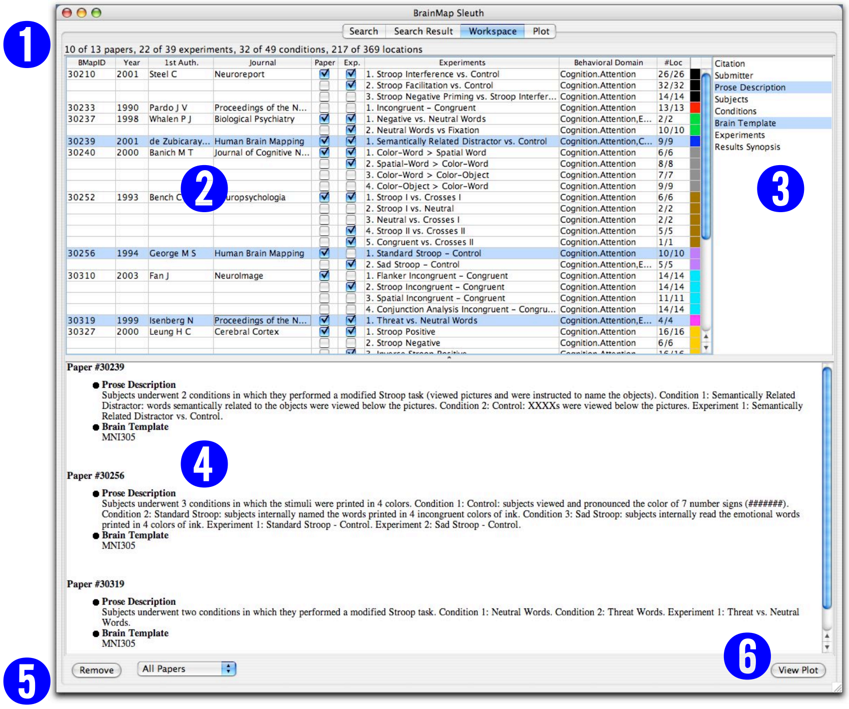

Figure 3. The Workspace Panel.

In the workspace contents ❷Fig.3, there are two columns marked "Paper" and "Exp". Here, you may decide which experiments you wish to retain and plot or which you wish to discard by checking or unchecking them. By default, every experiment that matches your search criteria is retained. There is a "Remove" option at the bottom left corner ❺Fig.3 that can be used to remove unplotted (unchecked) papers or all papers in the workspace. Once you are done making your selections here, you may move on to the Plot panel by clicking on "View Plot" at the lower right corner ❻Fig.3.

Plot

In the next panel you can view a plot of all the coordinates in your workspace, color-coded by paper. By default, the orientation is axial, but can be changed to coronal or sagittal using the appropriate button on the bottom left ❶Fig.4. To the right of those buttons is a box labeled "Current Location (mm)" ❷Fig.4. This box reports the coordinate that your mouse hovers over.

Just to the right of the plot you'll see three scroll bars ❸Fig.4. The middle scroll bar (blue scroll bar for PCs) adjusts which slice of the brain you wish to view (for example, scrolling when you're viewing axial slices moves you up and down through the slices, and scrolling when you're viewing coronal slices moves you forward and back). The far left and right scroll bars (green and red scroll bar for PCs) adjust the slice thickness. You can either adjust the left (green) and right (red) scroll bars by dragging them, or you may type in the appropriate value under the box labeled "Current Slice".

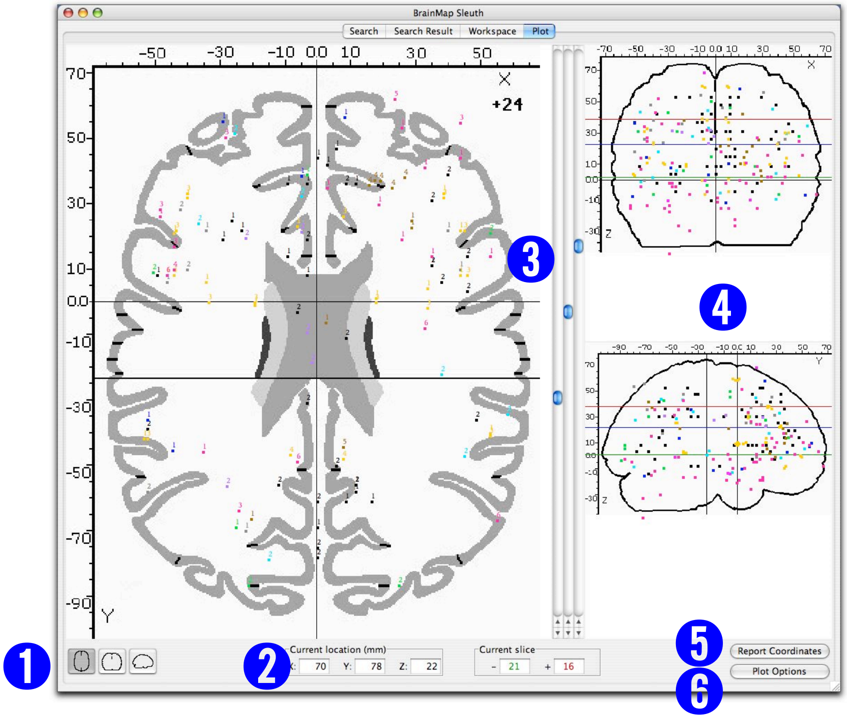

Figure 4. The Plot Panel.

The two plots on the right side are compressed versions of the two other orientations showing all of the coordinates in the paper ❹Fig.4. At the bottom right are two buttons that bring up new dialog windows, described below.

User Options for Plotting

<u>Coordinate Reporter</u>

This dialog window ❺Fig.4 (also Fig.5) reports the x,y,z coordinates and the Talairach Daemon label of whatever location you click on in the Plot panel. In addition, the Coordinate Reporter will tell you the associated BrainMap ID, experiment number, and experiment name that the coordinate is from. If you click on a location in the Plot panel that does not contain a BrainMap coordinate, you will only be shown the Talairach Daemon label of that location, no other information will be returned.

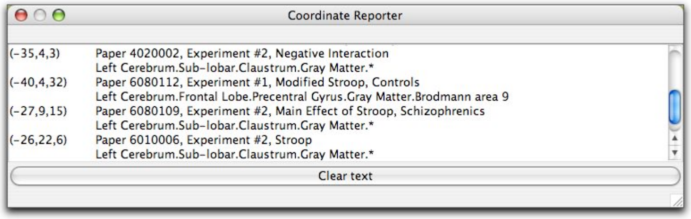

Figure 5. The Coordinate Reporter.

<u>Plot Options</u>

This dialog window ❻Fig.4 (also Fig.6) allows you to set options on how the coordinates appear in the Workspace, as well as filter coordinates. Experiments are selected in the top left portion of the window ❶Fig.6. The coordinates contained in these experiments are shown below ❷Fig.6, and you may choose to check or uncheck the coordinates that are plotted. By default all coordinates in an experiment are plotted. To the right of the coordinate locations is an Edit Filters button ❸Fig.6, which allows you to filter the plotted coordinates by

regions of interest (ROIs). Below the Edit Filters button is a small section for customizing how the coordinates appear in the workspace, including the color of the coordinates, the shape, the font, and the font size ❹Fig.6. There is radio button "Apply to whole paper", which if checked, changes the appearance of all experiments from the same paper. This can retain consistency in how the coordinates from the same paper look, but you may wish to uncheck it if you wish you distinguish between the experiments.

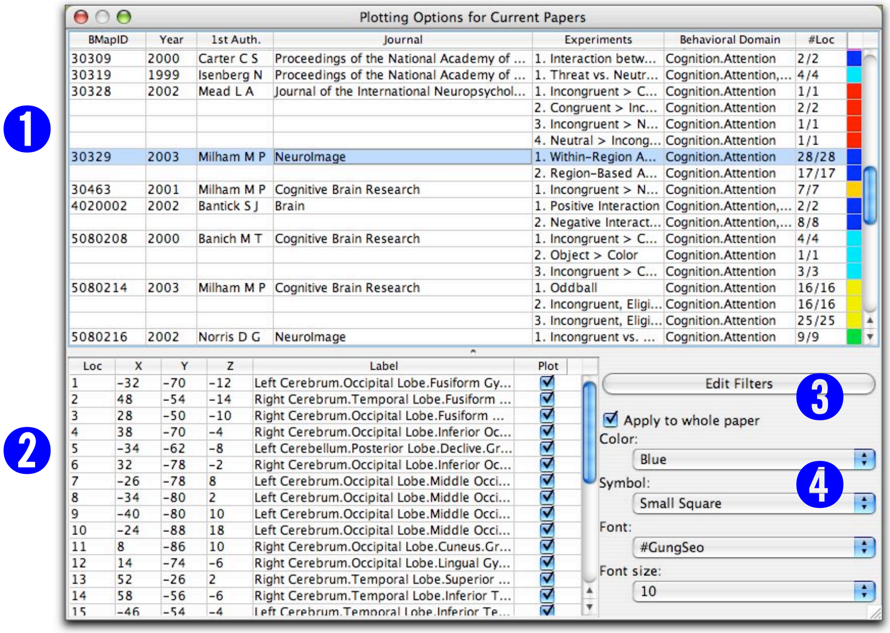

Figure 6. The Plot Options Panel.

Search Criteria Options

BrainMap includes a large amount of metadata that completely describes the content and context of a functional neuroimaging experiment. For functional studies, BrainMap searches are broken into five groups based on what type of search is desired: "Citation", "Subjects", "Conditions", "Experiments, or "Locations". Below, these options are described.

<u>Citation</u>

Citation searches are available on criteria relevant to information on the authors, BrainMap ID, date published, institution, journal, published keyword, and Medline number Fig.7. To add an item to your search query, select the appropriate category and the corresponding search criteria from the drop-down list. Several of these lists, such as authors, may be quite long. Logic operators are found at the bottom to refine your search as to whether the experiments must match "all" or "any" of the chosen criteria. To add search criteria, use the "+" button; to clear any search queries, use the "-" button.

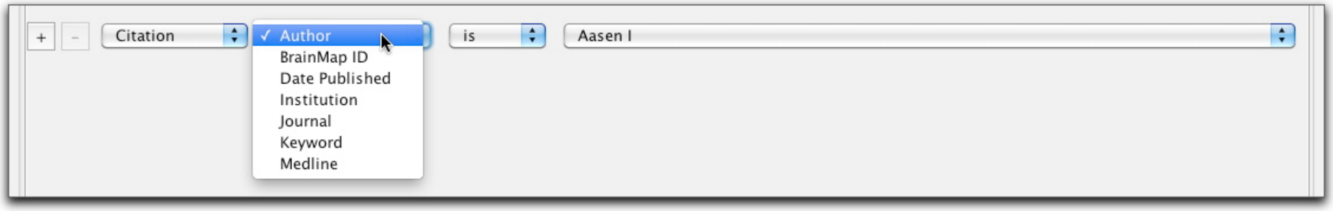

Figure 7. Citation Search Criteria.

Subjects

Subjects searches are available based on diagnosis, ICD code, handedness, native language, gender, subject size range, and age range Fig.8. When searching for a range, be aware that you must construct separate search criteria for the minimum and maximum values using the "is less than" and "is more than" options.

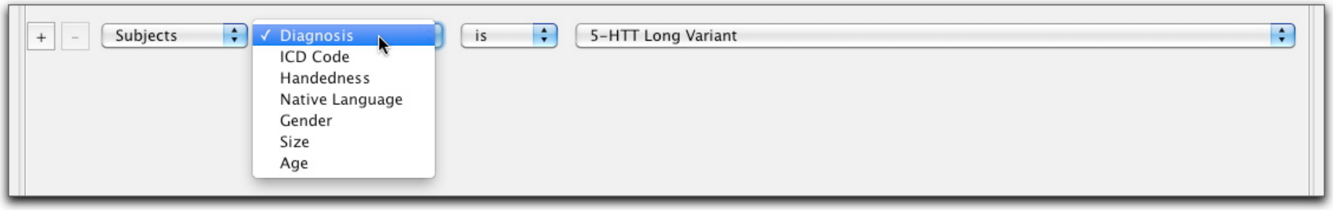

Figure 8. Subjects Search Criteria.

Conditions

Condition searches are available based on stimulus modality and type, overt response modality and type, instructions, and external variable Fig.9. It should also be noted that the response modality and type strictly refer to **overt** responses. Covert verb generation, for example, should fall under Response Modality: None; Response Type: None; Instructions: Generate.

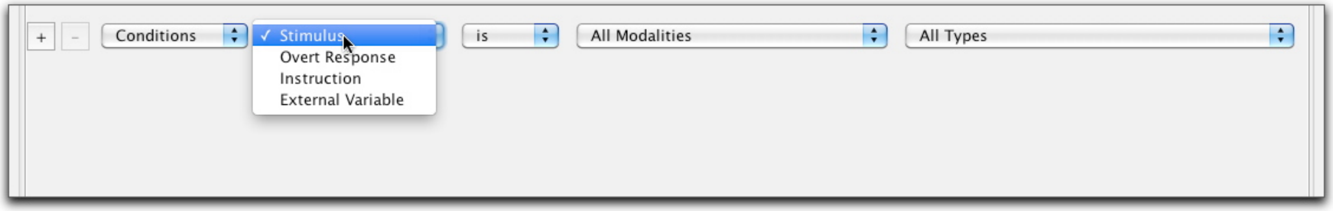

Figure 9. Conditions Search Criteria.

Experiments

Experiment searches are unique to the functional database and can be performed on criteria related to context, paradigm class, experiment contrast, behavioral domain, and imaging modality Fig.10. Behavioral domain is divided into 6 general categories that can be further divided into subcategories. To view all subcategories, double click on the appropriate category. For example, double clicking on Action yields subcategories including Execution, Imagination, Inhibition, etc.

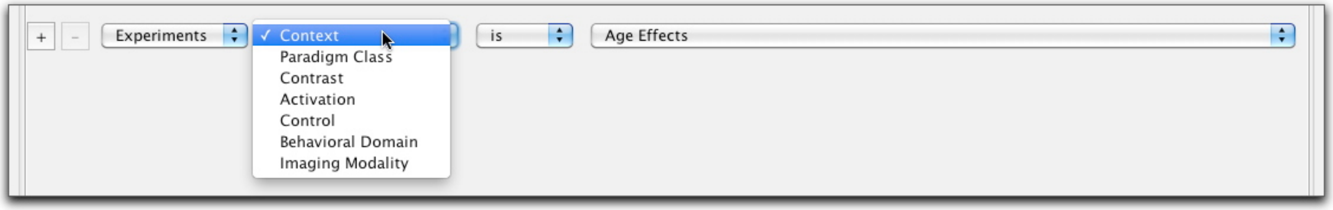

Figure 10. Experiments Search Criteria (Functional Only).

Analysis

Analysis searches are unique to the VBM database and can be performed on criteria related to VBM software, MRI field strength, and brain template Fig.11.

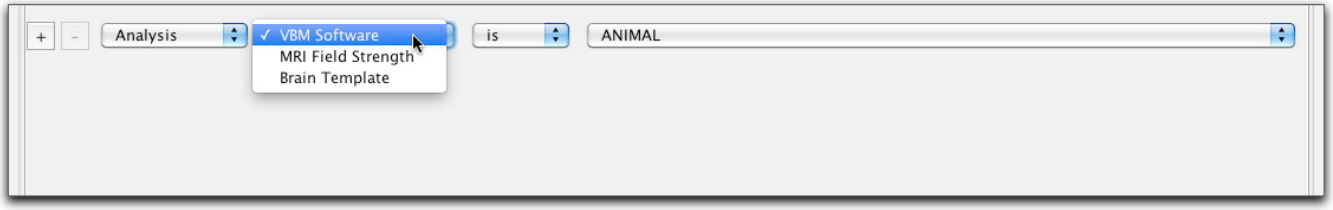

Figure 11. Analysis Search Criteria (VBM Only).

Locations

The coordinates in BrainMap can be searched either by a user-defined rectangular or spherical ROI ("Rectangular ROI"), anatomical label defined by the Talairach Daemon label ("TD Label"), or most recently, by arbitrary-shaped ROI image in Talairach ("Talairach Image") or MNI space ("MNI Image") Fig.12. Rectangular ROIs must be defined under "ROI Options" in the main toolbar (see below). Selecting the Talairach Image or MNI Image options will open a dialog window to allow the appropriate image to be selected. To carry out these image-based ROI searches, all user-originated files must conform to a strict format: ROIs must be formatted as binary NIfTI (http://nifti.nimh.nih.gov) images with 1x1x1 mm^3 resolution, and the ROI must not extend across more than 500 voxels. These stringent requirements are enforced to ensure a timely response from the database; more advanced hardware solutions are currently being evaluated to reduce these technical limitations and allow rapid image-based ROI searches of greater volume.

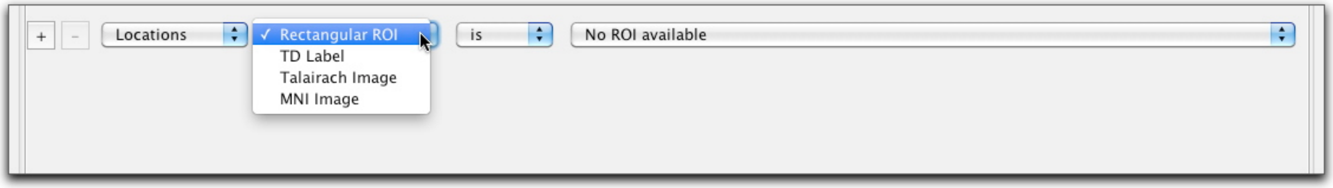

Figure 12. Locations Search Criteria.

Main Menu Items

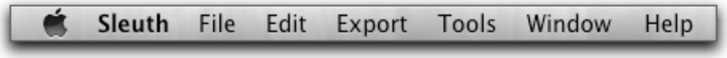

Figure 13. The Main Application Menu.

Sleuth

About Sleuth: This menu item contains basic information about Sleuth, such as homepage, version number, and copyright date.

Preferences: This menu item sets certain user preferences, such as remembering your login name and logging in automatically when the application is opened Fig.14. Hotkey: ⌘-, (Mac) or ctrl-, (PC). This is also where users can select their preferred standard brain space (Talairach or MNI).

Sleuth can now be used with HTTP proxies. This information is set in the Preferences. Your system administrator can help you with the details for this setting.

Quit Sleuth: This menu item exits you out of Sleuth. Hotkey: ⌘-Q (Mac) or ctrl-Q (PC).

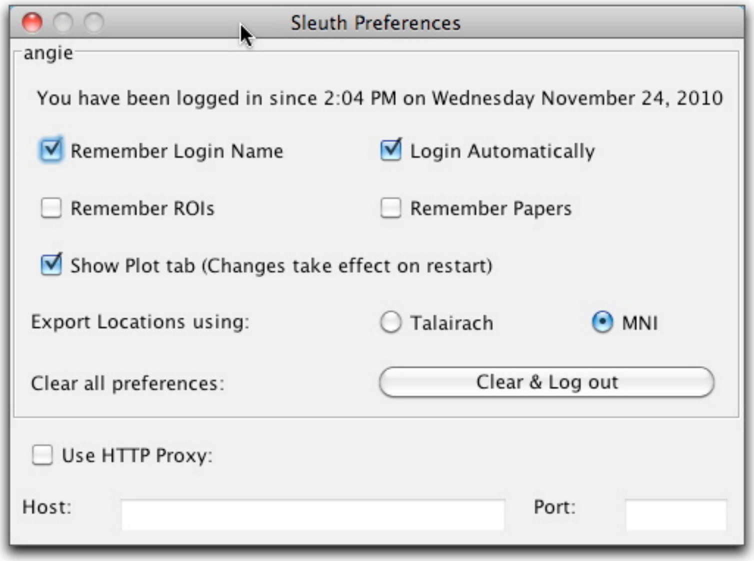

Figure 14. Preferences.

File

Log out: This menu item logs you out of Sleuth. Hotkey: ⌘-L (Mac) or ctrl-L (PC).

Open: This menu item allows you to open saved workspaces (.xml files), or locations (.txt files of x,y,z coordinates). Alternatively, you can open one of BrainMap's data files in .ent format that have been created using the application Scribe. Hotkey: ⌘-O (Mac) or ctrl-O (PC).

Save Workspace: This menu item saves your workspaces. By default, these workspaces are saved in BrainMap's .work format. However, since these workspace files are saved in .xml format, you may also specify a name with the .xml file extension. Hotkey: ⌘-S (Mac) or ctrl-S (PC).

Edit

This menu item allows you to "Select All" or "Select None" and is useful when selecting which experiments should be downloaded into the workspace.

Export

Citations (Endnote): This menu item exports the citation information from the papers in your workspace in .txt format, in a style that can be imported into the Endnote reference manager software. When importing these citations into Endnote, use the Import Option of "Endnote Import" with no text translation.

Locations (GingerALE Text): This menu item exports the locations (x,y,z coordinates) contained in your workspace in .txt format. Coordinates will be grouped by experiment and labeled by first author name, year, and experiment name. This metadata is commented out in the text file by a "//" so that the file may be correctly read back into the workspace at a later time for ALE meta-analysis purposes. If you have selected "MNI" under Preferences, then your coordinates will be exported in MNI space.

Locations (NIfTI Image): This menu item exports the locations (x,y,z coordinates) contained in your workspace in image format (.nii). This file can be viewed with our anatomical template (Colin1.1.nii) that is available on our website (www.brainmap.org/ale). In the image, each voxel that contains a foci from your workspace is assigned

a value corresponding to its experiment number. For any voxel in which more than one coordinate appears, we assign a value of n+1, where n equals the total number of experiments in your workspace. For example, if you have 5 experiments in your workspace and choose Export → Locations (Image), then the file that is written will have values that vary from 0 (no coordinates) to 5. If, however, identical coordinates are given in two or more experiments, the voxel corresponding to that coordinate will be assigned a value of 6. If you have selected "MNI" under Preferences, then your coordinates will be exported in MNI space, and you will need an alternate anatomical template to view this image (Colin27_T1_seg_MNI.nii, also on the ALE website).

Tools

Color Options: This menu item Fig.15 allows access to editing existing colors or adding new colors by specifying RGB values. To change an existing color, select that color from the left ❶Fig.15, make the desired modifications to the RGB values, and click on "Save this color" ❷Fig.15. To add a new color, specify the desired RGB values, name the new color, and click "Add this color" ❸Fig.15. The new color will then appear in the list on the left. The colors in this list are used when plotting coordinates in the Plot panel.

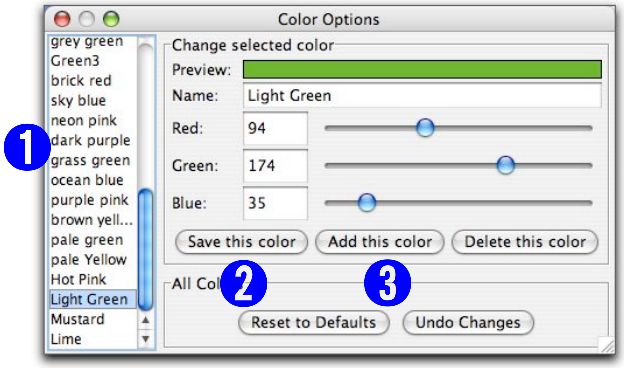

Figure 15. Color Options.

ROI Options: This menu item allows you to define a new ROI with a custom name and color. The ROI drawn will always be a rectangular prism, but can be drawn in one of two ways. The default dialog window allows you to enter the minimum and maximum x,y,z values for your desired ROI dimensions Fig.16, left. Alternatively, you can check the box "Use Cube-Shaped ROI" and draw a ROI cube with side lengths you specify (Width), centered at a given coordinate Fig.16, right. In addition, if you would like to enter your ROI in MNI coordinates instead of Talairach coordinates, then check "Convert coordinates from MNI" and choose the software you used for spatial normalization from the drop-down menu so that we implement the correct transformation from MNI to Talairach space (please see Lancaster et al., 2007). Please note that we no longer use the Brett transform to convert between MNI and Talairach space.

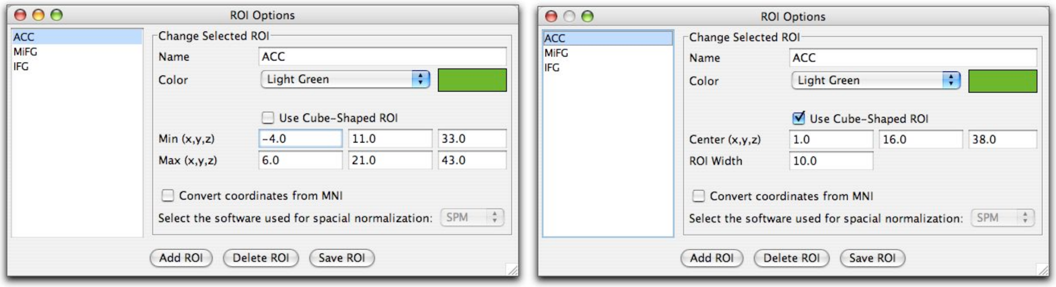

Figure 16. ROI Options: Rectangular (left) and Cubic (right) ROIs.

ROI Statistics: This menu item reports various statistics relevant to all of the ROIs present in the workspace, and the coordinates that are found within said ROIs Fig.17. ROI information includes: ROI name, color, and dimensions. Locations in this ROI are detailed by: BrainMap paper ID, experiment number, x,y,z coordinate values, and Talairach Daemon label.

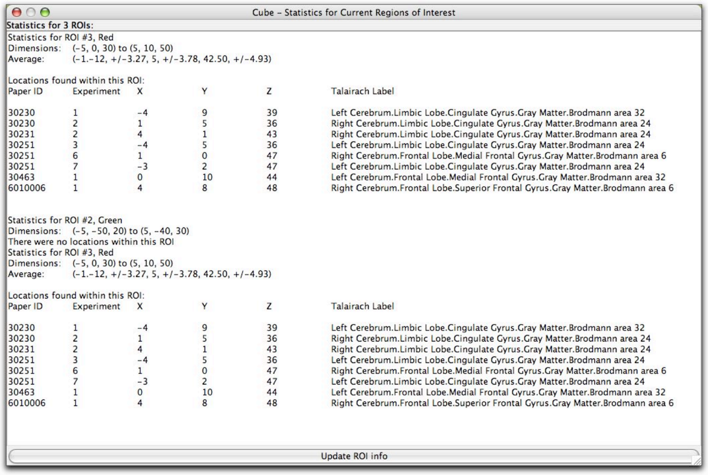

Figure 17. ROI Statistics.

Database Statistics: This menu item shows how many papers, experiments, and coordinates are currently in either the functional or VBM databases Fig.18.

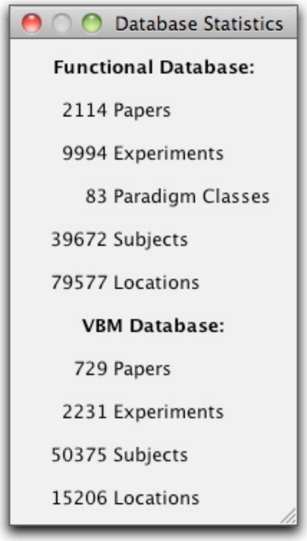

Figure 18. BrainMap Database Statistics.

Behavioral Histogram: This menu item computes a histogram that details the behavioral domain profile of the current workspace Fig.19. On the top is a local histogram, which applies to all the experiments in the workspace (or specific ROIs that are selected in the drop-down panel). On the bottom is a global histogram, which applies to all the papers in the BrainMap database. Colors indicate levels of the behavioral domain hierarchy. Counts for individual domains are viewed in the panel on the left. The default listing of domains includes the domains relevant to the existing workspace. The counts for each domain are cumulative (include counts for all sub-categories), but the histogram bins show only what is in that specific domain. For example, the counts on the

right will include hits for Memory and Working Memory, but the only the bin for Memory will disappear if the checkbox for Memory is unchecked (i.e., the bin for Working Memory will remain visible).

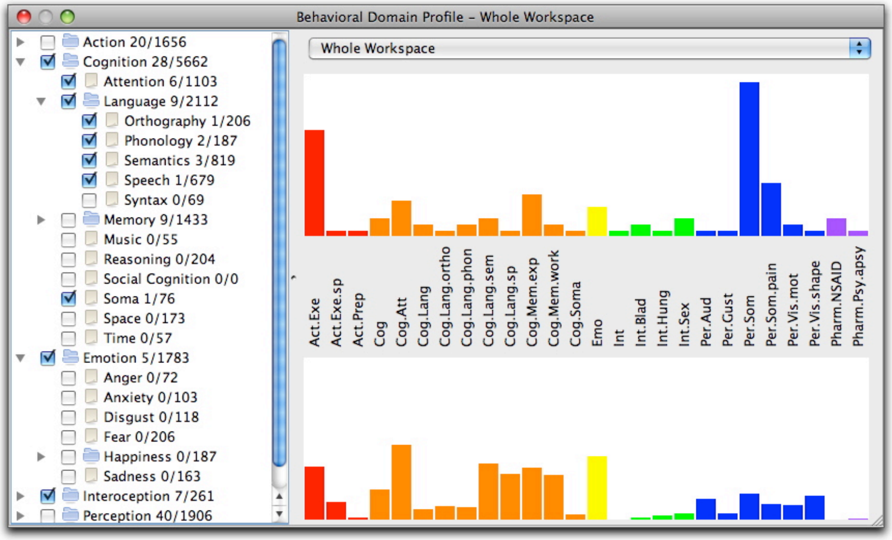

Figure 19. Behavioral Histogram.

Paradigms Histogram: This menu item computes a histogram that details the paradigm class profile of the current workspace Fig.20. Again, the top is a local histogram, which applies to all the experiments in the workspace (or specific ROIs that are selected in the drop-down panel). On the bottom is a global histogram, which applies to all the papers in the BrainMap database. Counts for paradigm classes are viewed in the panel on the left. The default listing of paradigms includes the classes relevant to the existing workspace.

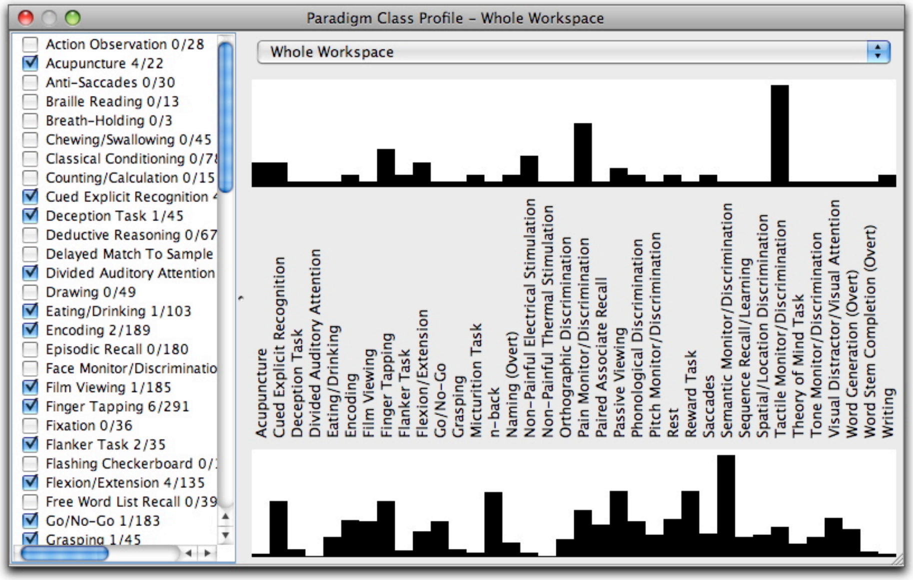

Figure 20. Paradigms Histogram.

Window

Each item in this menu tab takes you to the appropriate panel of Sleuth (Search, Workspace, or Plot).

Hotkey: Search = ⌘-1 (Mac) or ctrl-1 (PC)
Workspace = ⌘-2 (Mac) or ctrl-2 (PC)
Plot = ⌘-3 (Mac) or ctrl-3 (PC)

Help

Debugging Info: This menu item contains Sleuth configuration information in case troubleshooting is necessary.

Check for Updates: This menu item searches the web for Sleuth updates.

Show Read Me: This menu item will show the current readme file for Sleuth. The readme file contains information about installation and version changes. An internet connection is necessary for this menu option.

Show Manual: This menu item will show the current manual for Sleuth (this document). An internet connection is necessary for this menu option.

Show License: This menu item will show the current license information for Sleuth. An internet connection is necessary for this menu option.

Troubleshooting

I downloaded Sleuth and obtained a BrainMap username and password, but when I double-click on the application I get an error that says I cannot connect to the database.

This can indicate one of two things. First, the BrainMap web server may be down for maintenance. Alternatively, there may be a firewall blocking communication to the BrainMap web server. If your problems are caused by a firewall issue, you will see the following error:

java.io.IOException: Server returned HTTP response code: 500 for URL:
http://biad02.uthscsa.edu:9000/sv/servlet/performSql3.GetSearchResult2

The BrainMap web server uses port 80 and port 9000 for all Sleuth communications. If a successful connection to BrainMap has been made previously, then please contact BrainMap technical support to check the web server. Otherwise, your firewall issue can be solved by contacting your network administrator to make sure port 80 and port 9000 are open to http traffic from host www.brainmap.org.

I am interested in performing an ALE meta-analysis for my research. However, many of the papers I'd like to use aren't in the database. What should I do?

You, as a BrainMap user, are eligible to enter papers into our database. If you'd like to perform an ALE meta-analysis in GingerALE, then we definitely recommend that you add your missing papers to BrainMap. To do this you'll have to download our input software application, Scribe (http://brainmap.org/scribe/index.html) and use that software to enter in information about each paper. Also on the above webpage you'll find the user's manual for Scribe and several example .ent files (BrainMap database entry files). You can submit any functional or VBM neuroimaging paper that is of interest to you – the only requirement is that it publish results as stereotactic (x,y,z) coordinates.

Previously, I created and saved a workspace. Today, when I loaded that file I received an error:

"Please re-save this workspace. The database has recently been updated. Please check your workspace for errors and re-save the file."

Can you explain this error?

Occasionally, we find errors in BrainMap entry files. When we find an error, we correct it and reinsert the whole paper into the database. If you've included a file such as this in one of your saved workspaces, you'll receive this error the next time you open that workspace. Your workspace has been revised such that the corrected paper now has all experiments checked, regardless of how it was previously saved in the workspace. Scroll down your workspace and look for any paper(s) with all experiments checked. Then, uncheck any experiments from that paper that you do not want included in the workspace and re-save your workspace to an .xml or .work file.

I received the error: "Unable to download the following papers", followed by a long list of BrainMap ID number. What's going on?

If you're trying to download an extremely large workspace with many papers in it, this may cause stress on our servers. If you see this error message, try re-downloading your workspace. If possible, it might help to be more conservative in your search features so as to download a smaller, more targeted number of papers.

Does BrainMap authorize the reproductions of screen captures from Sleuth to be used in publications?

Yes, we allow screen captures to be published of Sleuth Workspaces or Plots. When doing so, please give BrainMap credit and cite the following paper:

Laird AR, Lancaster JL, Fox PT. (2005). BrainMap: The social evolution of a functional neuroimaging database. Neuroinformatics 3, 65-78.

I need to perform an analysis of coordinate data (x,y,z). Can you send me a text or .xml file of the contents of the BrainMap database?

BrainMap is a registered trademark of the University of Texas. All software and **curated** data contained in or used by the BrainMap database are copyrighted by the University of Texas. BrainMap software and data are available for use, free of charge, for educational and scientific, non-commercial purposes. The BrainMap coding scheme and its taxonomy of experimental design are available for use without restriction. If BrainMap's data, software, or coding strategies are used in scientific publications, we request that one or more relevant publications of the development team be cited, as described above. The BrainMap development team welcomes collaborations. We will provide guidance and assistance in the execution of meta-analyses, upon request. We encourage collaborations that develop new tools for meta-analysis or use BrainMap data to develop or validate other neuroinformatics tools and strategies. We do allow other databases and image-analysis environments to query BrainMap's data, but only with prior permission and as a joint development effort. BrainMap data may not be extracted for inclusion in other databases or redistributed in any manner, without prior written permission.
