# Supplementary material for: The BrainMap strategy for standardization, sharing, and meta-analysis of neuroimaging data
Source: BMC Res Notes. 2011 Sep 9;4:349. doi: 10.1186/1756-0500-4-349 (PMC3180707; doi:10.1186/1756-0500-4-349)
Supplement: Additional file 3 — BrainMap GingerALE Software Manual. This file describes the features of the GingerALE desktop application for performing activation likelihood estimation (ALE) meta-analyses on coordinate-based neuroimaging data. [file 1756-0500-4-349-S3.PDF]

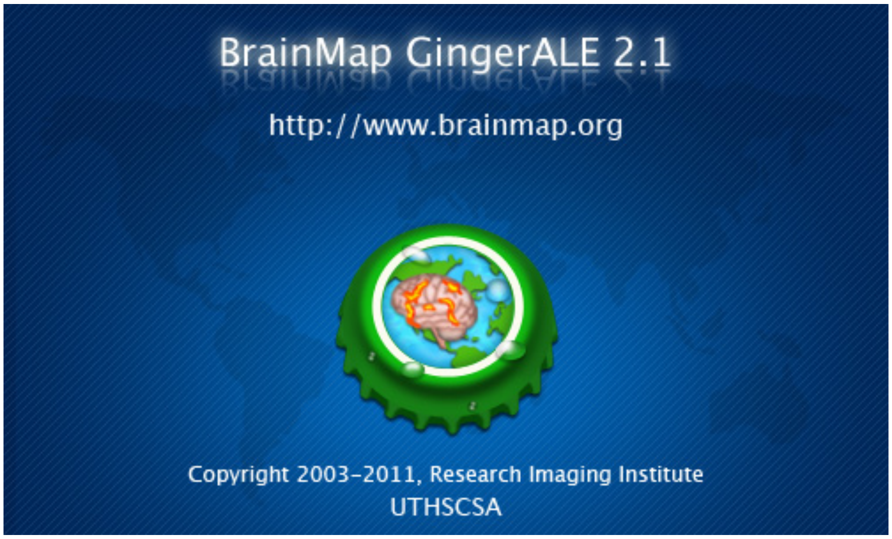

User Manual for GingerALE 2.1

http://brainmap.org

Angela R. Laird, Ph.D.
Research Imaging Institute, UT Health Science Center San Antonio

BrainMap Development Team:
Peter T. Fox, M.D.
Angela R. Laird, Ph.D.
Simon B. Eickhoff, M.D.
Jack L. Lancaster, Ph.D.
Mick Fox, Programmer Analyst
Angela M. Uecker, Programmer Analyst
Kimberly L. Ray, Research Assistant,
Juan J. Saenz, Jr., Research Assistant

Updated 20 May 2011

About GingerALE

GingerALE is used for performing activation (or anatomic) likelihood estimation (ALE) meta-analyses. The ALE meta-analysis method was initially developed by Peter Turkeltaub (Turkeltaub et al., 2002). This method of meta-analysis was adopted by BrainMap in 2003. Several modifications have been made to the ALE algorithm since then, including those described by Laird et al. (2005).

The current version of our software is described by Eickhoff et al. (2009), with a small modification described by Turkeltaub et al. (2011). The full text for these publications is available on www.brainmap.org/pubs.

New Features in GingerALE:

- ALE now supports random effect meta-analyses, rather than fixed effects meta-analyses.
- ALE values are weighted by the sample size of each contributing study.
- Users now have a choice of running their meta-analyses in MNI or Talairach space.
- Subtraction analysis is now included for two sets of foci.
- Streamlined interface that requires fewer buttons.

Performing ALE Meta-Analyses

All output files are written in NIfTI (.nii) format. The input for a meta-analysis in GingerALE is a text file of your foci. This file can be generated by hand, from an excel worksheet, or as an automated export of your workspace in BrainMap Sleuth. It is critical that any manually generated file is created with a format that exactly matches what is exported by Sleuth, including tags for the number of subjects and the standard brain space (e.g., Talairach or MNI). Be sure to include spacing between foci from separate contrasts, but not for each individual foci. Using non-standard formatting in your input foci file can result in empty ALE images.

If you used Sleuth to create a foci file from your workspace, then there is no need to spatially renormalize your MNI coordinates to Talairach space (or vice versa). This conversion is done automatically when the papers are inserted into the BrainMap database using a transform called icbm2tal developed by Lancaster et al. (2007). This new transform provides improved fit over the Brett transform (mni2tal), and improves the accuracy of meta-analyses (Laird et al., 2010). Please note that we no longer use the Brett transform for conversion of coordinates from MNI space to Talairach space; however, this transform is included in GingerALE's software to allow reconversion of published foci.

To load your coordinates into GingerALE, go to File → Open Foci. Navigate to your desired text file and select. If your preferences are set to a standard brain space that does not match the tag in your foci file, you will be prompted to change your coordinate system to match. A dialog window will inform you if any of your foci are located outside the brain mask; typically a small percentage of foci are located outside the mask. If a large number of foci are outside the mask, please check your foci file for errors.

The main window of GingerALE will then confirm for you the name of your foci file and the number of coordinates and experiments contained therein (Fig. 1). The prefix of your foci file will be used when generating output ALE images; you may edit this prefix if you choose.

The ALE meta-analysis procedure follows 3 steps. **First**, GingerALE computes the ALE values for each voxel in the brain and performs a test to determine the null distribution of the ALE statistic at each voxel. We no longer require manually entering the FWHM value, as this parameter has been empirically determined (Eickhoff et al., 2009). **Next**, GingerALE takes the *P* values from the previous step and computes the threshold for the ALE map using the algorithm from Tom Nichols's website (http://www.sph.umich.edu/~nichols/FDR). Choose a False Discovery Rate (q) for the desired level of significance (e.g., 0.05 or 0.01). Also choose a minimum cluster size in

mm^3. **Lastly**, GingerALE performs cluster analysis on the thresholded map, based on the minimum volume that is specified in the previous step. Anatomical labels of final cluster locations are provided by the Talairach Daemon: http://talairach.org.

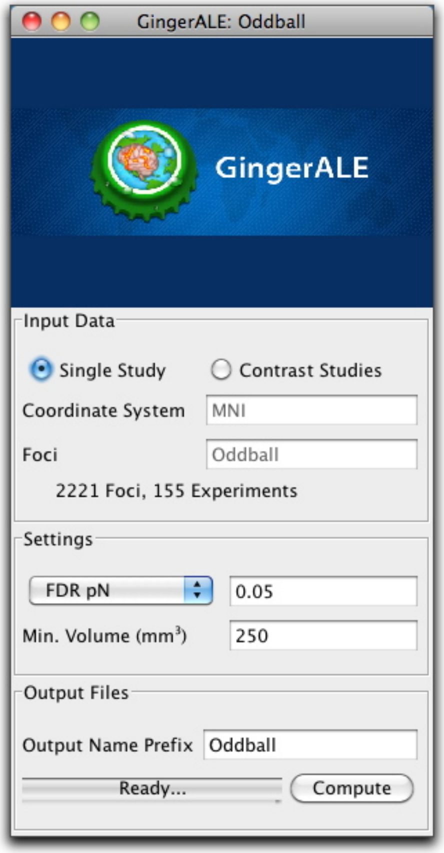

Figure 1. GingerALE Interface.

To carry out these 3 steps, click on "Compute", and GingerALE will write out a separate image for the unthresholded ALE values corresponding to your foci file, the *P* values at each voxel, and a final thresholded ALE map. Only voxels that were found to be statistically significant are assigned a value in this map. The value that is written out is the computed ALE value. The thresholded map is output in .nii format and can be read by a number of functional neuroimaging software packages.

Viewing Your Results

Once the thresholded map has been created, you'll need an anatomical underlay in order to view the meta-analysis results. We distribute two templates in Talairach space (one general file and one to be used specifically by AFNI) and one MNI template on GingerALE's website (www.brainmap.org/ale). Although our .nii files are compatible with most image viewing software, we suggest using Mango to view your meta-analysis results (www.ric.uthscsa.edu/mango). For meta-analyses performed in Talairach space:

a) Download and open Mango.

b) Open → Open Image → select the Talairach template file (Colin1.1.nii) or the MNI template file (Clin27_T1_seg_MNI.nii) that are available on www.brainmap.org/ale, depending on the space in which your meta-analysis was performed.

c) In the brain image that pops up, click on File → Add Overlay → and select the *_pN05.nii image that you created in the penultimate step of GingerALE. This overlays your functional meta-analysis results on top of the anatomical template.

d) Click on Edit → Update Image Range (very important!)

e) To change the color map, go to the smaller rectangular window and click on the red box on the left side, move your cursor down to the next red box, move to the side text box that pops up, move to "Color Table", then click on your preferred color option (Red-to-Yellow and Spectrum are good for ALE results).

f) Moving your cursor throughout the brain will move you through space. Pressing the spacebar will change the orientation of the biggest image (axial, coronal, sagittal).

g) To view anatomical labels of your current location in brain space, as well as the coordinates, select the button with a graph icon just to the right of the button with a "T" icon in the Mango small window, move your cursor down until the globe icon is selected, then select your desired anatomical atlas from the dialog window that appears. Anatomical labels and coordinates will then appear in top of the small Mango window.

To get the ALE values for all voxels (even ones not found to be significant), you'll need to open the file created in the first step of the ALE meta-analysis process (*_ALE.nii).

Subtraction Analysis

To compare two different sets of foci and examine them for statistically significant differences in convergence, you must first run the separate ALE analyses on the two foci files. Then, create a combined text file in which foci from both files are merged and run this "pooled" analysis – make sure that experiments that appear in both sets of foci are only reported once in the pooled text file. Once the pooled analysis is complete, you will need the 3 thresholded ALE files that were create in each of those analyses (*_pN05.nii).

To carry out a subtraction analysis, first select the "Contrast Studies" radio button in the main GingerALE window. Open the thresholded ALE image created from the first foci file from the main menu item: File → Open ALE Image 1. Then, open the thresholded ALE image created from the second foci file: File → Open ALE Image 2. Then, open the thresholded results of the pooled ALE analysis: File → Open Pooled ALE Image. Once all of the required data sets are loaded, make sure the appropriate parameters are selected for the number of permutations, FDR pN value, minimum volume extent, and output file prefix. Then click Compute.

Subtraction results are processed differently than a simple ALE analysis of a single foci file since it is difficult to interpret the subtraction of two sets of ALE images (e.g., significant areas can have a subtraction value of zero). Thus, z score images are saved as output for subtraction analyses, and the thresholded image and cluster analysis are based on these z score values.

Citing GingerALE

If you use GingerALE in your research, please acknowledge our previous work in any resultant publication:

Eickhoff SB, Laird AR, Grefkes C, Wang LE, Zilles K, Fox PT. Coordinate-based activation likelihood estimation meta-analysis of neuroimaging data: A random-effects approach based on empirical estimates of spatial uncertainty. Hum Brain Mapp 30, 2907-2926, 2009.

Laird AR, Fox M, Price CJ, Glahn DC, Uecker AM, Lancaster JL, Turkeltaub PE, Kochunov P, Fox PT. ALE meta-analysis: Controlling the false discovery rate and performing statistical contrasts. Hum Brain Mapp 25, 155-164, 2005.

Turkeltaub PE, Eden GF, Jones KM, Zeffiro TA. Meta-analysis of the functional neuroanatomy of single-word reading: Method and validation. NeuroImage 16, 765-780, 2002.

Main Menu Items

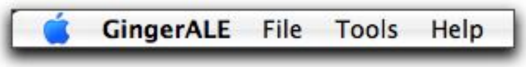

Figure 2. The Main Application Menu.

1. GingerALE Menu Items

About GingerALE: This menu item contains basic information about GingerALE, such as the homepage, version number, and copyright date.

Preferences (Fig. 3): The menu item addresses settings that are relevant to performing ALE meta-analyses. This information is divided into three sections: Mask Options, Default Values, and Output Files.

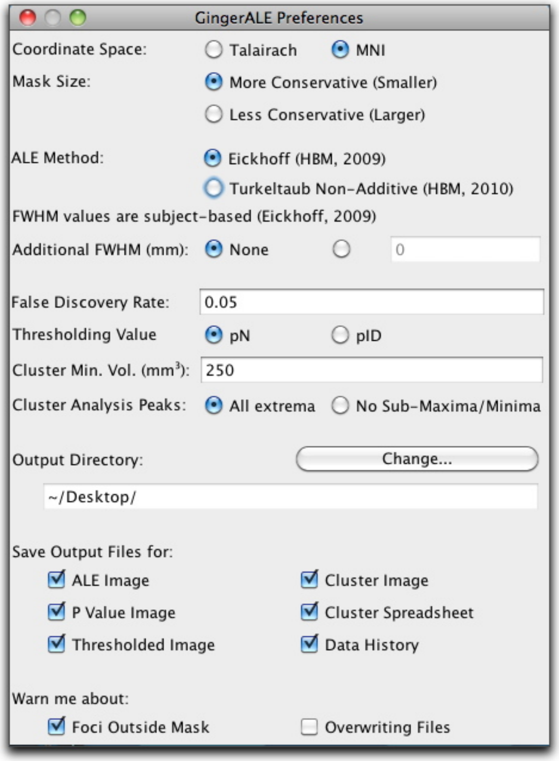

Figure 3. GingerALE Preferences.

- **Coordinate Space:** A radio button is available to select which standard space the meta-analysis should be performed in: Talairach or MNI.

- **Mask Size:** When a foci file is opened, the coordinates are compared against a mask defining the outer limits of Talairach (or MNI) space. A pop-up window will appear if any of your coordinates are located outside of this mask. The ALE analysis will proceed after this step without any intervention on your part. However, any coordinates located outside of this mask will not be omitted from subsequent analysis and might possibly yield strange activations on the border of your mask that do not appear to have a center of mass.

  Normally, finding coordinates outside of the mask will occur for less than 3% of your total foci (we have found this number to be even lower since implementing the Lancaster transform instead of the Brett transform). Finding coordinates located outside of the mask is sometimes due to author error (e.g., missing negative sign, inverted coordinates, etc.). You can often spot this type of error and correct for it manually. For example, if a coordinate is listed as being located in the occipital cortex, but the given y value is positive and extends outside of the Talairach mask, then we recommend that you change the y value from positive to negative before proceeding with ALE.

  Two options are available for your mask size, a smaller mask or a larger mask. Typically, we use the smaller mask for meta-analyses of functional imaging studies. The larger mask is available for VBM meta-analyses because many reported coordinates in these studies are located on the outside of the brain. We slightly enlarged the mask for these meta-analyses so as to include more foci located at the boundaries of Talairach or MNI space.

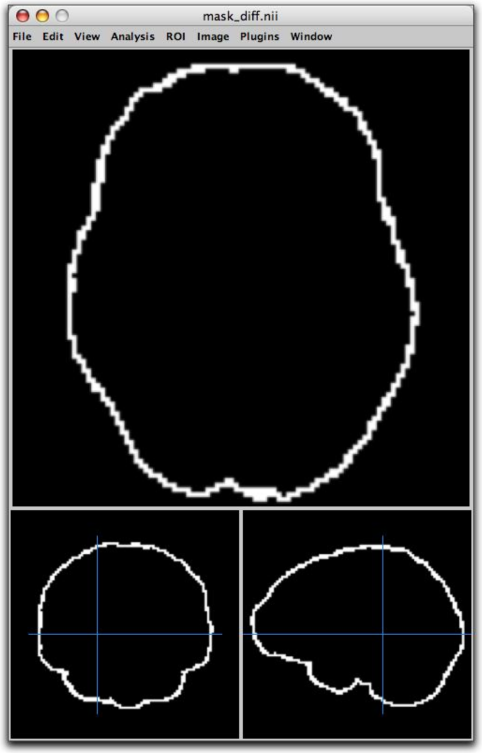

Figure 4. Difference Between Mask Size Options.

  If you have a large number of outlying foci that you do not want omitted from your meta-analysis, then you can select the option of “Less Conservative (Larger)”. This option will slightly increase the default mask size, thus including a wider range of coordinates. An image of the difference between the two mask files for the Talairach template can be seen in Fig.4. In this difference image, the white areas denote the extra voxels included when using the larger (less conservative) mask file. Please note that if you use this larger mask,

some of your resultant ALE clusters may appear to be located outside of the brain when viewed on the Talairach or MNI anatomical templates.

- **ALE Method:** Here, you may choose to use the ALE algorithm described by Eickhoff et al. (2009) or implement the small correction to minimize within-experiment and within-group effects described by Turkeltaub et al. (2011). In addition, since both of these techniques utilize automatically determined FWHM values, we provide an option here for advanced users to implement an additional manual FWHM "fudge factor". Please note that this parameter should be left as "None" for standard analyses.

  Next, you may set default values for the False Discovery Rate, pN or pID, and minimum cluster volume (mm^3). The computation for the false discovery rate will yield two *P* value thresholds. The RIC generally uses thresholds returned by pN. If you prefer pID, you can set it as the default thresholding value in the Preferences. See Genovese et al. (2002) for more details.

  You may also choose if you want the coordinates of any resultant ALE clusters to be reported for all submaxima in a single ALE cluster ("All extrema") or only one coordinate for the maximum ALE statistic in that cluster ("One extrema"). Choosing the former option is very useful for large ALE clusters that extend over many different areas of the brain.

- **Output Files**: Lastly, you may specify the output directory for all of your processed ALE files, which files you want to output, and what your preference is for pop-up windows about boundary foci and overwriting files.

  A number of files may be written to output during the ALE procedure. We recommend that all of these options be checked.

  ALE Image (*_ALE.nii) = contains the unthresholded ALE values, one computed at every voxel in the brain
  P Value Image (*_P.nii) = contains each voxel's *P* value, corrected for multiple comparisons using FDR
  Thresholded Image (*_pN05.nii) = ALE maps thresholded at a given α value; **this is the final image output.**
  Cluster Image (*_clust.nii) = thresholded ALE map, each cluster given an integer value.
  Data History (*_clust.txt) = reports the analysis parameters and the output of the cluster analysis in text format
  Cluster Spreadsheet (*_clust.xls) = excel doc of cluster analysis on thresholded ALE map

  In the data history and cluster spreadsheet files, the cluster analysis reports a variety of information. In the cluster spreadsheet, you will see 10 columns of information. From left to right these are:

  (1) cluster number
  (2) volume of cluster in mm^3
  (3-5) x,y,z values of the weighted center of mass of the cluster
  (6) maximum ALE value observed in the ALE cluster
  (7-9) x,y,z values of the location of the maximum ALE value
  (10) Talairach Daemon anatomical label associated with the location of the maximum ALE value.

  All of this information can be found in both the data history and cluster spreadsheet files. The data history file also includes information on the x,y,z values for the extent of each cluster and reported parameters for different stages of the analysis, such as computing the ALE statistic, performing the permutation test, running FDR and thresholding the ALE map.

2. File Menu Items

Open Foci: Hotkey: ⌘-F (Mac) or ctrl-F (PC). This menu item loads in a text file of coordinates into GingerALE. The format for this file should be three columns of numbers (x,y,z coordinates), separated with tabs or spaces. If

you created your foci file in Sleuth, the file starts by indicating the standard brain space, and subsequent experiments will be separated by a line break and delineated by first author name, year, and experiment name ("//" comments these descriptors out so that they will not be read by the ALE algorithm). Between the commented experiment name and the list of coordinates, you should also include a line that details the number of subjects for that group of foci. For example, your foci text file should look like:

```
// Reference=Talairach
// Hui, 2000: Acupuncture vs. Tactile Stimulation, Increases
// Subjects=13
56        -15       50
-59       -18       43
59        -21       21
-50       -18       18

// Li, 2003: Conventional Acupuncture > Rest, Activations
// Subjects=20
-5.12     -82.33    10.8
2.3       -77.71    11.37
15.36     -62.35    7.64
45.18     15.75     15.54

// Li, 2003: Electro-Acupuncture 2 Hz > Rest
// Subjects=20
2.28      -77.88    13.15
-6        -68.35    12.11
-56.89    -63.43    11.72
49.74     15.21     20.97
```

Open ALE Image 1: Hotkey: ⌘-1 (Mac) or ctrl-1 (PC). Opens an ALE image that corresponds to the first foci file analyzed in a subtraction analysis to test two sets of foci for statistically significant differences.

Open ALE Image 2: Hotkey: ⌘-2 (Mac) or ctrl-2 (PC). Opens an ALE image that corresponds to the second foci file analyzed in a subtraction analysis.

Open Pooled ALE Image: Hotkey: ⌘-3 (Mac) or ctrl-3 (PC). Opens an ALE image that corresponds to the first and second foci files merged and analyzed together.

Save Data History: Hotkey: ⌘-S (Mac) or ctrl-S (PC). This menu item allows you to save a text output that summarizes your ALE meta-analysis at any point in the procedure.

Clear Data: This menu item clears your foci from GingerALE.

3. Tools Menu Items

Export Foci Image: This menu item creates an .nii image of your foci file. In this image, each coordinate point is assigned a value. **No blurring** of the coordinate points is performed in this export – this step is simply intended as a way to view your coordinates in standard space. The value assigned to each coordinate point matches the experiment number of your foci file. Remember, different experiments are defined in a foci file simply by including a line break between the groups of foci. By assigning values in this way, it is easy to set each experiment number to a different color in your image viewer so that you can identify the paper and experiment for each coordinate point as you scroll through the brain. If 2 identical coordinate locations are included in

different experiments, then the value assigned to that voxel will be n+1, where n equals the number of total experiments. This is done so that these duplicate coordinates can be seen on the resultant output image.

Convert Foci: This menu item uses a dialog window as seen in Fig.5 to guide you through the conversion of your coordinates from MNI space to Talairach space and vice versa. You are given options for selecting your input file of coordinates, the transform you would like to use, and the name and location of your output file.

There are 8 coordinate transforms included in GingerALE:

The first three transforms convert coordinates from MNI space to Talairach space using the Lancaster transform, icbm2tal. This transform is broken into 3 options, based on what software you used for spatial normalization of your data (SPM, FSL, or Other):

(1) MNI (SPM) to Talairach
(2) MNI (FSL) to Talairach
(3) MNI (Other) to Talairach

The second three transforms perform the corresponding transforms from Talairach space to MNI space using the Lancaster transform. Again, this transform is broken into 3 software options:

(4) Talairach to MNI (SPM)
(5) Talairach to MNI (FSL)
(6) Talairach to MNI (Other)

The last 2 transforms are reproductions of the Brett transform, mni2tal. Two options are given for the Brett transform, one for converting from MNI space to Talairach space, and the other for converting from Talairach space to MNI space:

(7) Brett: Talairach to MNI
(8) Brett: MNI to Talairach

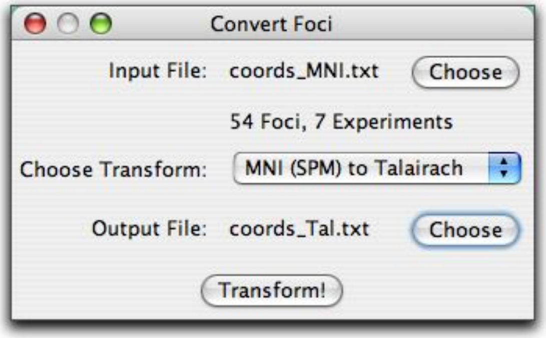

Figure 5. Transforming Coordinates: MNI and Talairach Spaces.

Although the BrainMap database no longer supports use of the Brett transform, we feel it is still important that we include it in our software. If one of the studies included in your meta-analysis generated its coordinates by using SPM for spatial normalization and published those coordinates after conversion using the Brett transform, then we recommend that you “un-Brett” the published coordinates using the above transform “Brett: Talairach to MNI” and then proceed with the Lancaster transform “MNI (SPM) to Talairach”. This will correctly move your coordinates into the Talairach space.

4. Help Menu Items

Check for Updates: This menu item will check the BrainMap website to see if you have the latest version of GingerALE.

Show Manual: This menu item will show the current manual for GingerALE (this document). An internet connection is necessary for this menu option.

Show Read Me: This menu item will show the current readme file for GingerALE. The readme file contains information about installation and version changes. An internet connection is necessary for this menu option.

Show License: This menu item will show the current license information for GingerALE. An internet connection is necessary for this menu option.

References

Eickhoff SB, Laird AR, Grefkes C, Wang LE, Zilles K, Fox PT. Coordinate-based activation likelihood estimation meta-analysis of neuroimaging data: A random-effects approach based on empirical estimates of spatial uncertainty. Hum Brain Mapp 30, 2907-2926, 2009.

Genovese CR, Lazar NA, Nichols TE. Thresholding of statistical maps in functional neuroimaging using the false discovery rate. Neuroimage 15, 870-878, 2002.

Laird AR, Robinson JL, McMillan KM, Tordesillas-Gutierrez D, Moran ST, Gonzales SM, Ray KL, Franklin C, Glahn DC, Fox PT, Lancaster JL. Comparison of the disparity between Talairach and MNI coordinates in functional neuroimaging data: Validation of the Lancaster transform. Neuroimage 51, 677-683, 2010.

Laird AR, Fox M, Price CJ, Glahn DC, Uecker AM, Lancaster JL, Turkeltaub PE, Kochunov P, Fox PT. ALE meta-analysis: Controlling the false discovery rate and performing statistical contrasts. Hum Brain Mapp 25, 155-164, 2005.

Lancaster JL, Tordesillas-Gutierrez D, Martinez M, Salinas F, Evans A, Zilles K, Mazziotta JC, Fox PT. Bias between MNI and Talairach coordinates analyzed using the ICBM-152 brain template. Hum Brain Mapp 28, 1194-1205, 2007.

Turkeltaub PE, Eickhoff SB, Laird AR, Fox M, Wiener M, Fox P. Minimizing within-experiment and within-group effects in activation likelihood estimation meta-analyses. Hum Brain Mapp, In Press.

Turkeltaub PE, Eden GF, Jones KM, Zeffiro TA. Meta-analysis of the functional neuroanatomy of single-word reading: Method and validation. NeuroImage 16, 765-780, 2002.
